# sp-Hybridized Seesaw Ge^0^ Complexes via Germylone-to-Seesaw Isomerization in a Four-Electron Cyclic N_2_Ge_2_ Ligand

**DOI:** 10.1021/acs.inorgchem.5c02120

**Published:** 2025-09-12

**Authors:** Wei-Ting Kuo, Cian-Wei Yang, Gou-Tao Huang, Yu-Te Wey, Fan-Shan Yang, Hsien-Cheng Yu, Jen-Shiang K. Yu, Yi-Chou Tsai

**Affiliations:** † Department of Chemistry, 34881 National Tsing Hua University, Hsinchu 300313, Taiwan, Republic of China; ‡ Department of Biological Science and Technology, Institute of Bioinformatics and Systems Biology and Center for Intelligent Drug Systems and Smart Bio-devices (IDSB), 541573 National Yang Ming Chiao Tung University, Hsinchu 300, Taiwan, Republic of China

## Abstract

We report the first examples of two linear trigermanium complexes (μ-Ge)­(κ^2^-**N**_**2**_ **Ge**_**2**_^**Ar**^) (Ar = 2,4,6-Me_3_C_6_H_2_ (**4**), 2,6-Et_2_C_6_H_3_ (**5**)), in which each central Ge^0^ atom displays a seesaw geometry enforced by a four-electron cyclic **N**_**2**_ **Ge**_**2**_^**Ar**^ ligand. The pyridine-stabilized sp-hybridized Ge^0^ center is a four-electron donor and two terminal germylenes are electron-acceptors. Comparative studies show that the N_2_Ge_2_ scaffold uniquely stabilizes the Ge^0^ atom. This work not only introduces a new class of main-group seesaw complexes but also demonstrates ligand-driven control over hybridization and donor–acceptor dynamics. Compound **5** underwent one-electron reduction, followed by ligand rearrangement and dimerization, to afford a novel hexanuclear homounivalent germanium cluster, **10**, featuring a snake-like Ge_6_ core. In **10**, each Ge atom in the central Ge^I^–Ge^I^ unit is solely stabilized one amido ligand, while each of the remaining four Ge atoms is supported by one amido and one pyridyl donor. The isolation of a bulkier analogue (**11**) and a heteronuclear Ge_4_Sn_2_ cluster (**16**) further underscores the crucial role of ligand sterics in stabilizing these assemblies. These findings expand the structural diversity of low-valent group 14 compounds and establish a new paradigm for constructing multinuclear tetrel clusters.

## Graphical abstract

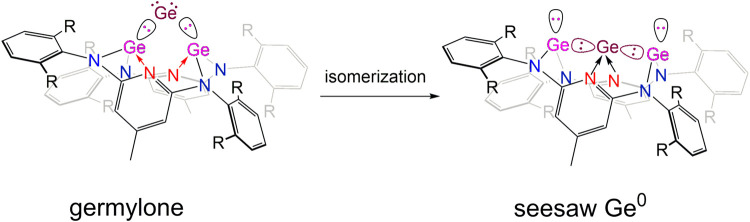

## Introduction

The seesaw (or sawhorse) molecular geometry is an unusual coordination arrangement that arises in molecules with a steric number of five, typically involving a central atom bonded to four substituents and featuring one lone pair. Such geometries are most commonly observed in hypervalent compounds of group 16, 17, and 18 elements, such as SF_4_, SeF_4_, TeCl_4_, [ClF_4_]^+^, and XeO_2_F_2_. The prevalence of the seesaw geometry among these elements can be attributed to hypervalency and expanded octet effects, which enable the central atom to adopt nontetrahedral geometries.^1^

Zero-valent germanium (Ge^0^) compounds (germylones) have recently emerged as an intriguing class of main-group species (Figure 1a–d).^2–19^ A germylone is defined as a Ge^0^ complex stabilized by two σ-donating ligands, with the germanium center retaining two lone pairs.^20–30^

**Figure 1:**
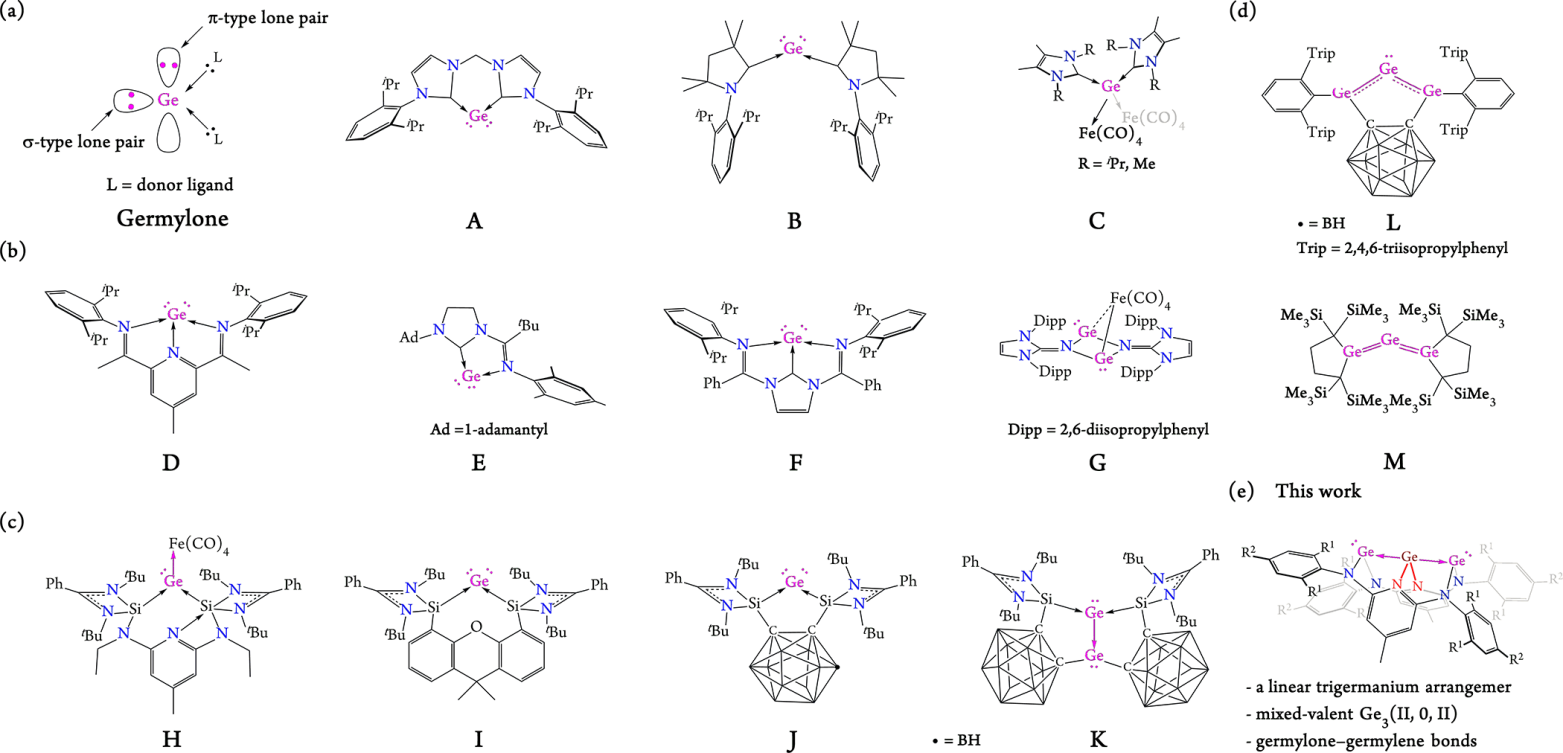
Characterized germylones supported by (a) N-heterocyclic carbenes (NHCs), (b) N-donors and imine-NHCs, (c) bis­(DSSi) (DSSi = donor-stabilized silylene), and (d) bis­(germylene), and their Lewis adducts (**C**, **H**, **G** and **K**). (e) Present work.

These monatomic zerovalent group 14 species exhibit bridging bidentate coordination behavior, where the two lone pairs can be donated to two metal fragments, forming tetrahedral coordination environments.^31–36^ Such interactions have led to the development of germylones as ligands in coordination chemistry, stabilizing the main-group and transition metals of various electronic configurations. However, all previously reported Lewis adducts exhibit tetrahedral-like geometries upon metal coordination. The possibility of the Ge^0^ atom featuring an alternative bonding mode has remained unexplored.

To address this challenge, we sought to develop a new strategy for stabilizing a two-coordinate Ge^0^ center. In our previous work, we reported the synthesis of a digermylene­(II) complex stabilized by two bulky terdentate 2,6-diamidopyridine ligands (Ge_2_(μ-κ^1^:κ^2^-DAP^Dipp^)_2_) (DAP^Dipp^ = 2,6-(DippN)_2_-4-CH_3_C_5_H_2_N; Dipp = 2,6-^*i*^Pr_2_C_6_H_3_) (**1**) (Figure 2).^37^ Each divalent Ge atom is three-coordinate and is supported by two amido and a pyridyl ligand, rendering it a N-donor stabilized four-membered-ring cyclic germylene (DSGe). Therefore, these two DSGes are expected to serve as good σ-donors and **1** (bis­(DSGe)) can thus be used as a bidentate ligand to stabilize a Ge^0^ atom.^5^ Another interesting structural feature in **1** is its possible structural flexibility. Due to the four-membered ring strain, the pyridyl N donors can readily decoordinate from the Ge atoms of two DSGes and lead to the formation of two two-coordinate germylenes, by which the structure of **1** will transform into a boat conformation and the electronic nature of two Ge atoms are reversed from electron-donors to electron-acceptors. As a result, an unusual four-electron cyclic dipyridyl-digermylene ligand is formed, which contains two N-donors and two Ge-acceptors (Ge_2_(μ-κ^1^:κ^1^-DAP^Dipp^)_2_ (**N**_**2**_ **Ge**_**2**_^**Dipp**^)) (Figure 2). The pocket in the boat framework of **N**_**2**_ **Ge**_**2**_^**Dipp**^ could be suitable for accommodating a Ge^0^ atom in an unconventional coordination environment, where two pyridyl N-donors are used to support a Ge^0^ atom, whose two lone pairs stabilize two outer divalent Ge atoms via coordination.

**Figure 2:**
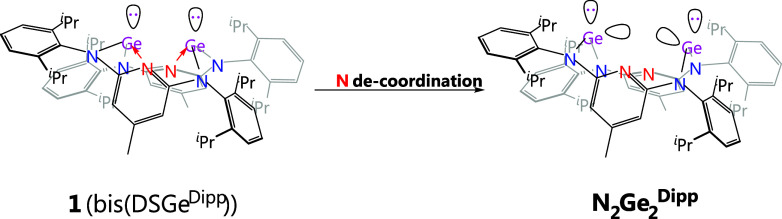
A cyclic four-electron dipyridyl-digermylene ligand (**N**_**2**_ **Ge**_**2**_^**Dipp**^) is used to support a Ge^0^ atom, where the ligand is derived from **1** (bis­(DSGe^Dipp^)) via decoordination of two pyridyl N-donors from the two Ge atoms.

Inspired by the success of Driess‘ bidentate bis­(DSSi) ligand (DSSi: donor-stabilized silylene) in stabilizing a Ge^0^ atom (**H**, **I**, **J**, and **K** in Figure 1c),^5^ we demonstrate that the smaller analogues (Ge_2_(μ-κ^1^:κ^2^-DAP^Ar^)_2_) of **1**, serve as ideal **N**_**2**_ **Ge**_**2**_^**Ar**^ [Ar = Mes, Dep, where Mes is 2,4,6-Me_3_C_6_H_2_ and Dep is 2,6-Et_2_C_6_H_3_] platforms for stabilizing a Ge^0^ center (Figure 1e), wherein the Ge^0^ atom is supported by two pyridyl N-donors and donates both of its lone pairs to two Ge^II^ acceptors. This arrangement removes the lone pairs from the Ge^0^ center, leading to the first example of seesaw compounds of a group 14 element. The three germanium atoms are aligned into a linear configuration, highlighting the unusual electronic structure of the Ge^0^ centers. The formation mechanism is realized by density functional theory calculations, which also reveals that the central Ge^0^ atom exhibits sp hybridization, rendering it to donate its lone pairs to the adjacent Ge^II^ centers. This bonding mode effectively stabilizes the Ge^0^ centers, while enforcing a seesaw geometry Given that monatomic Ge^0^ species typically form tetrahedral or pseudotetrahedral coordination environments upon coordination,^31–36^ our approach provides a fundamentally new strategy for main-group element stabilization and geometric control.

Beyond its unusual geometry, the unique electronic structure of the seesaw Ge^0^ center imparts distinctive reactivity. Notably, these species exhibit intriguing redox behavior: oxidation with GeCl_4_ yields a tetranuclear Ge^II^ complex, while reduction leads to the formation of novel hexagermanium clusters featuring unprecedented snake-like Ge_6_ cores with diverse Ge–Ge bonding motifs. These reactivity studies underscore the potential of the trigermanium species as a versatile building block for constructing multinuclear tetrel assemblies, demonstrating that our approach effectively modulates both the bonding and chemical behavior of low-valent group 14 species.

## Results and Discussion

The bis­(DSGe^Dipp^) compound, Ge_2_(μ-κ^1^:κ^2^-DAP^Dipp^)_2_ (**1**), features an unusual eclipsed configuration in the solid state.^37^ However, attempts to employ **1** as a bidentate ligand for Ge^0^ coordination were unsuccessful, likely due to the pronounced steric hindrance of the bulky DAP^Dipp^ substituents. To address this, two sterically less demanding bis­(DSGe^Ar^) analogues, Ge_2_(μ-κ^1^:κ^2^-DAP^Ar^)_2_ [Ar = Mes (**2**), Dep (**3**)], were synthesized in good yields (Scheme 1).

**Scheme 1:**
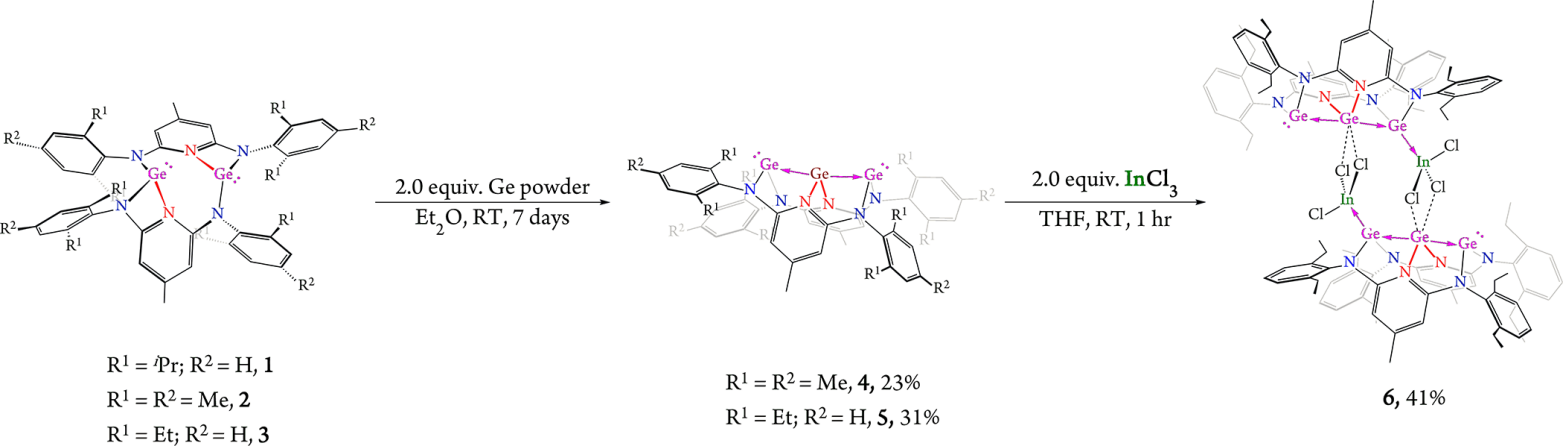
Synthesis of Two Germylone-Stabilized Linear Trigermanium Compounds **4** and **5**, and Lewis Acid–Base Coupling of **5** with InCl_3_ to Afford Indium Adduct **6**

Single-crystal X-ray diffraction analysis revealed that **2** and **3** are nearly isostructural, both adopting bent geometries in which each Ge center is three-coordinate, bonded to one pyridyl nitrogen and two amido donors (Figures S1 and S2). The coordination environment around each Ge atom is approximately trigonal pyramidal, consistent with the presence of a stereoactive lone pair. The Ge–N_amido_ bond lengths are ca. 0.1 Å shorter than the Ge–N_pyridine_ distances, indicating a greater localization of negative charge at the amido nitrogen atoms. Notably, the long Ge···Ge separations in **2** (3.1410(4) Å) and **3** (3.1775(11) Å) suggest the absence of a direct Ge–Ge bonding interaction.

Theoretical analyses including frontier molecular orbital (FMO), natural bond orbital (NBO), and electron localization function (ELF) calculations (Figures S57, S60, and S67) suggest that compounds **2** and **3** have the potential to act as chelating ligands. Accordingly, both were reacted with elemental germanium to afford two trigermanium complexes, (μ-Ge)­(κ^2^-**N**_**2**_ **Ge**_**2**_^**Ar**^) [Ar = Mes (**4**), Dep (**5**)] in 23 and 31% yield (Scheme 1), respectively. Both **4** and **5** are soluble in *n*-hexane, benzene, toluene, diethyl ether, and THF. The ^1^H NMR spectra of **4** and **5** (in C_6_D_6_) exhibit a singlet for the pyridyl meta-protons at δ = 5.36 and 5.24 ppm, respectively, consistent with a symmetric environment around the Ge_3_ core. The solid-state molecular structures of **4** and **5**, determined by single-crystal X-ray diffraction (Figures S3 and 3a), reveal an unprecedented linear arrangement of the Ge_3_ core, with Ge–Ge–Ge bond angles of 177.83(2)° (**4**) and 176.61(2)° (**5**). The structure around the central Ge atoms adopt an unusual seesaw geometry,^38^ while the terminal Ge atoms adopt trigonal pyramidal coordination. The pyridyl N donors coordinate to the central Ge^0^ atoms, by which each of the terminal germylene atoms is ligated by the central Ge^0^ atom in addition to two amido N donors. To the best of our knowledge, **4** and **5** represent the first structurally characterized trigermanium complexes in which each Ge^0^ center is supported by two relatively weak σ-donating pyridyl ligands,^2–6^ exhibiting a seesaw coordination environment. This is in stark contrast to the previously reported Lewis adducts,^31–36^ which typically feature a tetrahedral geometry around the group 14 element and coordinate to two metal fragments via donor–acceptor interactions.

**Figure 3:**
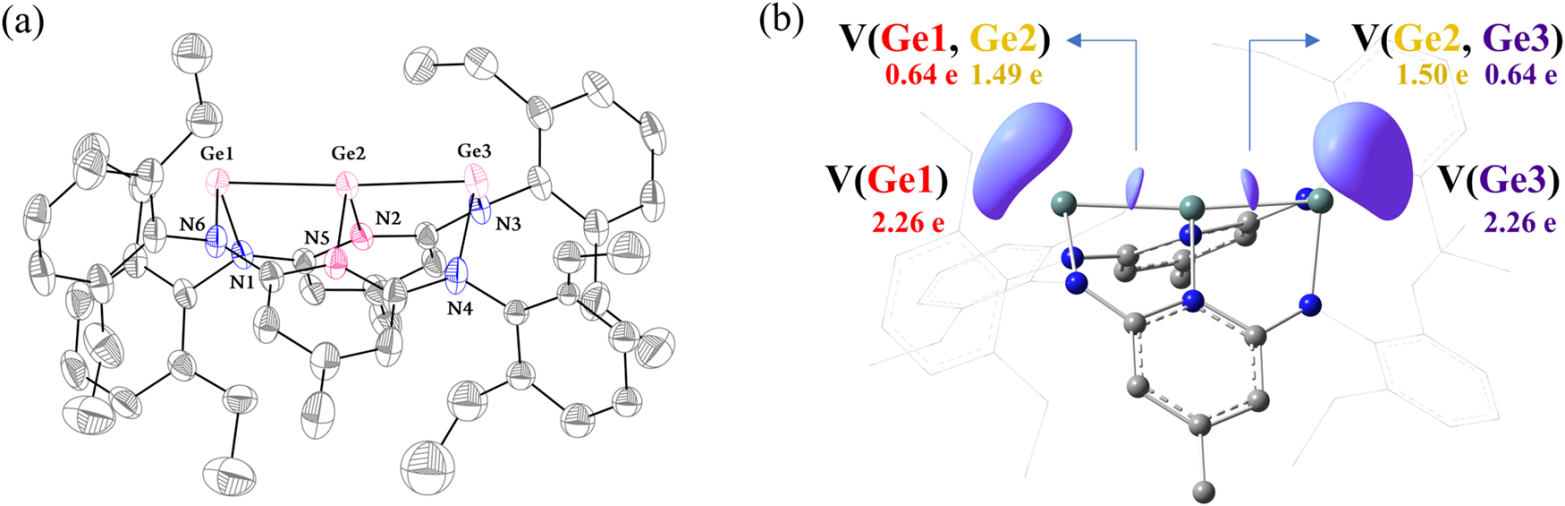
(a) The solid-state molecular structure of **5** with thermal ellipsoids at 50% probability. The hydrogen atoms have been omitted for clarity. Selected bond lengths (Å) and angles (°) Ge1–Ge2, 2.5042(4); Ge2–Ge3, 2.4986(4); Ge1–N1, 2.023(2); Ge1–N6, 1.965(2); Ge2–N2, 1.968(2); Ge2–N5, 1.965(2); Ge3–N3, 1.966(3); Ge3–N4, 2.026(3); Ge1–Ge2–Ge3, 176.609(19); N1–Ge1–N6, 100.11(10); N2–Ge2–N5, 101.00(10); N3–Ge3–N4, 99.37(11); Ge2–Ge1–N1, 80.61(7); Ge2–Ge1–N6, 84.14(7); N3–Ge3–Ge2, 84.07(7); N4–Ge3–Ge2, 80.65(7). (b) ELF plots of **5**. The ELF function of η­(**r**) = 0.8 is shown around Ge.

Both **4** and **5** show an eclipsed conformation along the Ge–Ge–Ge axes, as evidenced by N–Ge–N angles between 98.82(11)° and 101.00(10)°, indicating that each terminal Ge atom contains a stereoactive lone pair. The Ge–Ge bond lengths in **4** and **5** are nearly equivalent at approximately 2.50 Å, and notably shorter than that of the germylone–germylene-paired Ge_2_ species (LSi)_2_Ge_2_ (**K**) (2.599(2) Å) (LSi = PhC­(^*t*^BuN)_2_Si–C,C’-C_2_B_10_H_10_).^11^ This bond length difference can be rationalized by the involvement of two lone pairs on the central Ge atom into Ge→Ge dative interactions with the terminal Ge atoms in **4** and **5**, which reduce the bonding pair-lone pair repulsions between the central Ge atom and two terminal Ge atoms. Moreover, the Ge–Ge bonds in both complexes (**4** and **5**) are also shorter than those found in the *trans*-bent and *gauche* digermylenes (2.506(1)-2.709(1) Å)^39–46^ and yet significantly longer than those in LGe^I^Ge^I^L (ca. 2.20–2.28 Å),^47–50^ the bis­(germylene)-stabilized germylone (**L**) (2.369 (1) and 2.357(1) Å)^15^ and the trigermaallene (**M**) (2.321(2) and 2.330(2) Å).^16^

To gain further insight into the electronic structure of the trigermanium core, DFT calculations were performed on compound **5**. The optimized geometry closely matched the experimentally determined structure (Table S5). Natural bond orbital (NBO) analysis (Figure S61) revealed that each terminal Ge atom (Ge1 and Ge3) carries a lone pair of electrons, with respective occupancies of sp^0.24^. In contrast, the central Ge atom (Ge2) lacks a lone pair and instead donates electron density to the terminal Ge atoms via two Ge→Ge dative bonds, described as polarized σ-bonds (35% Ge1­(sp^9.61^) + 65% Ge2­(sp^1.25^); 65% Ge2­(sp^1.27^) + 35% Ge3­(sp^9.65^)), involving two sp-hybridized orbitals on Ge2. This is noteworthy because heavy main-group elements (*n* > 2) typically are reluctant to adopt sp hybridization due to the significant energy gap between their n*s* and n*p* orbitals.^51–54^ The unusual sp hybridization at the Ge2 center arises from the rigid boat-like conformation of the **N**_**2**_ **Ge**_**2**_ ligand framework and the steric repulsion between the two DAP^Ar^ ligands, the latter of which enforces large N_pyridyl_–Ge2–N_pyridyl_ bond angles. Consequently, **4** and **5** represent the first trigermanium complexes with the central Ge^0^ atom featuring two lone pairs residing in two sp-hybridized orbitals. This bonding picture is further supported by ELF analysis (Figures 3b and S68). Highly localized electron basins were found on the terminal Ge atoms [V­(Ge1) = V­(Ge3) = 2.26 e], confirming the presence of lone pairs. In the Ge–Ge bonding basins [V­(Ge1,Ge2) and V­(Ge2,Ge3)], the central Ge atom contributes the majority of the electron density (1.49 and 1.50 e, respectively), while Ge1 and Ge3 contribute only 0.64 e each, consistent with a donor–acceptor bonding model.

To further validate this bonding description, extended transition state-natural orbitals for chemical valence (ETS-NOCV) calculations were carried out.^55^ The results revealed that electron donation from the central Ge to the two terminal Ge atoms accounts for over 79% of the total orbital interaction energy (ΔE_orb_ = – 450.65 kcal/mol), with the two major contributions being Δρ_1_ = −298.96 kcal/mol and Δρ_2_ = −58.22 kcal/mol (Table S7). These findings provide strong computational evidence for a Ge^0^ center bonding scheme involving two polarized Ge→Ge σ-dative bonds.

The formation mechanism of **4** and **5** was investigated via DFT calculations, using the model system [Ge­(μ-κ^1^:κ^2^-DAP^Me^)]_2_ (**3** ^**Me**^) and a Ge atom as the reactants. All transition states were confirmed by the intrinsic reaction coordinate (IRC) analyses (Figures S73 and S74). As shown in Figure 4, as expected, the initial step involves the coordination of a Ge atom to **3** ^**Me**^, forming an intermediate, germylene-supported germylone (μ-Ge)­(κ^2^-**N**_**2**_ **Ge**_**2**_^**Me**^) (**5A**). Similar to Driess’s DSSi-supported germylones (**I** and **J**) (Figure 1c), the three Ge atoms adopt a bent geometry with the Ge–Ge–Ge bond angle of 68.60°. This angle is markedly narrower than those observed in related systems, such as the trigermaallene (**M**) (122.61(6)°),^16^ bis­(germylene)-stabilized germylone (**L**) (82.27(5)°),^15^ and the optimized NHGe-supported germylones (79–90°)^27–30^ (NHGe = *N*-heterocyclic germylene), likely due to the geometric rigidity imposed by the supporting DAP^Me^ ligands. The average Ge–Ge bond length in **5A** is 2.423 Å, comparable with the separations predicted for analogous NHGe-supported germylones (ca. 2.40–2.42 Å).^27–30^ Interestingly, the distance between the two terminal Ge atoms is 2.7035 Å, approximately 0.4 Å shorter than the corresponding nonbonded separations in **2** and **3**, and accompanied by a Wiberg bond index (WBI) of 0.43, indicative of a weak Ge···Ge interaction.

**Figure 4:**
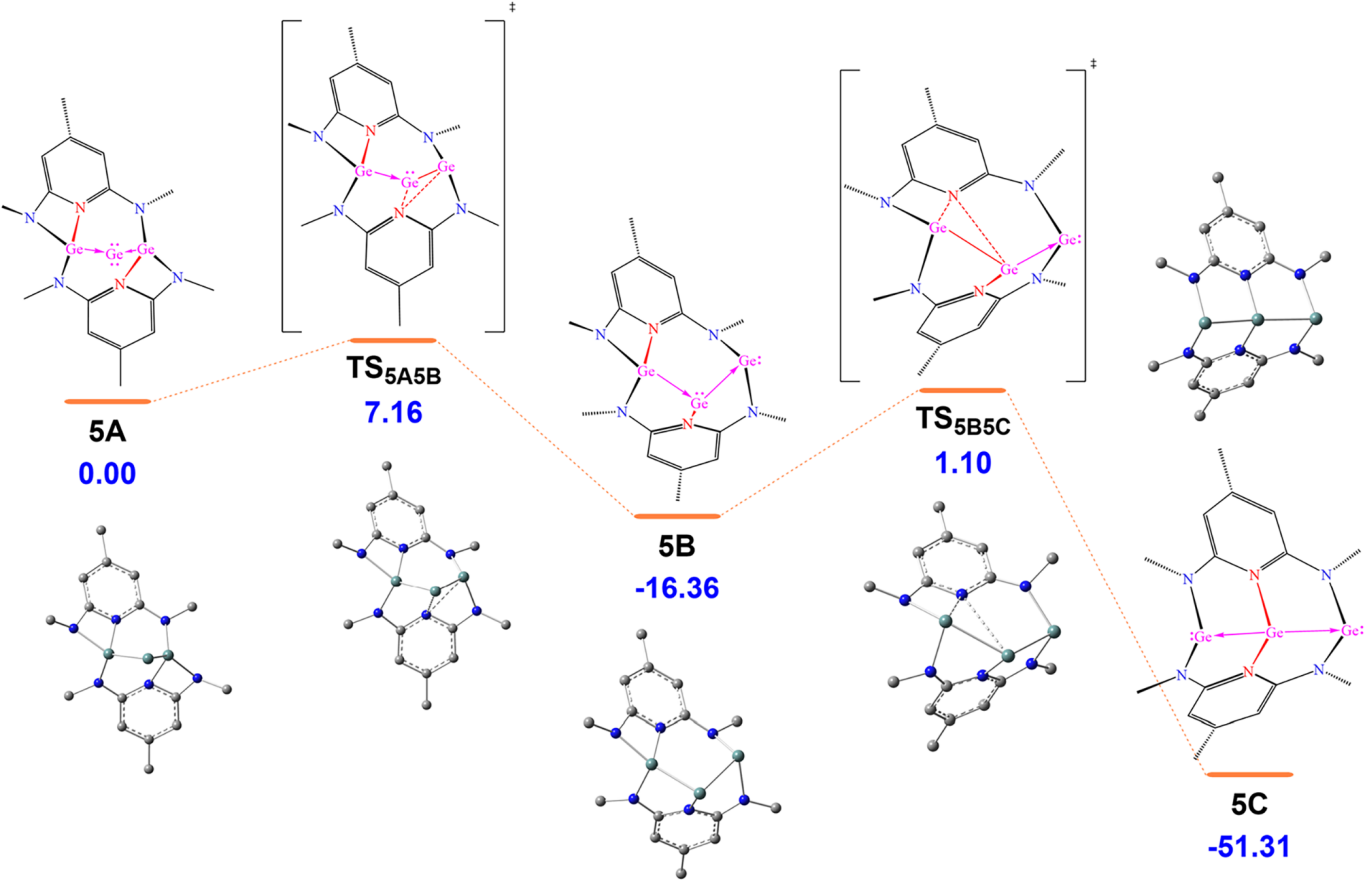
Energy profile for **5A** undergoing intramolecular rearrangement via an intermediate **5B** to give the product **5C**. All free energies in kcal/mol are relative to **5A** at 1 atm and 298.15 K.

Electronic structure analysis via NBO (Figure S64), ELF (Figure S70), and ETS-NOCV (Table S8) revealed that the central Ge atom in **5A** carries two lone pairs of electrons, while no significant ELF basin is observed between the outer Ge atoms. These findings suggest a donor–acceptor framework, with the central Ge^0^ acting as a Lewis acid, rather than forming a delocalized three-center bond. Collectively, **5A** can be described as a germylone with incipient cyclotrigermanylidene character,^56–60^ reflecting a subtle degree of multicenter bonding yet retaining localized lone pairs.

Following its initial formation, intermediate **5A** undergoes a low-barrier intramolecular rearrangement (ΔG^‡^ = 7.16 kcal·mol̅^1^) to afford the intermediate **5B**, and subsequently the final product **5C**. In **5B**, coordination of the central Ge^0^ atom by a pyridyl N donor and one NHGe ligand inverts the remaining NHGe→Ge^0^ donor–acceptor interaction to an NHGe←Ge^0^ bonding mode. These sequential interaction changes from **5A** to **5B** then enable the formation to the final product, **5C**, in which the central Ge^0^ atom is coordinated by two pyridyl N atoms and donates its two lone pairs to two terminal Ge^II^ centers. This transformation is favored by strain release accompanying conversion of the two four-membered [NCNGe] rings in **5A** into four five-membered [GeNCNGe] rings in **5C**, and by electronic effects arising from the stronger σ-donating ability of the pyridyl N atoms than the two NHGe moieties in **5A**.^61–63^ These differences highlight that the transformation of the Ge_3_ core is thermodynamically favorable, as evidenced by a substantial free energy decrease of 51.31 kcal·mol̅^1^. During the rearrangement, the pyridyl N atoms decoordinate from the NHGe groups and recoordinate to the central Ge atom, prompting a reorganization of the donor–acceptor interactions within the Ge_3_ core. Specifically, the Ge atom in **5C** donates both lone pairs to form two Ge→Ge dative bonds to the terminal Ge atoms. As a result, a lone pair becomes localized on each terminal Ge atom in the final structure. This reversal of donor–acceptor polarity is supported by NBO (Figures S65 and S66), ELF (Figures S71 and S72), and ETS-NOCV (Tables S9 and S10) analyses. In intermediate **5B**, the electron density is distributed such that the central and one outer Ge atom each retain a lone pair. In the final product **5C**, however, the central Ge atom lacks any lone pair, while both outer Ge atoms carry localized lone pairs, fully consistent with the bonding picture deduced from the solid-state structure of compound **5**.

To confirm the presence of a lone pair of electrons on each outer Ge atom in **4** and **5**, two equivalents of InCl_3_ were added to **5** in THF, affording the indium adduct [(Cl_3_In)←(μ-Ge)­(κ^2^-**N**_**2**_ **Ge**_**2**_^**Dep**^)]_2_ (**6**) (Scheme 1), which was characterized by X-ray crystallography. Crystals of **6** were obtained under two different conditions: from diethyl ether at – 35 °C and from C_6_D_6_ at room temperature, yielding different crystal systems *P*2_1_/*n* for **6**·Et_2_O and *P*1̅ for **6**·5C_6_D_6_. Despite the differences in packing, both crystals reveal a dimeric aggregation of two [(Cl_3_In)←(μ-Ge)­(κ^2^-**N**_**2**_ **Ge**_**2**_^**Dep**^)] units via intermolecular head-to-tail Ge···Cl dipole–dipole interactions (Figures 5 and S5). In both crystal forms, two Cl atoms of the InCl_3_ unit approach the central Ge atom of the adjacent molecule, consistent with a partial positive charge on the central Ge atom. The Ge···Cl distances [3.3373(8)-3.4724(8) Å] are notably shorter than the sum of the van der Waals radii of Ge and Cl (3.86 Å),^64^ indicating a significant dipolar interaction. The Ge2–Ge1–In1 angles are 134.41(2)° and 137.14(1)°, and the Ge1–In1 bond lengths are 2.6217(4) Å and 2.6383(4) Å in **6**·Et_2_O and **6**·5C_6_D_6_, respectively. Compared to compound **5**, the Ge_3_ core in **6** exhibits distinct geometric distortions, particularly around the Ge1 center bonded to InCl_3_. The N–Ge1–N bond angle increases by approximately 4°, which can be attributed to lone pair donation from Ge1 to InCl_3_, thereby reducing lone pair-bonding pair repulsion at Ge1. Upon coordination to one molecule of InCl_3_, the increased acidity of the central Ge^0^ atom in **6** trigers dimerization. As a result, the other terminal Ge atom is shielded by two neighboring intramolecular aryl groups and the intermolecular InCl_3_ molecule and is thus inaccessible to an InCl_3_ molecule. This is supported by an independent experiment, in which the addition of 20 equiv of InCl_3_ to **5** also leads to the formation of **6** only. Additionally, the Ge1–Ge2 bond length shortens to ca. 2.40 Å, significantly shorter than the Ge2–Ge3 bond (ca. 2.51 Å) and also shorter than that in the germylone–germylene donor–acceptor complex [SiNSi]­Ge→GeCl_2_→Fe­(CO)_4_ (2.4784(7) Å, SiNSi = 2,6-{N­(Et)­Si­(^*t*^BuN)_2_PhC}_2_-pyridine).^9^ This difference highlights the reduction in electron repulsion upon coordination, further supporting the localization of a lone pair on Ge1 in the parent compound.

**Figure 5:**
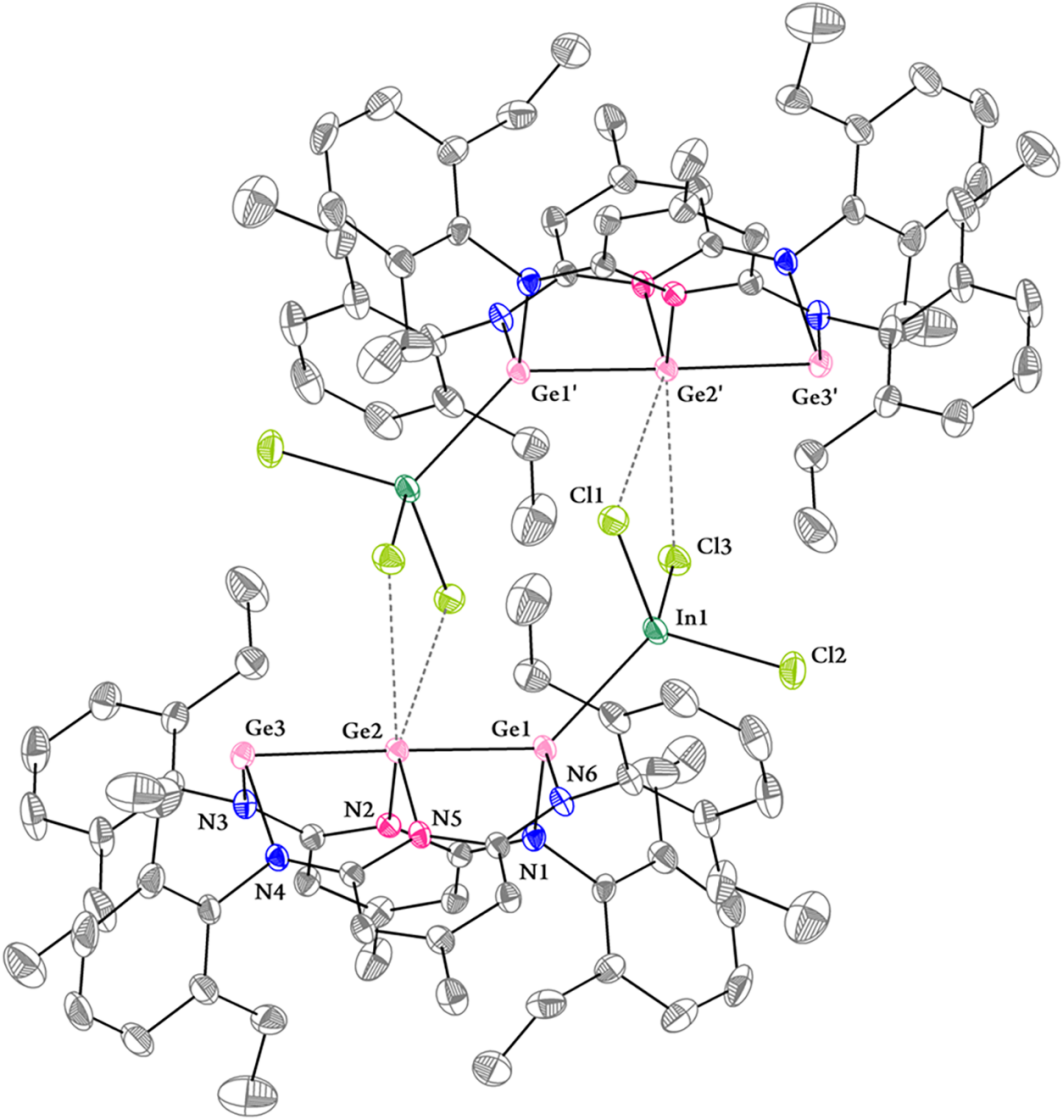
Solid-state molecular structure of **6·Et**_**2**_ **O** with thermal ellipsoids at 30% probability. Hydrogen atoms and Et_2_O were omitted for the sake of clarity. Selected bond lengths (Å) and angles (°): Ge1–Ge2, 2.3987(5); Ge2–Ge3, 2.5031(5); In1–Ge1, 2.6217(4); In1–Cl1, 2.4018(9); In1–Cl2, 2.3580(10); In1–Cl3, 2.3805(10); Ge1–N1, 1.915(3); Ge1–N6, 1.893(3); Ge2–N2, 1.976(3); Ge2–N5, 1.989(3); Ge2′···Cl1, 3.4091(10); Ge2′···Cl3, 3.3514(11); Ge3–N3, 1.973(3); Ge3–N4, 2.043(3); Ge1–Ge2–Ge3, 178.77(2); In1–Ge1–Ge2, 134.405(18); N1–Ge1–N6, 104.69(12); N2–Ge2–N5, 98.92(11); N3–Ge3–N4, 99.18(11); Ge2–Ge3–N3, 83.40(8); Ge2–Ge3–N4, 79.60(7).

Fascinated by the well-documented catenation ability of the tetrel elements,^11,37,65–67^ we sought to explore the potential of compounds **4** and **5** as building blocks for higher-nuclearity germanium clusters since they contain a Ge^0^ atom, which is prone to be oxidized. Reactions of **4** and **5** with GeCl_4_ in Et_2_O or toluene led to the formation of cyclic tetranuclear homodivalent germanium complexes, (GeCl)_4_(μ-κ^1^:κ^1^-DAP^Ar^)_2_ [Ar = Mes (**7**), Dep (**8**)]. The bulkier analogue, (GeCl)_4_(μ-κ^1^:κ^1^-DAP^Dipp^)_2_ (**9**), was synthesized by reacting compound **1** with two equivalents of GeCl_2_·dioxane. Interestingly, KC_8_ reduction of **7** and **8** regenerated compounds **4** and **5** in good yields of 61 and 85% (Scheme 2), respectively. These two-step reduction–oxidation interconversions provide efficient synthetic routes to cyclic oligogermanes, which are typically challenging to access via classical reductive coupling of dichlorogermanes, a method often plagued by low yields.^68,69^ The ^1^H NMR spectra of **7**-**9** are fully consistent with their solid-state structures (*vide infra*). Single-crystal X-ray crystallography of **8** (Figure S7) and **9** (Figure S8) reveals a puckered Ge_4_ ring, with dihedral angles of 27.04(1)° and 27.64(2)°, respectively. The two organic ligands reside on opposite faces of the Ge_4_ ring and adopt an approximately perpendicular orientation. Each ligand bridges two Ge atoms, leading to a tetrahedral coordination geometry at each Ge center, which also bears a terminal chlorido ligand. All Ge–Ge bond lengths are essentially equivalent, ranging from 2.4354(6)-2.4460(4) Å in **8** and 2.4442(10)-2.4677(7) Å in **9**, indicative of delocalized bonding within the Ge_4_ core.

**Scheme 2:**
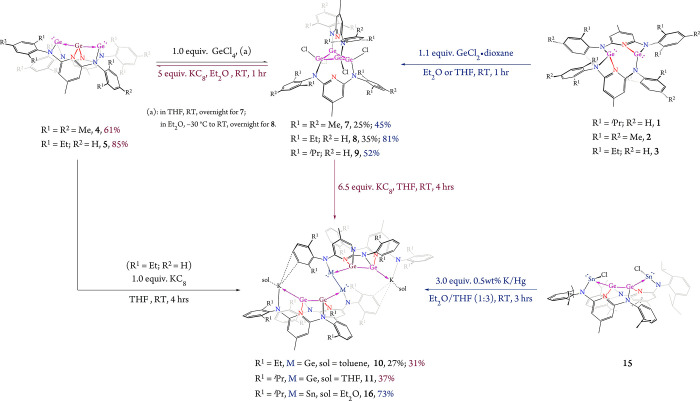
Preparation of the Trigermanium Compounds **4** and **5**, Cyclic Tetagermanium Compounds **7–9**, and Hexagermanium Compounds **10**, **11** and **16**

Given that each of **4** and **5** contains two Ge^II^ centers, we anticipated that they might exhibit interesting reduction behavior. Indeed, KC_8_ reduction of **5** unexpectedly led to the isolation of a hexagermanium cluster, [K­(C_7_H_8_)]_2_Ge_6_(μ_3_-κ^1^:κ^1^:κ^1^-DAP^Dep^)_2_(μ_4_-κ^1^:κ^1^:κ^1^:η^2^-DAP^Dep^)_2_ (**10**) (Scheme 2), which could also be obtained by reducing **8** under similar conditions. Although the corresponding trigermanium species, [Ge_3_(μ_3_-κ^1^:κ^1^:κ^1^-DAP^Dipp^)_2_], was not isolable, presumably due to steric crowding, the bulkier hexagermanium analogue, [K­(THF)]_2_Ge_6_(μ_3_-κ^1^:κ^1^:κ^1^-DAP^Dipp^)_2_(μ_4_-κ^1^:κ^1^:κ^1^:η^2^-DAP^Dipp^)_2_ (**11**), was successfully synthesized via KC_8_ reduction of **9**. These results highlight the essential role of ligand sterics in stabilizing high-nuclearity Ge clusters. In contrast, attempts to isolate a smaller DAP^Mes^-substituted analogue, K_2_Ge_6_(μ_3_-κ^1^:κ^1^:κ^1^-DAP^Mes^)_2_(μ_4_-κ^1^:κ^1^:κ^1^:η^2^-DAP^Mes^)_2_, were unsuccessful; K_2_(DAP^Mes^) was the only identifiable product from the reduction of **4** and **7**.

The molecular structures of compounds **10** and **11** were confirmed by single-crystal X-ray diffraction (Figures S9 and 6a). In contrast to previously reported Ge_6_ clusters that adopt chairlike, linear, trigonal prismatic, or octahedral geometries,^65^ both **10** and **11** crystallize with *C*_2h_ symmetry and feature a snake-like Ge_6_ core stabilized by four DAP ligands. Structurally, these two clusters can be described as dimers of two KGe_3_ fragments connected by a Ge3–Ge3′ bond, with one DAP ligand in each fragment displaced outward. Consequently, each Ge atom of the central Ge3–Ge3′ unit is solely supported by an amido ligand. Within each fragment, the remaining two Ge atoms are each ligated by one amido and one pyridyl donor, while the K^+^ ion is coordinated by an amido group together with a THF molecule and a phenyl ring from an adjacent Dipp substituent. This arrangement is structurally analogous to the digermylene complex K_2_Ge_2_(DAP^Dipp^)_2_ (**12**)^37^ and suggests that all Ge atoms adopt a formal oxidation state of +1. Accordingly, the Ge1–Ge2 and Ge1′–Ge2′ interactions can be described as conventional 2c–2e covalent bonds, whereas the Ge2→Ge3 and Ge2′→Ge3′ interactions are best represented as donor–acceptor (dative) bonds. In addition, the Ge1 and Ge1′ atoms donate their lone pairs to the neighboring K^+^ ions.

**Figure 6:**
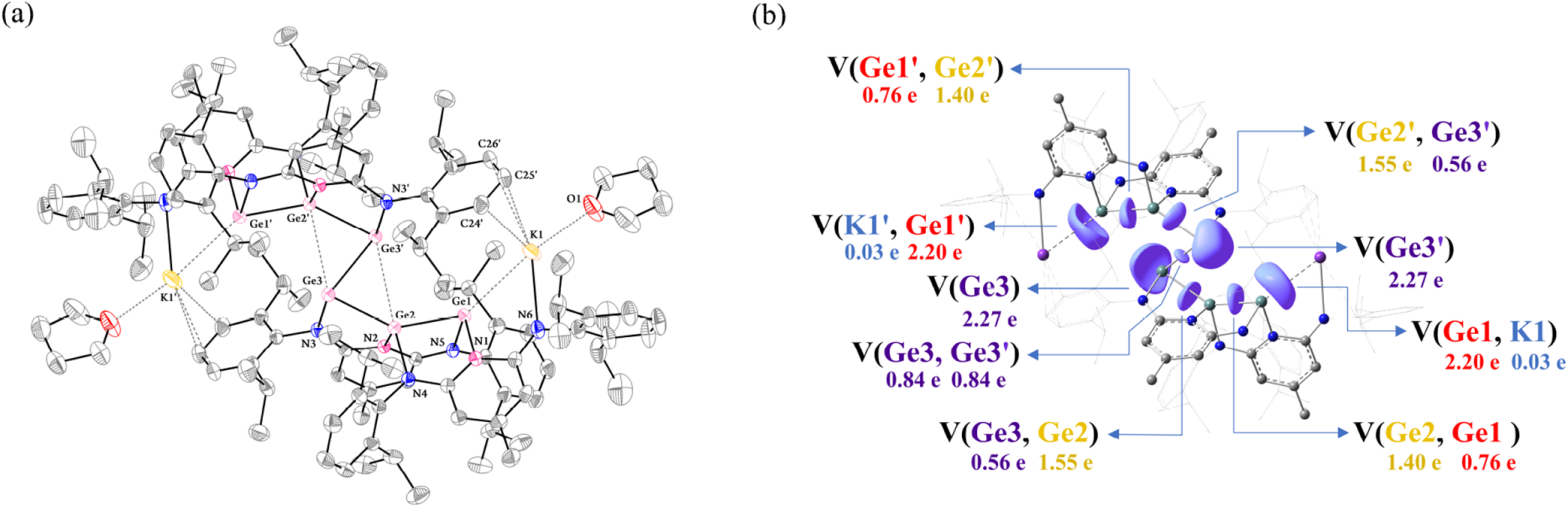
(a) The solid-state molecular structure of **11** with thermal ellipsoids at 30% probability. The hydrogen atoms have been omitted for clarity. Selected bond lengths (Å) and angles (°): Ge1–Ge2, 2.4446(4); Ge2–Ge3, 2.4734(4); Ge3–Ge3′, 2.7634(5); Ge1···K1, 3.1870(8); K1···C24′, 3.233(4); K1···C25′, 3.063(4); K1···C26′, 3.388(4); K1–O1, 2.563(3); Ge2–N2, 1.9671(19); Ge2–N4, 1.960(2); Ge1–N1, 1.993(2); Ge1–N5, 2.016(2); Ge3–N3, 2.059(2); K1–N6, 2.683(3); Ge2–Ge3–Ge3′, 77.609(12); Ge1–Ge2–Ge3, 141.969(14); Ge2–Ge1–K1, 136.68(2); Ge1–K1–O1, 155.82(9); N3–Ge3–Ge3′, 98.34­(6); N1–Ge1–N5, 98.27(8); N2–Ge2–N4, 99.46(9). (b) ELF plots of **11m**. The ELF function of η­(**r**) = 0.7 is shown around Ge.

In both compounds, the unsupported Ge3–Ge3′ bonds adopt a *trans*-bent geometry, with N3–Ge3–Ge3′ and Ge2–Ge3–Ge3′ bond angles of 91.95(6)° and 74.48(1)° in **10**, and 98.34(6)° and 77.61(1)° in **11**, respectively. This structural arrangement is consistent with the presence of a stereoactive lone pair on both Ge3 and Ge3′ centers, which adopt distorted trigonal pyramidal configurations. The Ge3–Ge3′ bond lengths measure 2.7216(6) Å (**10**) and 2.7634(5) Å (**11**), values notably longer than those observed in known Ge^I^–Ge^I^ dimers (2.506–2.709 Å),^39–50^ reflecting increased steric congestion in the bulkier DAP^Dipp^ system.^70^ The Ge_3_ unit within each fragment adopts significantly bent conformation, as indicated by Ge1–Ge2–Ge3 bond angles of 144.33(2)° in **10** and 141.97(1)° in **11**. The decreased N2–Ge2–N4 bond angles of 98.38(9)° in **10** and 99.46(9)° in **11** differ from that in compounds **4** and **5**. The Ge1–Ge2 bond lengths are 2.4398(4) Å in **10** and 2.4446(4) Å in **11**, comparable to values observed in related species such as (GeCl)_2_Ge_2_(DAP^Dipp^)_2_ (**13**, 2.4071(6) Å) and the cyclic [Ge_4_(DAP^Dipp^)_2_]_2_ (**14**, 2.4383(9) Å).^37^ The Ge2–Ge3 bond lengths (2.4421(4) Å (**10**), 2.4734(4) Å (**11**)) are comparable to the corresponding Ge1–Ge2 bonds. Notably, the Ge2–Ge3–Ge3′–Ge2′ core in **10** and **11** adopts an intriguing Z-shaped geometry, in which the lone pairs of Ge3 and Ge3′ are oriented toward Ge2′ and Ge2, respectively. The two crystallographically identical Ge2···Ge3′ and Ge2′···Ge3 separations measure 3.1328(4) Å in **10** and 3.2895(3) Å in **11**, significantly shorter than the sum of the van der Waals radii of two Ge atoms (4.22 Å).^64^ This suggests a notable through-space interaction. Consistent with the intermolecular Ge···Cl contacts observed in **6**, Ge2 and Ge2′ also exhibit Lewis acidic character. In both compounds, the Ge2–N2 and Ge2–N4 bonds fall within the narrow range (ca. 1.95–1.97 Å), similar to the Ge–N_py_ bonds in **4** and **5** (ca. 1.97 Å), indicating that the bonding environment at Ge2 resembles that at the Ge centers in **4** and **5**. Additionally, the Ge···K^+^ distances in **10** and **11** (ca. 3.17–3.19 Å) are significantly shorter than those observed in **12** for the Ge···K^+^ separations (3.49–3.52 Å),^37^ suggesting stronger ion-pair interactions. The ^1^H NMR spectra of **10** and **11** are in good agreement with their solid-state structures. All four DAP ligands are magnetically equivalent, while the Dipp substituents and the pyridyl meta-protons exhibit inequivalent signals, consistent with the anisotropic environments of the ligands around the asymmetric Ge_6_ core.

To gain further insight into the bonding schemes within the Ge_6_ core of compounds **10** and **11**, DFT calculations were performed on the THF-free model compound K_2_Ge_6_(μ_3_-κ^1^:κ^1^:κ^1^-DAP^Dipp^)_2_(μ_4_-κ^1^:κ^1^:κ^1^:η^2^-DAP^Dipp^)_2_ (**11m**). The optimized geometry of **11m** shows an excellent agreement with the experimental structure (Table S6). For example, the calculated Ge1–Ge2 and Ge2–Ge3 bond lengths are 2.4548 Å and 2.4917 Å, respectively, while the unsupported Ge3–Ge3′ bond is slightly underestimated at 2.6716 Å. NBO analysis (Figures S62 and S63) revealed that the Ge3–Ge3′ bond is characterized as a σ-type interaction dominated by p-orbital overlap (Ge: sp^10.32^), with each Ge3 atom also retaining a lone pair of high s-character (sp^0.24^). Compared with **4** and **5**, Ge2 in **11m** is involved in two polarized, asymmetric σ-interactions with the adjacent Ge atoms, as reflected in the NBO compositions: Ge2–Ge3 [64% Ge2 (sp^1.09^), 36% Ge3 (sp^14.06^)], and Ge2–Ge1 [60% Ge2 (sp^1.38^), 40% Ge1 (sp^7.14^)]. Two Ge–N interactions are also present involving the pyridyl and amido N atoms. ELF analysis (Figures 6b and S69) further supports this bonding description. A well-localized lone pair is present on Ge3 [V­(Ge3) = 2.27 e]. Two disynaptic basins, V­(Ge1,Ge2) and V­(Ge2,Ge3), are also observed and display unequal electron sharing, with larger populations associated with Ge2 (1.40 and 1.55 e) than with Ge1 and Ge3 (0.76 and 0.56 e). These values are comparable to those found in **5**. Notably, the lone pair on Ge1 is oriented toward the adjacent K^+^ ion, with a basin population of 2.20 e, consistent with a Ge→K donor–acceptor interaction.

For comparative purposes, we synthesized an analogue of compound **11**, in which the unsupported Ge3–Ge3′ bond is replaced by a Sn–Sn unit to probe the impact of this substitution on hybridization and bonding within the Ge_4_Sn_2_ cluster. K/Hg reduction of (SnCl)_2_Ge_2_(μ_3_-κ^1^:κ^1^:κ^1^-DAP^Dipp^)_2_ (**15**; see the Supporting Information for details) furnished the heterobimetallic hexanuclear Ge_4_Sn_2_ cluster, [K­(Et_2_O)]_2_Ge_4_Sn_2_(μ_3_-κ^1^:κ^1^:κ^1^-DAP^Dipp^)_2_(μ_4_-κ^1^:κ^1^:κ^1^:η^2^-DAP^Dipp^)_2_ (**16**), which is analogous to compounds **10** and **11** (Scheme 2). The solid-state structures of **15** and **16** are shown in Figures S11 and 7, respectively. Compound **15** adopts a conformation similar to that of **13**, featuring a central digermylene unit bridged by two diamidopyridine ligands in an eclipsed arrangement; each outer Sn atom displays a distorted trigonal-pyramidal geometry consistent with a stereochemically active lone pair. In **15**, the Sn-bound Cl atoms are oriented away from the phenyl groups of the supporting ligands, likely due to repulsive interactions between the Cl lone pairs and the phenyl rings. Notably, the Ge–Ge bond length in **15** (2.3735(6) Å) is shorter than that in **13** (2.4071(6) Å),^37^ a difference attributed to the increased acidity of the Sn atom, which reduces repulsion between lone pairs on the Ge centers. Moreover, the two equivalent Sn–Ge bond lengths in **15** (ca. 2.68 Å) fall within the reported range for Sn–Ge single bonds (2.599(3)-2.7847(7) Å).^12,41,71–75^

**Figure 7:**
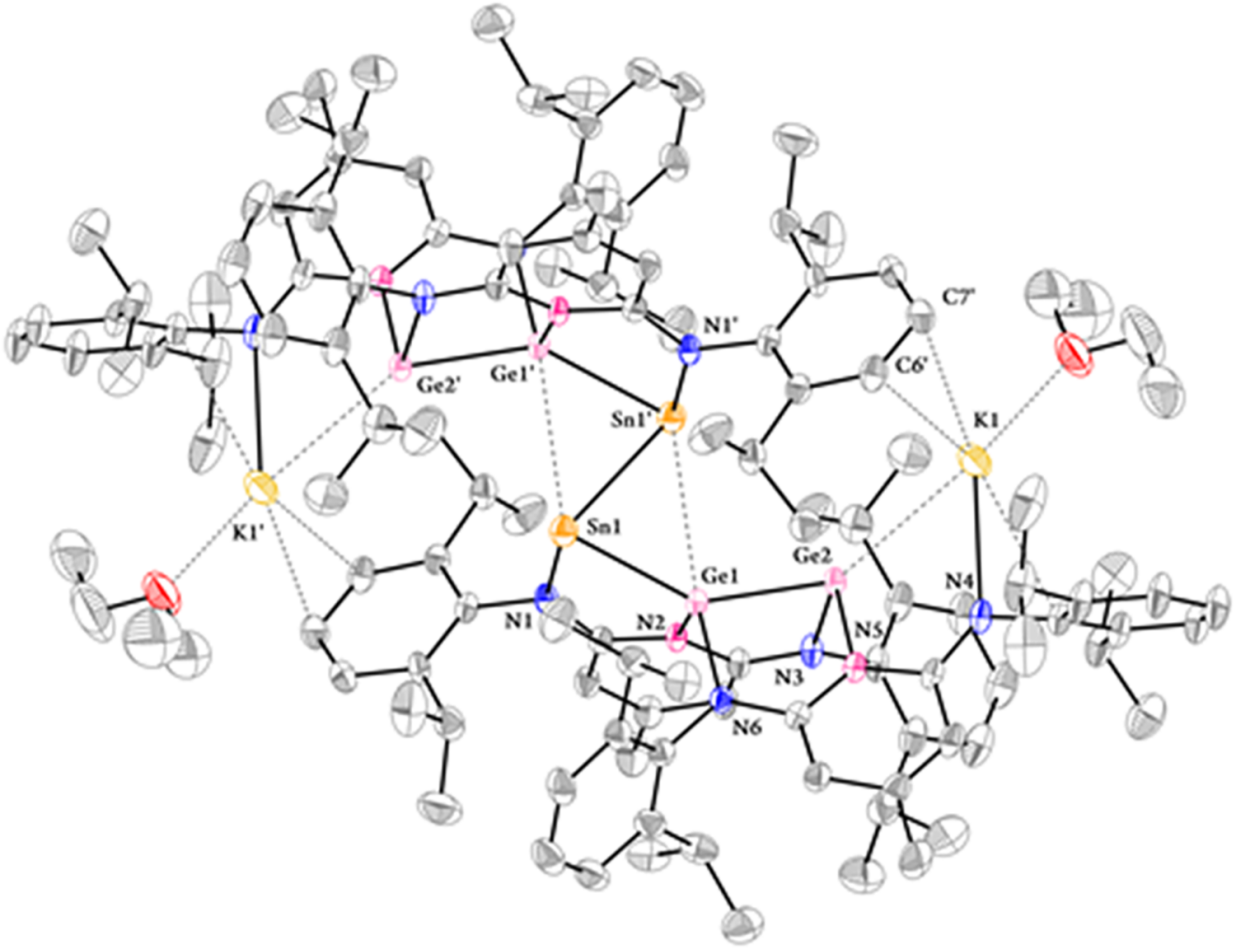
Solid-state molecular structure of **16** with thermal ellipsoids at 30% probability where the hydrogen atoms were omitted for the sake of clarity. Selected bond lengths (Å) and angles (°): Sn1–Sn1′, 2.9718(9); Sn1–Ge1, 2.5865(7); Ge1–Ge2, 2.4473(8); Ge2···K1, 3.2605(18); Sn1···Ge1′, 3.3944(8); Sn1–N1, 2.195(5); 2.021(5); K1···C7′, 3.194(8); K1···C6′, 3.285(7), K1–O1, 2.669(6); K1–N4, 2.729(6); K1–O1 2.669(6); Ge1–N2, 1.984(4); Ge1–N6, 1.953(5); Ge2–N3, 1.996(5); Ge2–N5, Sn1′–Sn1–Ge1, 74.93(2); Sn1–Ge1–Ge2, 142.21(3); Ge1–Ge2–K1, 137.51(4); Sn1′–Sn1–N1, 93.71­(13); Ge1–Sn1–N1, 80.38(12); N2–Ge1–N6, 103.7(2); N3–Ge2–N5, 97.0(2).

The structure of **16** closely resembles that of compounds **10** and **11**, likewise featuring a snake-like Ge_4_Sn_2_ core, that can be described as a dimer of two Ge_2_Sn fragments linked by an Sn1–Sn1′ bond. Each tin atom in the central Sn1–Sn1′ unit is solely coordinated by an amido ligand, while the coordination environment of the two Ge_2_K fragments is identical to that observed in **10** and **11**. Accordingly, the bonding interactions in the Ge_4_Sn_2_ core parallel those in the Ge_6_ cores of **10** and **11**, with both Sn and the four Ge atoms adopting a formal oxidation of +1. The conformation of the two Ge_2_Sn fragments in **16** is also similar to that of the Ge_3_ fragment in **10** and **11**, as evidenced by the Sn1–Ge1–Ge2 bond angle of 142.21(3)°, which is comparable to the Ge1–Ge2–Ge3 angles in **10** and **11**. Like the unsupported Ge3–Ge3′ bond in **10** and **11**, the unsupported Sn1–Sn1′ bond in **16** also adopts a *trans-bent* configuration, with N1–Sn1–Sn1′ and Ge1–Sn1–Sn1′ angles of 93.71(13)° and 74.93(2)°, respectively. Each Sn atom, besides its sterically active lone pair, is bonded to another Sn atom, an amido nitrogen donor, and a Ge atom, resulting in a distorted trigonal-pyramidal geometry. The Sn1–Sn1′ bond length in **16** is 2.9718(7) Å, which is comparable to those observed in distannynes (RSn–SnR, ca. 2.89–3.06 Å).^49,76–85^ Furthermore, the Ge1→Sn1 and Ge1′→Sn1′ bonds measure 2.5865(7) Å, values slightly shorter than the lower limit reported for Sn–Ge single bonds (2.599(3)-2.7847(7) Å),^12,41,71–75^ while the Ge2–Ge1 and Ge2′–Ge1′ bond lengths (2.4473(8) Å) are similar to those in **10** and **11** (ca. 2.44 Å). Similar to the Z-shaped conformation of the central Ge_4_ core in **10** and **11**, the Ge1–Sn1–Sn1–Ge1′ unit also adopts a Z-shaped geometry, where the lone pairs on Sn1 and Sn1′ are oriented toward Ge1′ and Ge1, respectively. The two equivalent Sn···Ge distances are 3.3944(8) Å, shorter than the sum the van der Waals radii of Ge and Sn atoms (4.28 Å), indicating the Lewis acidic character of Ge1 and Ge1′. Consistent with these structural assignments, the ^119^Sn NMR spectrum of **15** in C_6_D_6_ shows a singlet at −245.3 ppm, whereas **16** exhibits a more downfield singlet at −94.7 ppm, reflecting the lower oxidation state of the Sn atoms in **16**.^86,87^

## Conclusions

In summary, we have developed a novel synthetic strategy that leverages zerovalent monatomic germanium chemistry to stabilize low-valent group 14 species, leading to the first examples of trigermanium compounds ((μ-Ge)­(κ^2^-**N**_**2**_ **Ge**_**2**_^**Ar**^)) (**4** and **5**) with the central Ge^0^ atom supported by a unique four-electron cyclic dipyridyl-digermylene donor ligand **N**_**2**_ **Ge**_**2**_^**Ar**^. Detailed spectroscopic, crystallographic, and DFT studies reveal that **4** and **5** are generated via isomerization of bis­(DSGe^Ar^) (**2** and **3**)-supported germylone intermediates. The central Ge^0^ atom adopts an unprecedented sp-hybridized, seesaw geometry. In this arrangement, the central Ge^0^ atom is supported by two nitrogen donors and donates its electron pairs to the outer Ge^II^ centers, each of which retains a localized lone pair.

This unique electronic structure of (μ-Ge)­(κ^2^-**N**_**2**_ **Ge**_**2**_^**Dep**^) is further validated by the formation of a donor–acceptor adduct (**6**) and by its reactivity toward KC_8_ reduction, which affords a novel hexanuclear homounivalent germanium cluster (**10**) exhibiting a snake-like Ge_6_ core. The inability to generate an analogous cluster from less sterically demanding precursors, together with the successful isolation of a bulkier analogue (**11**), underscores the critical role of ligand congestion in stabilizing these multinuclear assemblies.

Furthermore, by extending this approach to heteronuclear systems, we have synthesized a hexanuclear Ge_4_Sn_2_ cluster (**16**) from a DAP^Dipp^-supported Ge_2_Sn_2_Cl_2_ precursor. The Ge_4_Sn_2_ core in **16** mirrors the snake-like architecture observed in the Ge_6_ clusters in **10** and **11**, featuring three distinct E–E bond types (Sn–Sn, Ge→Sn, and Ge–Ge). Overall, these findings not only expand the structural diversity of low-valent group 14 compounds but also establish a new paradigm for constructing multinuclear tetrel clusters. The precise modulation of bonding interactions through steric and electronic tuning in these systems paves the way for future developments in main-group chemistry and materials science. Ongoing investigations in our laboratory will further explore the reactivity and potential applications of these unprecedented clusters.

## Experimental Section

### General Considerations

All manipulations were carried out using standard Schlenk and glovebox techniques under an atmosphere of high-purity nitrogen. Diethyl ether (Et_2_O) and tetrahydrofuran (THF) were distilled under nitrogen from purple sodium benzophenone ketyl. *n*-Pentane, *n*-hexane and toluene were passed through columns of solvent purification systems (Vigor VAPA-5) to remove oxygen and moisture. Distilled solvents were transferred under vacuum into vacuum-tight glass vessels before being transferred into a glovebox. C_6_D_6_ and *d*_8_-THF were purchased in ampules from Sigma-Aldrich and stored over 4 Å molecular sieves in Schlenk tubes. Four Å molecular sieves and *Celite* were dried in a vacuum at 200 °C for 3 days. All other commercially available chemicals were used without further purification. Elemental analyses were performed with the Elementar vario EL CUBE CHN-OS Rapid. The ^1^H, ^13^C­{^1^H} and ^119^Sn­{^1^H} NMR spectra were recorded with Varian Unity INOVA-500 MHz, Varian Unity INOVA-400 MHz and Bruker Avance −500 MHz spectrometer and referenced internally the residue of the solvent resonances (C_6_D_6_: ^1^H: 7.16 ppm, ^13^C­{^1^H}: 128.06 ppm; *d*_8_-THF: ^1^H: 1.72 and 3.58 ppm, ^13^C­{^1^H}: 25.31 and 67.21 ppm).^88^ ^119^Sn­{^1^H} NMR spectra were referenced externally with respect to SnMe_4_. The dilithiated 2,6-diamidopyridines Li_2_[(DAP^Ar^)] (DAP^Ar^ = 2,6-(ArN)_2_-4-CH_3_C_5_H_2_N); Ar = Dipp, Dep, Mes; Dipp = 2,6-^*i*^Pr_2_C_6_H_3_, Dep = 2,6-Et_2_C_6_H_3_, Mes = 2,4,6-Me_3_C_6_H_2_,^37^ KC_8_,^89^ and K/Hg^90^ were synthesized following the documented methods.

### Synthesis and Characterization

#### Ge_2_(μ-κ^1^:κ^2^-DAP^Dipp^)_2_ (**1**)

**1** was synthesized according to modified literature procedure.^37^ A 20 mL of vial was charged with Li_2_[(DAP^Dipp^)] (0.0922g, 0.1741 mmol) and GeCl_2_·dioxane (0.0433 g, 0.1870 mmol), and 2 mL of THF was added as solvent. The reaction mixture was allowed to stir for 1 h at room temperature. At this point, the yellow solution was obtained, and the solvent was removed by *vacuo*. The residue was extracted with 2 mL of *n*-hexane for three times and filtered through a pad of *Celite* to remove insoluble material. The yellow filtrate was concentrated under vacuum to give a yellow solid (0.0742 g, 0.0721 mmol, 82.8%). ^1^H NMR (500 MHz, C_6_D_6_, 298 K) δ 7.26–7.03 (m, 12H, 2,6-^*i*^Pr_2_C_6_ *H*_3_), 5.18 (s, 2H, 4-CH_3_C_5_ *H*_2_N), 4.96 (s, 2H, 4-CH_3_C_5_ *H*_2_N), 3.64 (septet, 4H, *H*CMe_2_), 3.50 (septet, 2H, *H*CMe_2_), 2.34 (septet, 2H, *H*CMe_2_), 1.39 (s, 6H, 4–C*H*_3_C_5_H_2_N), 1.37 (d, 6H, CH*Me*_2_), 1.36 (d, 6H, CH*Me*_2_), 1.33 (d, 6H, CH*Me*_2_), 1.17 (d, 6H, CH*Me*_2_), 1.14 (d, 6H, CH*Me*_2_), 1.06 (d, 6H, CH*Me*_2_), 0.80 (d, 6H, CH*Me*_2_), 0.75 (d, 6H, CH*Me*_2_). ^13^C­{^1^H} NMR (126 MHz, C_6_D_6_, 298 K) δ 169.0, 158.4, 152.1, 148.7, 147.8, 147.3, 145.7, 139.9, 139.2, 127.6, 126.9, 125.0, 125.0, 124.6, 123.6, 99.2 (*C*H_2_, *meta*-4-Me-pyridine), 94.9 (*C*H_2_, *meta*-4-Me-pyridine), 30.2, 29.9, 28.7, 27.9, 27.6, 26.9, 25.9, 25.2, 24.9, 24.8, 24.3, 24.2, 21.5 (*C*H_3_, 4-Me-pyridine). Anal. Calcd for C_60_H_78_N_6_Ge_2_: C, 70.06; H, 7.64; N, 8.17. Found: C, 69.57; H, 7.71; N, 7.91.

#### Ge_2_(μ-κ^1^:κ^2^-DAP^Mes^)_2_ (**2**)

The mixture of Li_2_[(DAP^Mes^)] (0.2070g, 0.4646 mmol) and GeCl_2_·dioxane (0.1145 g, 0.4946 mmol) was taken into a 20 mL of vial and 2 mL of THF were added at room temperature. The reaction mixture was allowed to stir for 1 h. All volatiles were removed by *vacuo*. The residue was extracted with 2 mL of toluene for three times, and the insoluble material was filtered off through a pad of *Celite*. The yellow filtrate was concentrated under vacuum to give a yellow solid (0.1079 g, 0.1254 mmol, 53.9%). X-ray quality crystals of **2** were obtained from evaporation of toluene at room temperature. ^1^H NMR (500 MHz, C_6_D_6_, 298 K) δ 6.89 (s, 2H, 2,4,6-Me_3_C_6_ *H*_2_), 6.73 (s, 4H, 2,4,6-Me_3_C_6_ *H*_2_), 6.66 (s, 2H, 2,4,6-Me_3_C_6_ *H*_2_), 5.37 (s, 2H, 4-CH_3_C_5_ *H*_2_N), 5.02 (s, 2H, 4-CH_3_C_5_ *H*_2_N), 2.56 (s, 6H, 2,4,6-*Me*_3_C_6_H_2_), 2.38 (s, 6H, 2,4,6-*Me*_3_C_6_H_2_), 2.19 (s, 6H, 2,4,6-*Me*_3_C_6_H_2_), 2.16 (s, 6H, 2,4,6-*Me*_3_C_6_H_2_), 2.06 (s, 6H, 2,4,6-*Me*_3_C_6_H_2_), 1.60 (s, 6H, 2,4,6-*Me*_3_C_6_H_2_), 1.49 (s, 6H, 4–C*H*_3_C_5_H_2_N). ^13^C­{^1^H} NMR (126 MHz, C_6_D_6_, 298 K) δ 166.9, 157.3, 152.5, 141.4, 138.5, 137.2, 137.0, 134.9, 134.6, 134.4, 130.5, 129.7, 129.4, 128.5, 97.8 (*C*H_2_, *meta*-4-Me-pyridine), 93.0 (*C*H_2_, *meta*-4-Me-pyridine), 21.8 (*C*H_3_, 4-Me-pyridine), 20.9 (*C*H_3_), 20.9 (*C*H_3_,), 20.7 (*C*H_3_), 20.3 (*C*H_3_), 19.4 (*C*H_3_), 17.2 (*C*H_3_). Anal. Calcd for C_48_H_54_N_6_Ge_2_: C, 67.02; H, 6.33; N, 9.77. Found: C, 67.70; H, 6.80; N, 9.46.

#### Ge_2_(μ-κ^1^:κ^2^-DAP^Dep^)_2_ (**3**)

Addition of 2 mL of THF into the reaction mixture of Li_2_[(DAP^Dep^)] (0.1010 g, 0.2133 mmol) and GeCl_2_·dioxane (0.0537 g, 0.2320 mmol) at room temperature resulting in the formation of yellow solution immediately. After stirring for 1 h, volatiles were removed under vacuum, and then 6 mL of *n*-hexane was added into the residue. The yellow suspension was filtered through a pad of *Celite*. The filtrate was concentrated by *vacuo* to give a yellow solid (0.0643 g, 0.0701 mmol, 65.7%). X-ray quality crystals of **3** were obtained from recrystallization in *n*-hexane at −30 °C or evaporation of toluene at room temperature. ^1^H NMR (500 MHz, C_6_D_6_, 298 K) δ 7.24–6.95 (m, 12H, 2,6-Et_2_C_6_ *H*_3_), 5.28 (s, 4H, 4-CH_3_C_5_ *H*_2_N), 4.95 (s, 4H, 4-CH_3_C_5_ *H*_2_N), 3.10 and 3.01 (m, 8H, 2,6-(C*H*_2_CH_3_)_2_C_6_H_3_), 2.84 (m, 2H, 2,6-(C*H*_2_CH_3_)_2_C_6_H_3_), 2.61 (m, 4H, 2,6-(C*H*_2_CH_3_)_2_C_6_H_3_), 2.05 (m, 2H, 2,6-(C*H*_2_CH_3_)_2_C_6_H_3_), 1.46 (m, 2H, 2,6-(C*H*_2_CH_3_)_2_C_6_H_3_), 1.40 (s, 6H, 4–C*H*_3_C_5_H_2_N), 1.27 (t, 6H, 2,6-(CH_2_C*H*_3_)_2_C_6_H_3_), 1.21 (t, 6H, 2,6-(CH_2_C*H*_3_)_2_C_6_H_3_), 1.02 (t, 6H, 2,6-(CH_2_C*H*_3_)_2_C_6_H_3_), 0.85 (t, 6H, 2,6-(CH_2_C*H*_3_)_2_C_6_H_3_). ^13^C­{^1^H} NMR (126 MHz, C_6_D_6_, 298 K) δ 167.8, 157.7, 152.5, 143.3, 142.8, 142.3, 141.5, 141.1, 140.1, 127.2, 126.8, 126.4, 126.3, 125.7, 98.3 (*C*H_2_, *meta*-4-Me-pyridine), 93.4 (*C*H_2_, *meta*-4-Me-pyridine), 26.4, 26.1, 24.2, 22.6, 21.7 (*C*H_3_, 4-Me-pyridine), 17.1, 16.2, 15.1, 13.9. Anal. Calcd for C_52_H_62_N_6_Ge_2_: C, 68.16; H, 6.82; N, 9.17. Found: C, 68.579; H, 7.142; N, 9.061.

#### (μ-Ge)­(κ^2^-N_2_Ge_2_^Mes^) (**4**)

The mixture of **2** (0.1675 g, 0.1947 mmol) and KC_8_ (0.0543 g, 0.4017 mmol) was taken into a 20 mL of vial and 4 mL of THF were added at room temperature. The reaction mixture was allowed to stir for 1 h to give a yellow green suspension. All volatiles were removed by *vacuo*. The residue was extracted with 2 mL of *n*-hexane for three times, and the insoluble material was filtered off through a pad of *Celite*. The yellow filtrate was concentrated under vacuum to give a yellow solid (0.0515 g, 0.0552 mmol, 28.4%). **Method 2**: In a glovebox, a 20 mL vial was charged with **2** (0.0674 g, 0.0783 mmol), Ge powder (0. 114 g, 1.566 mmol), 1 mL of Et_2_O and a magnetic stir bar. The reaction mixture was allowed to stir for 7 days. At this point, the suspension was filtered through a pad of Celite to remove insoluble material. The filtrate was evaporated by *vacuo* and the residue was extracted into 2 mL of *n*-pentane for three times. A yellow solid of 4 was isolated in 22.8% yield (0.0167 g, 0.0179 mmol) after all volatiles were removed by *vacuo*. **Method 3**: A 20 mL of vial was charged with **7** (0.0743 g, 0.0648 mmol) and KC_8_ (0.0424 g, 0.3136 mmol), and 2 mL of Et_2_O was added as solvent. The reaction mixture was allowed to stir for 4 h at room temperature. At this point, the brown suspension was obtained, and the solvent was removed by *vacuo*. The residue was extracted with 2 mL of *n*-hexane for three times and filtered through a pad of *Celite* to remove the black insoluble material. The brown filtrate was concentrated under vacuum to give a brown solid (0.037 g, 0.0397 mmol, 61.3%). X-ray quality crystals of **4** were obtained from evaporation of *n*-hexane at room temperature. ^1^H NMR (500 MHz, C_6_D_6_, 298 K) δ 6.82 (s, 4H, 2,4,6-Me_3_C_6_ *H*_2_-N), 6.80 (s, 4H, 2,4,6-Me_3_C_6_ *H*_2_-N), 5.36 (s, 4H, 4-CH_3_C_5_ *H*_2_N), 2.28 (s, 12H, 2,4,6-*Me*_3_C_6_H_2_–N), 2.14 (s, 12H, 2,4,6-*Me*_3_C_6_H_2_–N), 2.00 (s, 12H, 2,4,6-*Me*_3_C_6_H_2_–N), 1.41 (s, 6H, 4–C*H*_3_C_5_H_2_N). ^13^C­{^1^H} NMR (126 MHz, C_6_D_6_, 298 K) δ 160.3, 154.4, 143.0, 136.8, 135.3, 135.2, 130.4, 130.2, 97.8 (*C*H_2_, *meta*-4-Me-pyridine), 21.3 (*C*H_3_, *meta*-4-Me-pyridine), 21.0 (*C*H_3_), 19.5 (*C*H_3_), 18.4 (*C*H_3_). Anal. Calcd for C_48_H_54_N_6_Ge_3_: C, 61.80; H, 5.83; N, 9.01. Found: C, 61.45; H, 6.00; N, 8.66.

#### (μ-Ge)­(κ^2^-N_2_Ge_2_^Dep^) (**5**)

A 20 mL of vial was charged with **3** (0.0504 g, 0.0550 mmol) and KC_8_ (0.0092 g, 0.0681 mmol), and the mixture of *n*-hexane and Et_2_O in 2:1 ratio was added as solvent. The reaction mixture was allowed to stir for 4 h at room temperature. At this point, the reddish-brown suspension was obtained, and the solvent was removed by *vacuo*. The residue was extracted with 2 mL of Et_2_O for three times and filtered through a pad of *Celite* to remove the black insoluble material. The brown filtrate was concentrated under vacuum to give a brown solid (0.0282 g, 0.0285 mmol, 51.8%). **Method 2**: In a glovebox, a 20 mL vial was charged with **3** (0.0504 g, 0.0550 mmol), Ge powder (0.0800 g, 1.10 mmol), 1.5 mL of Et_2_O and a magnetic stir bar. The reaction mixture was allowed to stir for 7 days. At this point, the suspension was filtered through a pad of *Celite* to remove insoluble material. The filtrate was evaporated by *vacuo* and the residue was extracted into 2 mL of *n*-pentane for three times. A brown solid of **5** was isolated in 31.2% yield (0.0170 g, 0.0172 mmol) after all volatiles were removed by *vacuo*. **Method 3**: A 20 mL of vial was charged with **8** (0.3070 g, 0.2551 mmol) and KC_8_ (0.1930 g, 1.4277 mmol), and 8 mL of Et_2_O was added as solvent. The reaction mixture was allowed to stir for 1 h at room temperature. At this point, the brown suspension was obtained, and the solvent was removed by *vacuo*. The residue was extracted with 2 mL of Et_2_O for three times and filtered through a pad of *Celite* to remove the black insoluble material. The brown filtrate was concentrated under vacuum to give a brown solid (0.2140 g, 0.2164 mmol, 84.8%). X-ray quality crystals of **5** were obtained from evaporation of *n*-hexane at room temperature. ^1^H NMR (500 MHz, C_6_D_6_, 298 K) δ 7.15–7.09 (m, 12H, 2,6-Et_2_C_6_ *H*_3_), 5.24 (s, 4H, 4-CH_3_C_5_ *H*_2_N), 2.78 and 2.70 (m, 8H, 2,6-(C*H*_2_CH_3_)_2_C_6_H_3_), 2.40 (m, 8H, 2,6-(C*H*_2_CH_3_)_2_C_6_H_3_), 1.34 (s, 6H, 4–C*H*_3_C_5_H_2_N), 1.13 (t, 12H, 2,6-(CH_2_C*H*_3_)_2_C_6_H_3_), 1.01­(t, 12H, 2,6-(CH_2_C*H*_3_)_2_C_6_H_3_). ^13^C­{^1^H} NMR (126 MHz, C_6_D_6_, 298 K) δ 160.8, 154.0, 144.3, 142.1, 141.4, 127.5, 126.6, 126.3, 98.7 (*C*H_2_, *meta*-4-Me-pyridine), 25.8, 23.5, 21.0 (*C*H_3_, 4-Me-pyridine), 15.5, 14.0. Anal. Calcd for C_52_H_62_NGe_3_: C, 63.15; H, 6.32; N, 8.50. Found: C, 62.59; H, 6.39; N, 8.30.

#### [(Cl_3_In)←(μ-Ge)­(κ^2^-N_2_Ge_2_^Dep^)]_2_ (**6**)

Addition of 2 mL of THF into the reaction mixture of **5** (0.1281 g, 0.1295 mmol) and InCl_3_ (0.0626 g, 0.2830 mmol) at room temperature resulting in the formation of orange solution immediately. After stirring for 1 h, volatiles were removed under vacuum, and 6 mL of the solution of *n*-hexane and toluene in 1:1 ratio was added into the residue. The yellow suspension place in the freezer at −30 °C and was filtered through a pad of *Celite*. The filtrate was concentrated by *vacuo.* Yellow crystals of **6** were obtained from recrystallization in Et_2_O at −30 °C. The crystals were washed with cold Et_2_O to afford **6** as a yellow solid after dried by *vacuo* (0.064 g, 0.0529 mmol, 40.8%). X-ray quality crystals of **6** were obtained from evaporation of C_6_D_6_ at room temperature or recrystallization in Et_2_O at −30 °C. ^1^H NMR (400 MHz, C_6_D_6_, 298 K) δ 7.10 (m, 12H, 2,6-^*i*^Pr_2_C_6_H_3_), 5.36 (s, 4H, 4-CH_3_C_5_ *H*_2_N), 2.81 and 2.26 (m, 16H, 2,6-(C*H*_2_CH_3_)_2_C_6_H_3_), 1.25 (t, 12H, 2,6-(CH_2_C*H*_3_)_2_C_6_H_3_), 1.17 (s, 6H, 4–C*H*_3_C_5_H_2_N), 0.96 (t, 12H, 2,6-(CH_2_C*H*_3_)_2_C_6_H_3_). ^13^C­{^1^H} NMR (101 MHz, C_6_D_6_, 298 K) δ 159.9, 155.6, 142.1, 141.8, 126.4, 101.1 (*C*H_2_, *meta*-4-Me-pyridine), 26.4, 23.6, 20.9 (*C*H_3_, 4-Me-pyridine), 15.4, 13.7. Anal. Calcd for C_52_H_62_N_6_InCl_3_Ge_3_: C, 51.61; H, 5.16; N, 6.94. Found: C, 51.25; H, 5.09; N, 6.71.

#### (GeCl)_4_(μ-κ^1^:κ^1^-DAP^Mes^)_2_ (**7**)

A 20 mL of vial was charged with **2** (0.1004 g, 0.1167 mmol) and GeCl_2_·dioxane (0.0566 g, 0.2445 mmol), and 4 mL of THF was added as solvent. The reaction mixture was allowed to stir for 2 h at room temperature. At this point, the orange solution was obtained, and the solvent was removed by *vacuo*. The crude material was washed with the mixture of *n*-hexane and Et_2_O in 1:1 ratio to afford **7** as an orange solid after dried by *vacuo* (0.0599 g, 0.0522 mmol, 44.7%). **Method 2**: In a glovebox, a 20 mL of vial was charged with a solution of **4** (0.0629 g, 0.0674 mmol) in 3 mL of THF. To this solution was slowly added 0.51 mL of 0.12 M GeCl_4_ (0.0612 mmol) in *n*-hexane. The reaction mixture was allowed to stir overnight at room temperature. At this point, all volatiles were removed by vacuo and the residue was extracted into 2 mL of *n*-hexane for three times. An orange solid of **7** was obtained in 24.5% yield (0.0188 g, 0.0165 mmol) after all volatiles were removed by vacuo. ^1^H NMR (500 MHz, C_6_D_6_, 298 K) δ 6.82 (s, 8H, 2,4,6-Me_3_C_6_ *H*_2_-N), 5.20 (s, 4H, 4-CH_3_C_5_ *H*_2_N), 2.50 (s, 24H, 2,4,6-*Me*_3_C_6_H_2_–N), 2.12 (s, 12H, 2,4,6-*Me*_3_C_6_H_2_–N), 1.38 (s, 6H, 4–C*H*_3_C_5_H_2_N). ^13^C­{^1^H} NMR (126 MHz, C_6_D_6_, 298 K) δ 158.4, 155.4, 138.0, 137.4, 134.1, 130.0, 96.6 (*C*H_2_, *meta*-4-Me-pyridine), 21.5 (*C*H_3_, 4-Me-pyridine), 21.1 (*C*H_3_), 19.0 (*C*H_3_). Anal. Calcd for C_48_H_54_N_6_Cl_4_Ge_4_: C, 50.25; H, 4.74; N, 7.33. Found: C, 50.17; H, 4.77; N, 6.93.

#### (GeCl)_4_(μ-κ^1^:κ^1^-DAP^Dep^)_2_ (**8**)

A 20 mL of vial was charged with **3** (0.0342 g, 0.0346 mmol) and GeCl_2_·dioxane (0.0184 g, 0.0795 mmol), and 4 mL of THF was added as solvent. The reaction mixture was allowed to stir for 2 h at room temperature. At this point, all volatiles were removed by vacuo and the residue was extracted into 2 mL of Et_2_O for three times. An orange solid of **8** was obtained in 80.9% yield (0.0337 g, 0.0280 mmol) after all volatiles were removed by vacuo. **Method 2**: In a glovebox, a 20 mL of vial was charged with a solution of **5** (0.0304 g, 0.0301 mmol) in 3 mL of Et_2_O and a magnetic stir bar, and was kept at −35 °C for 30 min. To this solution was slowly added 0.26 mL of 0.12 M GeCl_4_ (0.0312 mmol) in *n*-hexane. The reaction mixture was allowed to warm up to room temperature and stirred overnight. At this point, all volatiles were removed by vacuo and the residue was extracted into 2 mL of *n*-hexane for three times. An orange solid of **8** was obtained in 34.9% yield (0.0126 g, 0.0105 mmol) after all volatiles were removed by vacuo. X-ray quality crystals of **8** were obtained from evaporation of Et_2_O at room temperature. ^1^H NMR (500 MHz, C_6_D_6_, 298 K) δ 7.18–7.11 (m, 12H, 2,6-Et_2_C_6_ *H*_3_), 5.13 (s, 4H, 4-CH_3_C_5_ *H*_2_N), 3.02 (m, 8H, 2,6-(C*H*_2_CH_3_)_2_C_6_H_3_), 2.89 (m, 8H, 2,6-(C*H*_2_CH_3_)_2_C_6_H_3_), 1.31 (s, 6H, 4–C*H*_3_C_5_H_2_N), 1.31 (t, 24H, 2,6-(CH_2_C*H*_3_)_2_C_6_H_3_). ^13^C­{^1^H} NMR (126 MHz, C_6_D_6_, 298 K) δ 159.1, 155.2, 144.2, 135.4, 128.6, 127.2, 97.1 (*C*H_2_, *meta*-4-Me-pyridine), 25.5, 21.4 (*C*H_3_, 4-Me-pyridine), 15.3. Anal. Calcd for C_54_H_62_N_6_Ge_4_Cl_4_: C, 51.90; H, 5.19; N, 6.98. Found: C, 52.09; H, 5.16; N, 6.92.

#### (GeCl)_4_(μ-κ^1^:κ^1^-DAP^Dipp^)_2_ (**9**)

A 20 mL of vial was charged with **1** (0.0557 g, 0.0542 mmol) and GeCl_2_·dioxane (0.0282 g, 0.1219 mmol), and 4 mL of Et_2_O was added as solvent. The reaction mixture was allowed to stir for 2 h at room temperature. At this point, the orange solution was obtained, and the solvent was removed by *vacuo*. The crude material was washed with the mixture of *n*-hexane and Et_2_O in 4:1 ratio to afford **9** as an orange solid after dried by *vacuo* (0.0374 g, 0.0284 mmol, 52.4%). X-ray quality crystals of **9** were obtained from evaporation of Et_2_O at room temperature. ^1^H NMR (500 MHz, C_6_D_6_, 298 K) δ 7.23–7.20 (m, 12H, 2,6-^*i*^Pr_2_C_6_ *H*_3_), 5.04 (s, 4H, 4-CH_3_C_5_ *H*_2_N), 3.66 (septet, 8H, *H*CMe_2_), 1.42 (d, 24H, CH*Me*_2_), 1.31 (s, 6H, 4–C*H*_3_C_5_H_2_N), 1.20 (d, 24H, CH*Me*_2_). ^13^C­{^1^H} NMR (126 MHz, C_6_D_6_, 298 K) δ 159.8, 154.1, 148.9, 134.1, 129.1, 124.6, 97.9 (*C*H_2_, *meta*-4-Me-pyridine), 29.3, 24.8, 21.2 (*C*H_3_, 4-Me-pyridine). Anal. Calcd for C_60_H_78_N_6_Ge_4_Cl_4_: C, 54.78; H, 5.98; N, 6.39. Found: C, 55.231; H, 6.056; N, 6.253.

#### [K­(C_7_H_8_)]_2_Ge_6_(μ_3_-κ^1^:κ^1^:κ^1^-DAP^Dep^)_2_(μ_4_-κ^1^:κ^1^:κ^1^:η^2^-DAP^Dep^)_2_ (**10**)

A 20 mL of vial was charged with **8** (0.0295g, 0.0245 mmol) and KC_8_ (0.0221 g, 0.1635 mmol), and 2 mL of THF was added as solvent. The reaction mixture was allowed to stir for 4 h at room temperature to give a brown suspension. The brown suspension was concentrated under vacuum. The residue was extracted with 2 mL of Et_2_O for three times and filtered through a pad of *Celite* to remove insoluble material. The orange filtrate was concentrated under vacuum to give an orange solid. The crystals of **10** were obtained from evaporation of Et_2_O and few drops of toluene at room temperature (0.0085 g, 0.0038 mmol, 31.0%). **Method 2**: The mixture of **5** (0.0712 g, 0.0720 mmol) and KC_8_ (0.0111 g, 0.0821 mmol) was taken into a 20 mL of vial and 4 mL of THF were added at room temperature. The reaction mixture was allowed to stir for 4 h to give orange suspension. All volatiles were removed by *vacuo*. The residue was extracted with 2 mL of Et_2_O for three times, and the insoluble material was filtered off through a pad of *Celite*. The yellow filtrate was concentrated under vacuum to give a yellow solid (0.0216 g, 0.0096 mmol, 26.7%). X-ray quality crystals of **10** were obtained from evaporation of *n*-hexane and few drops of toluene at room temperature. ^1^H NMR (500 MHz, C_6_D_6_, 298 K) δ 7.27–7.01 (m, 24H, 2,6-^*i*^Pr_2_C_6_H_3_), 5.14 (s, 4H, 4-CH_3_C_5_ *H*_2_N), 4.75 (s, 4H, 4-CH_3_C_5_ *H*_2_N), 3.27–2.18 (m, 32H, 2,6-(C*H*_2_CH_3_)_2_C_6_H_3_), 1.65 (s, 12H, 4–C*H*_3_C_5_H_2_N), 1.17 (t, 48H, 2,6-(CH_2_C*H*_3_)_2_C_6_H_3_). ^13^C­{^1^H} NMR (126 MHz, C_6_D_6_, 298 K) δ 163.6, 163.4, 151.5, 150.4, 143.4, 141.9, 140.6, 136.5, 136.0, 126.6,126.4, 126.2, 125.5, 125.1, 120.8, 97.5 (*C*H_2_, *meta*-4-Me-pyridine), 88.6 (*C*H_2_, *meta*-4-Me-pyridine), 26.9, 24.7, 24.0, 21.7 (*C*H_3_, 4-Me-pyridine), 15.3, 14.5, 14.4. Anal. Calcd for C_104_H_124_N_12_K_2_Ge_6_: C, 60.75; H, 6.08; N, 8.17. Found: C, 61.34; H, 5.98; N, 8.62.

#### [K­(THF)]_2_Ge_6_(μ_3_-κ^1^:κ^1^:κ^1^-DAP^Dipp^)_2_(μ_4_-κ^1^:κ^1^:κ^1^:η^3^-DAP^Dipp^)_2_ (**11**)

The mixture of **10** (0.124 g, 0.0942 mmol) and KC_8_ (0.1138 g, 0.8419 mmol) was taken into a 20 mL of vial and 4 mL of THF were added at room temperature. The reaction mixture was allowed to stir for 4 h to give an orange suspension. All volatiles were removed by *vacuo*. The residue was extracted with 2 mL of *n*-hexane for three times, and the insoluble material was filtered off through a pad of *Celite*. The orange filtrate was concentrated under vacuum to give an orange solid (0.0417 g, 0.0172 mmol, 36.5%). X-ray quality crystals of **11** were obtained from evaporation in a mixture of *n*-hexane and THF at room temperature. ^1^H NMR (500 MHz, C_6_D_6_, 298 K) δ 7.24–7.04 (m, 24H, 2,6-^*i*^Pr_2_C_6_ *H*_3_), 5.32 (s, 4H, 4-CH_3_C_5_ *H*_2_N), 5.18 (s, 4H, 4-CH_3_C_5_ *H*_2_N), 3.65 (septet, 3H, *H*CMe_2_), 3.40 (septet, 2H, *H*CMe_2_), 3.33 (septet, 2H, *H*CMe_2_), 3.13 (septet, 2H, *H*CMe_2_), 3.02 (septet, 3H, *H*CMe_2_), 2.96 (septet, 3H, *H*CMe_2_), 1.47 (d, 12H, CH*Me*_2_), 1.34 (s, 12H, 4–C*H*_3_C_5_H_2_N), 1.29 (d, 12H, CH*Me*_2_), 1.22 (d, 12H, CH*Me*_2_), 1.19 (d, 12H, CH*Me*_2_), 1.13 (d, 12H, CH*Me*_2_), 1.09 (d, 12H, CH*Me*_2_), 1.05 (d, 12H, CH*Me*_2_), 0.77 (d, 12H, CH*Me*_2_). ^13^C­{^1^H} NMR (500 MHz, *d*_8_-THF, 298 K) δ 162.4, 160.9, 153.1, 147.9, 147.4, 147.1, 145.8, 143.1, 142.9, 127.3, 126.9, 125.9, 125.5, 125.0, 124.6, 123.6, 122.6, 101.8 (*C*H_2_, *meta*-4-Me-pyridine), 101.2 (*C*H_2_, *meta*-4-Me-pyridine), 30.2, 28.8, 28.4, 28.3, 26.5, 25.7, 24.5, 24.2, 23.9, 22.9, 21.0 (*C*H_3_, 4-Me-pyridine). Anal. Calcd for C_120_H_156_N_12_Ge_6_K_2_: C, 63.20; H, 6.89; N, 7.37. Found: C, 63.263; H, 7.173; N, 7.074.

#### [K­(THF)]_2_Ge_2_(μ_4_-κ^1^:κ^1^:κ^1^:η^2^-DAP^Dipp^)_2_ (**12**)

**12** was synthesized according to modified literature procedure.^37^ Addition of 5 mL of THF into the reaction mixture of **1** (0.2754g, 0.2677 mmol) and KC_8_ (0.1095 g, 0.8100 mmol) at room temperature resulting in the formation of yellow green suspension immediately. After stirring for 1 h, volatiles were removed under vacuum, and 6 mL of the solution of *n*-hexane was added into the residue. The suspension was filtered through a pad of *Celite*. The filtrate was concentrated by *vacuo* to give an orange powder (0.2214 g, 0.1770 mmol, 66.1%). ^1^H NMR (500 MHz, C_6_D_6_, 298 K) δ 7.24 (d, 2H, *meta*-2,6-^*i*^Pr_2_C_6_ *H*_3_), 7.20 (d, 2H, *meta*-2,6-^*i*^Pr_2_C_6_ *H*_3_), 7.14 (d, 2H, *meta*-2,6-^*i*^Pr_2_C_6_ *H*_3_), 7.07 (t, 2H, *para*-2,6-^*i*^Pr_2_C_6_ *H*_3_), 6.81 (t, 2H, *para*-2,6-^*i*^Pr_2_C_6_ *H*_3_), 6.73 (d, 2H, *meta*-2,6-^*i*^Pr_2_C_6_ *H*_3_), 4.91 (s, 2H, 4-CH_3_C_5_ *H*_2_N), 4.51 (s, 2H, 4-CH_3_C_5_ *H*_2_N), 3.92 (septet, 2H, *H*CMe_2_), 3.50 (septet, 2H, *H*CMe_2_), 3.25 (septet, 2H, *H*CMe_2_), 2.99 (septet, 2H, *H*CMe_2_), 1.65 (s, 6H, 4–C*H*_3_C_5_H_2_N), 1.35 (d, 6H, CH*Me*_2_), 1.35 (d, 6H, CH*Me*_2_), 1.34 (d, 6H, CH*Me*_2_), 1.21 (d, 12H, CH*Me*_2_), 1.10 (d, 6H, CH*Me*_2_), 0.94 (d, 6H, CH*Me*_2_), 0.81 (d, 6H, CH*Me*_2_). ^13^C­{^1^H} NMR (126 MHz, C_6_D_6_, 298 K) δ 163.1, 162.3, 150.6, 150.0, 149.5, 148.2, 145.8, 144.2, 141.1, 125.4, 124.4, 124.1, 123.7, 123.3, 121.2, 96.3 (*C*H_2_, *meta*-4-Me-pyridine), 90.8 (*C*H_2_, *meta*-4-Me-pyridine), 28.6, 28.2, 28.1, 27.8, 27.4, 26.1, 25.8, 25.7, 24.6, 24.5, 23.5, 22.9, 21.2 (*C*H_3_, 4-Me-pyridine). Anal. Calcd for C_60_H_78_N_6_Ge_2_K_2_: C, 65.11; H, 7.10; N, 7.59. Found: C, 65.55; H, 7.34; N, 7.28.

#### (SnCl)_2_Ge_2_(μ_3_-κ^1^:κ^1^:κ^1^-DAP^Dipp^)_2_ (**15**)

A 20 mL of vial was charged with **12** (0.2314g, 0.2091 mmol) and SnCl_2_ (0.0932 g, 0.4915 mmol), and 2 mL of THF was added as solvent. The reaction mixture was allowed to stir for 1 h at room temperature to give a yellow suspension. The yellow suspension was concentrated under vacuum. The residue was extracted with 2 mL of *n*-hexane for three times and filtered through a pad of *Celite* to remove insoluble material. X-ray quality of **15** were obtained from slow evaporation in a mixture of hexane and Et_2_O in 1:2 ratio at room temperature. The crystals were washed with *n*-hexane, and all volatiles were removed by *vacuo* to give a yellow solid (0.0698g, 0.0522 mmol, 24.9%). ^1^H NMR (500 MHz, C_6_D_6_, 298 K) δ 7.22–7.06 (m, 12H, 2,6-^*i*^Pr_2_C_6_ *H*_3_), 5.25 (s, 2H, 4-CH_3_C_5_ *H*_2_N), 4.92 (s, 2H, 4-CH_3_C_5_ *H*_2_N), 3.55 and 3.49 (septet, 4H, *H*CMe_2_), 3.52–3.47 (septet, 2H, *H*CMe_2_), 3.07 (septet, 2H, *H*CMe_2_), 2.35 (septet, 2H, *H*CMe_2_), 1.40 (d, 6H, CH*Me*_2_), 1.33 (d, 6H, CH*Me*_2_), 1.31 (s, 6H, 4–C*H*_3_C_5_H_2_N), 1.20 (d, 6H, CH*Me*_2_), 1.17 (d, 6H, CH*Me*_2_), 1.13 (d, 6H, CH*Me*_2_), 1.07 (d, 6H, CH*Me*_2_), 0.85 (d, 6H, CH*Me*_2_), 0.79 (d, 6H, CH*Me*_2_). ^13^C­{^1^H} NMR (126 MHz, C_6_D_6_, 298 K) δ 161.5, 161.4, 153.7, 147.5, 147.4, 145.8, 145.1, 141.7, 141.3, 129.5, 127.3, 126.1, 125.6, 125.2, 124.7, 100.9 (*C*H_2_, *meta*-4-Me-pyridine), 97.3 (*C*H_2_, *meta*-4-Me-pyridine), 30.2, 28.7, 28.1, 27.9, 27.4, 26.4, 26.1, 25.9, 24.8, 24.4, 24.1, 23.5, 20.8 (*C*H_3_, 4-Me-pyridine). ^119^Sn­{^1^H} NMR (187 MHz, C_6_D_6_, 298 K) δ −245.26. Anal. Calcd for C_60_H_78_N_6_Ge_2_Sn_2_Cl_2_: C, 53.91; H, 5.88; N, 6.29. Found: C, 54.30; H, 6.09; N, 6.20.

#### [K­(Et_2_O)]_2_Ge_4_Sn_2_(μ_3_-κ^1^:κ^1^:κ^1^-DAP^Dipp^)_2_(μ_4_-κ^1^:κ^1^:κ^1^:η^2^-DAP^Dipp^)_2_ (**16**)

The mixture of 15 (0.1641 g, 0.1227 mmol) and 0.5 wt % K/Hg (2.8876 g, 0.3694 mmol) was taken into a 20 mL of vial and 6 mL of the solution of Et_2_O and THF in 2:1 ratio was added at room temperature. The reaction mixture was allowed to stir for 3 h to give earthy yellow suspension. The crude material was collected after filtration, and all volatiles were removed by vacuo. The residue was extracted with 2 mL of *n*-hexane for three times, and the insoluble material was filtered off through a pad of Celite. The orange filtrate was concentrated under vacuum to give an earthy yellow solid (0.1128 g, 0.0447 mmol, 72.9%). X-ray quality crystals of 16 were obtained from slow evaporation in a mixture of *n*-hexane and Et_2_O at room temperature. ^1^H NMR (500 MHz, C_6_D_6_, 298 K) δ 7.24–7.06 (m, 24H, 2,6-^i^Pr_2_C_6_H_3_), 5.30 (s, 2H, 4-CH_3_C_5_H_2_N), 5.24 (s, 2H, 4-CH_3_C_5_H_2_N), 5.22 (s, 2H, 4-CH_3_C_5_H_2_N), 5.13 (s, 2H, 4-CH_3_C_5_H_2_N), 3.54 (septet, 2H, HCMe_2_), 3.37 (septet, 2H, HCMe_2_), 3.07 (septet, 12H, HCMe_2_), 1.45 (d, 12H, CHMe_2_), 1.42 (d, 12H, CHMe_2_), 1.34 (s, 12H, 4-CH_3_C_5_H_2_N), 1.28 (d, 24H, CHMe_2_), 1.16 (d, 12H, CHMe_2_), 1.13 (d, 24H, CHMe_2_), 0.77 (d, 12H, CHMe_2_). ^13^C­{^1^H} NMR (126 MHz, C_6_D_6_, 298 K) δ 162.4, 160.9, 153.1, 147.9, 147.4, 147.1, 145.8, 143.1, 142.9, 127.3, 126.9, 125.9, 125.5, 125.0, 124.6, 123.6, 122.6, 101.8 (*C*H_2_, *meta*-4-Me-pyridine), 101.2 (*C*H_2_, *meta*-4-Me-pyridine), 30.2, 28.8, 28.4, 26.5, 25.7, 24.5, 24.2, 23.9, 22.9, 21.0 (*C*H_3_, 4-Me-pyridine). ^119^Sn­{^1^H} NMR (187 MHz, C_6_D_6_, 298 K) δ −94.66. Anal. Calcd for C_120_H_156_N_12_Ge_4_Sn_2_K_2_: C, 60.74; H, 6.63; N, 7.08. Found: C, 61.35; H, 6.87; N, 7.08.

### X-ray crystallography

Data collection of **2**–**6, 8**, **9**, **11** and **15**–**16** were carried out using the SMART program on a Bruker SMART Apex II diffractometer with CCD area detector and multilayer mirror monochromated Mo Kα radiation (λ = 0.71073 Å) at 200(2) K or Cu Kα radiation (λ = 1.54184 Å) at 100(1) K. Cell parameters were retrieved and refined using *DENZO-SMN* software^91^ on all observed reflections. Data reduction was performed with the *DENZO-SMN* software as well. An empirical absorption was based on the symmetry-equivalent reflections and applied the data using the *SORTAV* program.^92^ Using *SHELXTL* program^93^ on PC computer made the structure analysis. The structure was solved by using the *SHELXS-97* program and refined by using *SHELXL-97* program by full-matrix least-squares on *F* ^2^ values.^94–96^ All of non-hydrogen atoms are refined anisotropically. Hydrogen atoms attached to the carbons were fixed at calculated positions and refined using a riding mode. **10** was collected on Rigaku XtaLAB HyPix-Arc 150 diffractometer with Cu–Kα radiation (λ = 1.54178 Å) at 100(10) K. The structure determinations and refinements were carried out using the *SHELXS* ^95^ and *SHELXL* ^96^ programs respectively on the Olex2 interface.^97^ The structures were solved using direct methods, which yielded the positions of all nonhydrogen atoms. Hydrogen atoms were placed in calculated positions in the final structure refinement. Crystallographic refinement parameters are listed in Tables S1–S3.

### Computational Methods

Calculations were performed with the Gaussian 16 software package.^98^ The molecular geometries were optimized without symmetry constraints at the BP86 level of density functional theory (DFT)^99,100^ and stability of wave function was checked for optimized structure. Vibrational frequency calculations at the same level of theory have also been performed at 1 atm and 298.15 K to identify all the located stationary points as minima (zero imaginary frequency) or transition states (one imaginary frequency). The SCF convergence criterion was set to 10^–8^ in all cases. Intrinsic reaction coordinates (IRC) were calculated for the transition states to validate the expected reactants and products.^101,102^ The 6–31G­(d,p) Pople basis set was used to describe H, C, N, Cl, K and Ge atoms (named BS-I).^103–108^ The basis set used for the single-point calculations comprised def2-TZVP for all atoms (named BS-II)^109^ with the optimized structures at the BP86/BS-I level. Multiwfn 3.8 (dev) program^110^ was used for the analyses of natural bond orbitals (NBO)^111^ and electron localization function (ELF)^112^ and the extended transition state method for energy decomposition analysis combined with the natural orbitals for chemical valence (ETS-NOCV).^55^ Cartesian coordinates of the optimized geometries are listed in Table S12. To speed up the calculations for the intramolecular rearrangement of 5, the bulky 2,6-Et_2_(C_6_H_3_) groups of the 2,6-diamidopyridyl ligands were replaced with the methyl groups.

## Supplementary Material



## Notes

NMR spectra of all compounds, preparative procedures, crystallographic and computational data (PDF)
