# Supplementary material for: sp-Hybridized Seesaw Ge0 Complexes via Germylone-to-Seesaw Isomerization in a Four-Electron Cyclic N2Ge2 Ligand
Source: Inorg Chem. 2025 Sep 12;64(38):19181–96. doi: 10.1021/acs.inorgchem.5c02120 (PMC12486199; doi:10.1021/acs.inorgchem.5c02120)
Supplement: Supplementary file 1 [file ic5c02120_si_001.pdf]

Supporting Information

sp-Hybridized Seesaw Ge^0 Complexes via Germylone-to-Seesaw Isomerization in a Four-electron Cyclic N_2Ge_2 Ligand

Wei-Ting Kuo,[a‡] Cian-Wei Yang,[a‡] Gou-Tao Huang,[b‡] Yu-Te Wey [a], Fan-Shan Yang,[a] Hsien-Cheng Yu,[a] Jen-Shiang K. Yu[b]* and Yi-Chou Tsai[a]*

[a] *Department of Chemistry, National Tsing Hua University, Hsinchu 300313, Taiwan, Republic of China.*

[b] *Department of Biological Science and Technology, Institute of Bioinformatics and Systems Biology and Center for Intelligent Drug Systems and Smart Bio-devices (IDSB), National Yang Ming Chiao Tung University, Hsinchu 300, Taiwan, Republic of China.*

‡ The authors contributed equally to this work.

Jen-Shiang K. Yu–Email: jsyu@mail.nctu.edu.tw

Yi-Chou Tsai–Email: yictsai@mx.nthu.edu.tw

Table of contents

1. General considerations.......1

2. Details of crystal data.......13

3. NMR spectra.......28

4. Elemental analysis report.......44

5. Details of computational studies.......58

6. References.......116

1. General considerations

All manipulations were carried out using standard Schlenk and glove box techniques under an atmosphere of high-purity nitrogen. Diethyl ether (Et_2O) and tetrahydrofuran (THF) were distilled under nitrogen from purple sodium benzophenone ketyl. *n*-Pentane, *n*-hexane and toluene were passed through columns of solvent purification systems (Vigor VAPA-5) to remove oxygen and moisture. Distilled solvents were transferred under vacuum into vacuum-tight glass vessels before being transferred into a glove box. C_6D_6 and d_8-THF were purchased in ampoules from Sigma-Aldrich and stored over 4 Å molecular sieves in Schlenk tubes. 4 Å molecular sieves and *celite* were dried in a vacuum at 200 ℃ for 3 days. All other commercially available chemicals were used without further purification. Elemental analyses were performed with the Elementar vario EL CUBE CHN-OS Rapid. The ^1H, $^{13}C\{^1H\}$ and $^{119}Sn\{^1H\}$ NMR spectra were recorded with Varian Unity INOVA-500MHz, Varian Unity INOVA-400MHz and Bruker Avance -500MHz spectrometer and referenced internally the residue of the solvent resonances (C_6D_6: ^1H: 7.16 ppm, $^{13}C\{^1H\}$: 128.06 ppm; d_8-THF: ^1H: 1.72 and 3.58 ppm, $^{13}C\{^1H\}$: 25.31 and 67.21 ppm).[1] $^{119}Sn\{^1H\}$ NMR spectra were referenced externally with respect to $SnMe_4$. The dilithiated 2,6-diamidopyridines $Li_2[(DAP^{Ar})]$ (DAP^{Ar} = 2,6-$(ArN)_2$-4-$CH_3C_5H_2N$); Ar = Dipp, Dep, Mes; Dipp = 2,6-$^iPr_2C_6H_3$, Dep = 2,6-$Et_2C_6H_3$, Mes = 2,4,6-$Me_3C_6H_2$),[2–3] KC_8,[4] and K/Hg[5] were synthesized following the documented methods.

Synthesis of $Ge_2(\mu$-κ^1:κ^2-$DAP^{Dipp})_2$ (1)

1 was synthesized according to modified literature procedure.[3] A 20 mL of vial was charged with $Li_2[(DAP^{Dipp})]$ (0.0922g, 0.1741 mmol) and $GeCl_2$□dioxane (0.0433 g, 0.1870 mmol), and 2 mL of THF was added as solvent. The reaction mixture was allowed to stir for 1 hour at room temperature. At this point, the yellow solution was obtained, and the solvent was removed by *vacuo*. The residue was extracted with 2 mL of *n*-hexane for three times and filtered through a pad of *Celite* to remove insoluble material. The yellow filtrate was concentrated under vacuum to give a yellow solid (0.0742 g, 0.0721 mmol, 82.8%). ^1H NMR (500 MHz, C_6D_6, 298K) δ 7.26-7.03 (m, 12H, 2,6-$^iPr_2C_6H_3$), 5.18 (s, 2H, 4-$CH_3C_5H_2N$), 4.96 (s, 2H, 4-$CH_3C_5H_2N$), 3.64 (septet, 4H, $HCMe_2$), 3.50 (septet, 2H, $HCMe_2$), 2.34

(septet, 2H, *H*CMe$_2$), 1.39 (s, 6H, 4-C*H*$_3$C$_5$H$_2$N), 1.37 (d, 6H, CH*Me*$_2$), 1.36 (d, 6H, CH*Me*$_2$), 1.33 (d, 6H, CH*Me*$_2$), 1.17 (d, 6H, CH*Me*$_2$), 1.14 (d, 6H, CH*Me*$_2$), 1.06 (d, 6H, CH*Me*$_2$), 0.80 (d, 6H, CH*Me*$_2$), 0.75 (d, 6H, CH*Me*$_2$). ^{13}C{^{1}H} NMR (126 MHz, C$_6$D$_6$, 298K) δ 169.0, 158.4, 152.1, 148.7, 147.8, 147.3, 145.7, 139.9, 139.2, 127.6, 126.9, 125.0, 125.0, 124.6, 123.6, 99.2 (*C*H$_2$, *meta*-4-Me-pyridine), 94.9 (*C*H$_2$, *meta*-4-Me-pyridine), 30.2, 29.9, 28.7, 27.9, 27.6, 26.9, 25.9, 25.2, 24.9, 24.8, 24.3, 24.2, 21.5 (*C*H$_3$, 4-Me-pyridine). Anal. Calcd. for $C_{60}H_{78}N_6Ge_2$: C, 70.06; H, 7.64; N, 8.17. Found: C, 69.57; H, 7.71; N, 7.91.

Synthesis of Ge$_2$(μ-κ^1:κ^2-DAP^{Mes})$_2$ (2)

The mixture of Li$_2$[(DAP^{Mes})] (0.2070g, 0.4646 mmol) and GeCl$_2$□dioxane (0.1145 g, 0.4946 mmol) was taken into a 20 mL of vial and 2 mL of THF were added at room temperature. The reaction mixture was allowed to stir for 1 hour. All volatiles were removed by *vacuo*. The residue was extracted with 2 mL of toluene for three times, and the insoluble material was filtered off through a pad of *Celite*. The yellow filtrate was concentrated under vacuum to give a yellow solid (0.1079 g, 0.1254 mmol, 53.9%). X-ray quality crystals of **2** were obtained from evaporation of toluene at room temperature. ^{1}H NMR (500 MHz, C$_6$D$_6$, 298K) δ 6.89 (s, 2H, 2,4,6-Me$_3$C$_6$*H*$_2$), 6.73 (s, 4H, 2,4,6-Me$_3$C$_6$*H*$_2$), 6.66 (s, 2H, 2,4,6-Me$_3$C$_6$*H*$_2$), 5.37 (s, 2H, 4-CH$_3$C$_5$*H*$_2$N), 5.02 (s, 2H, 4-CH$_3$C$_5$*H*$_2$N), 2.56 (s, 6H, 2,4,6-*Me*$_3$C$_6$H$_2$), 2.38 (s, 6H, 2,4,6-*Me*$_3$C$_6$H$_2$), 2.19 (s, 6H, 2,4,6-*Me*$_3$C$_6$H$_2$), 2.16 (s, 6H, 2,4,6-*Me*$_3$C$_6$H$_2$), 2.06 (s, 6H, 2,4,6-*Me*$_3$C$_6$H$_2$), 1.60 (s, 6H, 2,4,6-*Me*$_3$C$_6$H$_2$), 1.49 (s, 6H, 4-C*H*$_3$C$_5$H$_2$N). ^{13}C{^{1}H}} NMR (126MHz, C$_6$D$_6$, 298K) δ 166.9, 157.3, 152.5, 141.4, 138.5, 137.2, 137.0, 134.9, 134.6, 134.4, 130.5, 129.7, 129.4, 128.5, 97.8 (*C*H$_2$, *meta*-4-Me-pyridine), 93.0 (*C*H$_2$, *meta*-4-Me-pyridine), 21.8 (*C*H$_3$, 4-Me-pyridine), 20.9 (*C*H$_3$), 20.9 (*C*H$_3$,), 20.7 (*C*H$_3$), 20.3 (*C*H$_3$), 19.4 (*C*H$_3$), 17.2 (*C*H$_3$). Anal. Calcd. for $C_{48}H_{54}N_6Ge_2$: C, 67.02; H, 6.33; N, 9.77. Found: C, 67.70; H, 6.80; N, 9.46.

Synthesis of Ge$_2$(μ-κ^1:κ^2-DAP^{Dep})$_2$ (3)

Addition of 2 mL of THF into the reaction mixture of Li$_2$[(DAP^{Dep})] (0.1010 g, 0.2133 mmol) and GeCl$_2$□dioxane (0.0537 g, 0.2320 mmol) at room temperature resulting in the formation of yellow solution immediately. After stirring for 1 hour, volatiles were removed under vacuum, and then 6 mL of

n-hexane was added into the residue. The yellow suspension was filtered through a pad of *Celite*. The filtrate was concentrated by *vacuo* to give a yellow solid (0.0643 g, 0.0701 mmol, 65.7%). X-ray quality crystals of **3** were obtained from recrystallization in *n*-hexane at −30 °C or evaporation of toluene at room temperature. ^{1}H NMR (500 MHz, C_6D_6, 298K) δ 7.24-6.95 (m, 12H, 2,6-$Et_2C_6\mathit{H}_3$), 5.28 (s, 4H, 4-$CH_3C_5\mathit{H}_2N$), 4.95 (s, 4H, 4-$CH_3C_5\mathit{H}_2N$), 3.10 and 3.01 (m, 8H, 2,6-$(C\mathit{H}_2CH_3)_2C_6H_3$), 2.84 (m, 2H, 2,6-$(C\mathit{H}_2CH_3)_2C_6H_3$), 2.61 (m, 4H, 2,6-$(C\mathit{H}_2CH_3)_2C_6H_3$), 2.05 (m, 2H, 2,6-$(C\mathit{H}_2CH_3)_2C_6H_3$), 1.46 (m, 2H, 2,6-$(C\mathit{H}_2CH_3)_2C_6H_3$), 1.40 (s, 6H, 4-$C\mathit{H}_3C_5H_2N$), 1.27 (t, 6H, 2,6-$(CH_2C\mathit{H}_3)_2C_6H_3$), 1.21 (t, 6H, 2,6-$(CH_2C\mathit{H}_3)_2C_6H_3$), 1.02 (t, 6H, 2,6-$(CH_2C\mathit{H}_3)_2C_6H_3$), 0.85 (t, 6H, 2,6-$(CH_2C\mathit{H}_3)_2C_6H_3$). $^{13}C\{^{1}H\}$ NMR (126 MHz, C_6D_6, 298K) δ 167.8, 157.7, 152.5, 143.3, 142.8, 142.3, 141.5, 141.1, 140.1, 127.2, 126.8, 126.4, 126.3, 125.7, 98.3 (*C*H_2, *meta*-4-Me-pyridine), 93.4 (*C*H_2, *meta*-4-Me-pyridine), 26.4, 26.1, 24.2, 22.6, 21.7 (*C*H_3, 4-Me-pyridine), 17.1, 16.2, 15.1, 13.9. Anal. Calcd. for $C_{52}H_{62}N_6Ge_2$: C, 68.16; H, 6.82; N, 9.17. Found: C, 68.579; H, 7.142; N, 9.061.

Synthesis of *(μ-Ge)(κ²-N₂Ge₂^Mes)* (4)

Method 1: The mixture of **2** (0.1675 g, 0.1947 mmol) and KC_8 (0.0543 g, 0.4017 mmol) was taken into a 20 mL of vial and 4 mL of THF were added at room temperature. The reaction mixture was allowed to stir for 1 hour to give a yellow green suspension. All volatiles were removed by *vacuo*. The residue was extracted with 2 mL of *n*-hexane for three times, and the insoluble material was filtered off through a pad of *Celite*. The yellow filtrate was concentrated under vacuum to give a yellow solid (0.0515 g, 0.0552 mmol, 28.4%).

Method 2: In a glove box, a 20-mL vial was charged with **2** (0.0674 g, 0.0783 mmol), Ge powder (0. 114 g, 1.566 mmol), 1 mL of Et_2O and a magnetic stir bar. The reaction mixture was allowed to stir for 7 days. At this point, the suspension was filtered through a pad of Celite to remove insoluble material. The filtrate was evaporated by *vacuo* and the residue was extracted into 2 mL of *n*-pentane for three times. A yellow solid of 4 was isolated in 22.8% yield (0.0167 g, 0.0179 mmol) after all volatiles were removed by *vacuo*.

Method 3: A 20 mL of vial was charged with **7** (0.0743 g, 0.0648 mmol) and KC_8 (0.0424 g, 0.3136

mmol), and 2 mL of Et_2O was added as solvent. The reaction mixture was allowed to stir for 4 hours at room temperature. At this point, the brown suspension was obtained, and the solvent was removed by *vacuo*. The residue was extracted with 2 mL of *n*-hexane for three times and filtered through a pad of *Celite* to remove the black insoluble material. The brown filtrate was concentrated under vacuum to give a brown solid (0.037 g, 0.0397 mmol, 61.3%). X-ray quality crystals of **4** were obtained from evaporation of *n*-hexane at room temperature. ^1H NMR (500 MHz, C_6D_6, 298K) δ 6.82 (s, 4H, 2,4,6-$Me_3C_6H_2$-N), 6.80 (s, 4H, 2,4,6-$Me_3C_6H_2$-N), 5.36 (s, 4H, 4-$CH_3C_5H_2N$), 2.28 (s, 12H, 2,4,6-$Me_3C_6H_2$-N), 2.14 (s, 12H, 2,4,6-$Me_3C_6H_2$-N), 2.00 (s, 12H, 2,4,6-$Me_3C_6H_2$-N), 1.41 (s, 6H, 4-$CH_3C_5H_2N$). $^{13}C\{^1H\}$ NMR (126 MHz, C_6D_6, 298K) δ 160.3, 154.4, 143.0, 136.8, 135.3, 135.2, 130.4, 130.2, 97.8 (CH_2, *meta*-4-Me-pyridine), 21.3 (CH_3, *meta*-4-Me-pyridine), 21.0 (CH_3), 19.5 (CH_3), 18.4 (CH_3). Anal. Calcd. for $C_{48}H_{54}N_6Ge_3$: C, 61.80; H, 5.83; N, 9.01. Found: C, 61.45; H, 6.00; N, 8.66.

Synthesis of *(μ-Ge)(κ²-N₂Ge₂ᴰᵉᵖ) (5)*

Method 1: A 20 mL of vial was charged with **3** (0.0504 g, 0.0550 mmol) and KC_8 (0.0092 g, 0.0681 mmol), and the mixture of *n*-hexane and Et_2O in 2:1 ratio was added as solvent. The reaction mixture was allowed to stir for 4 hours at room temperature. At this point, the reddish-brown suspension was obtained, and the solvent was removed by *vacuo*. The residue was extracted with 2 mL of Et_2O for three times and filtered through a pad of *Celite* to remove the black insoluble material. The brown filtrate was concentrated under vacuum to give a brown solid (0.0282 g, 0.0285 mmol, 51.8%).

Method 2: In a glove box, a 20-mL vial was charged with **3** (0.0504 g, 0.0550 mmol), Ge powder (0.0800 g, 1.10 mmol), 1.5 mL of Et_2O and a magnetic stir bar. The reaction mixture was allowed to stir for 7 days. At this point, the suspension was filtered through a pad of *Celite* to remove insoluble material. The filtrate was evaporated by *vacuo* and the residue was extracted into 2 mL of *n*-pentane for three times. A brown solid of **5** was isolated in 31.2% yield (0.0170 g, 0.0172 mmol) after all volatiles were removed by *vacuo*.

Method 3: A 20 mL of vial was charged with **8** (0.3070 g, 0.2551 mmol) and KC_8 (0.1930 g, 1.4277 mmol), and 8 mL of Et_2O was added as solvent. The reaction mixture was allowed to stir for 1 hour at

room temperature. At this point, the brown suspension was obtained, and the solvent was removed by *vacuo*. The residue was extracted with 2 mL of Et_2O for three times and filtered through a pad of *Celite* to remove the black insoluble material. The brown filtrate was concentrated under vacuum to give a brown solid (0.2140 g, 0.2164 mmol, 84.8%). X-ray quality crystals of **5** were obtained from evaporation of *n*-hexane at room temperature. ^{1}H NMR (500 MHz, C_6D_6, 298K) δ 7.15-7.09 (m, 12H, 2,6-$Et_2C_6H_3$), 5.24 (s, 4H, 4-$CH_3C_5H_2N$), 2.78 and 2.70 (m, 8H, 2,6-$(CH_2CH_3)_2C_6H_3$), 2.40 (m, 8H, 2,6-$(CH_2CH_3)_2C_6H_3$), 1.34 (s, 6H, 4-$CH_3C_5H_2N$), 1.13 (t, 12H, 2,6-$(CH_2CH_3)_2C_6H_3$), 1.01(t, 12H, 2,6-$(CH_2CH_3)_2C_6H_3$). ^{13}C{^{1}H} NMR (126 MHz, C_6D_6, 298K) δ 160.8, 154.0, 144.3, 142.1, 141.4, 127.5, 126.6, 126.3, 98.7 (CH_2, *meta*-4-Me-pyridine), 25.8, 23.5, 21.0 (CH_3, 4-Me-pyridine), 15.5, 14.0. Anal. Calcd. for $C_{52}H_{62}NGe_3$: C, 63.15; H, 6.32; N, 8.50. Found: C, 62.59; H, 6.39; N, 8.30.

Synthesis of *[(Cl₃In)←(μ-Ge)(κ²-N₂Ge₂^Dep)]₂* (6)

Addition of 2 mL of THF into the reaction mixture of **5** (0.1281 g, 0.1295 mmol) and $InCl_3$ (0.0626 g, 0.2830 mmol) at room temperature resulting in the formation of orange solution immediately. After stirring for 1 hour, volatiles were removed under vacuum, and 6 mL of the solution of *n*-hexane and toluene in 1:1 ratio was added into the residue. The yellow suspension place in the freezer at −30 °C and was filtered through a pad of *Celite*. The filtrate was concentrated by *vacuo.* Yellow crystals of **6** were obtained from recrystallization in Et_2O at −30 °C. The crystals were washed with cold Et_2O to afford **6** as a yellow solid after dried by *vacuo* (0.064 g, 0.0529 mmol, 40.8%). X-ray quality crystals of **6** were obtained from evaporation of C_6D_6 at room temperature or recrystallization in Et_2O at −30 °C. ^{1}H NMR (400 MHz, C_6D_6, 298K) δ 7.10 (m, 12H, 2,6-$^iPr_2C_6H_3$), 5.36 (s, 4H, 4-$CH_3C_5H_2N$), 2.81 and 2.26 (m, 16H, 2,6-$(CH_2CH_3)_2C_6H_3$), 1.25 (t, 12H, 2,6-$(CH_2CH_3)_2C_6H_3$), 1.17 (s, 6H, 4-$CH_3C_5H_2N$), 0.96 (t, 12H, 2,6-$(CH_2CH_3)_2C_6H_3$). ^{13}C{^{1}H} NMR (101 MHz, C_6D_6, 298K) δ 159.9, 155.6, 142.1, 141.8, 126.4, 101.1 (CH_2, *meta*-4-Me-pyridine), 26.4, 23.6, 20.9 (CH_3, 4-Me-pyridine), 15.4, 13.7. Anal. Calcd. for $C_{52}H_{62}N_6InCl_3Ge_3$: C, 51.61; H, 5.16; N, 6.94. Found: C, 51.25; H, 5.09; N, 6.71.

Synthesis of $(GeCl)_4(\mu\text{-}\kappa^1\text{:}\kappa^1\text{-DAP}^{Mes})_2$ (7)

Method 1: A 20 mL of vial was charged with **2** (0.1004 g, 0.1167 mmol) and $GeCl_2$·dioxane (0.0566 g,

0.2445 mmol), and 4 mL of THF was added as solvent. The reaction mixture was allowed to stir for two hours at room temperature. At this point, the orange solution was obtained, and the solvent was removed by *vacuo*. The crude material was washed with the mixture of *n*-hexane and Et_2O in 1:1 ratio to afford **7** as an orange solid after dried by *vacuo* (0.0599 g, 0.0522 mmol, 44.7 %).

Method 2: In a glovebox, a 20-mL of vial was charged with a solution of **4** (0.0629 g, 0.0674 mmol) in 3 mL of THF. To this solution was slowly added 0.51 mL of 0.12 M $GeCl_4$ (0.0612 mmol) in *n*-hexane. The reaction mixture was allowed to stir overnight at room temperature. At this point, all volatiles were removed by vacuo and the residue was extracted into 2 mL of *n*-hexane for three times. An orange solid of **7** was obtained in 24.5% yield (0.0188 g, 0.0165 mmol) after all volatiles were removed by vacuo. ^{1}H NMR (500 MHz, C_6D_6, 298K) δ 6.82 (s, 8H, 2,4,6-$Me_3C_6H_2$-N), 5.20 (s, 4H, 4-$CH_3C_5H_2N$), 2.50 (s, 24H, 2,4,6-$Me_3C_6H_2$-N), 2.12 (s, 12H, 2,4,6-$Me_3C_6H_2$-N), 1.38 (s, 6H, 4-$CH_3C_5H_2N$). $^{13}C\{^{1}H\}$ NMR (126 MHz, C_6D_6, 298K) δ 158.4, 155.4, 138.0 , 137.4, 134.1, 130.0, 96.6 (CH_2, *meta*-4-Me-pyridine), 21.5 (CH_3, 4-Me-pyridine), 21.1 (CH_3), 19.0 (CH_3). Anal. Calcd. for $C_{48}H_{54}N_6Cl_4Ge_4$: C, 50.25; H, 4.74; N, 7.33. Found: C, 50.17; H, 4.77; N, 6.93.

Synthesis of $(GeCl)_4(\mu\text{-}\kappa^1\text{:}\kappa^1\text{-DAP}^{Dep})_2$ (8)

Method 1: A 20 mL of vial was charged with **3** (0.0342 g, 0.0346 mmol) and $GeCl_2 \cdot$dioxane (0.0184 g, 0.0795 mmol), and 4 mL of THF was added as solvent. The reaction mixture was allowed to stir for two hours at room temperature. At this point, all volatiles were removed by vacuo and the residue was extracted into 2 mL of Et_2O for three times. An orange solid of **8** was obtained in 80.9 % yield (0.0337 g, 0.0280 mmol) after all volatiles were removed by vacuo.

Method 2: In a glovebox, a 20-mL of vial was charged with a solution of **5** (0.0304 g, 0.0301 mmol) in 3 mL of Et_2O and a magnetic stir bar, and was kept at −35 °C for 30 minutes. To this solution was slowly added 0.26 mL of 0.12 M $GeCl_4$ (0.0312 mmol) in *n*-hexane. The reaction mixture was allowed to warm up to room temperature and stirred overnight. At this point, all volatiles were removed by vacuo and the residue was extracted into 2 mL of *n*-hexane for three times. An orange solid of **8** was obtained in 34.9% yield (0.0126 g, 0.0105 mmol) after all volatiles were removed by vacuo. X-ray quality crystals of **8** were

obtained from evaporation of Et_2O at room temperature. ^{1}H NMR (500 MHz, C_6D_6, 298K) δ 7.18-7.11 (m, 12H, 2,6-$Et_2C_6\mathit{H}_3$), 5.13 (s, 4H, 4-$CH_3C_5\mathit{H}_2N$), 3.02 (m, 8H, 2,6-$(C\mathit{H}_2CH_3)_2C_6H_3$), 2.89 (m, 8H, 2,6-$(C\mathit{H}_2CH_3)_2C_6H_3$), 1.31 (s, 6H, 4-$C\mathit{H}_3C_5H_2N$), 1.31 (t, 24H, 2,6-$(CH_2C\mathit{H}_3)_2C_6H_3$). $^{13}C\{^1H\}$ NMR (126 MHz, C_6D_6, 298K) δ 159.1, 155.2, 144.2, 135.4, 128.6, 127.2, 97.1 (*C*H_2, *meta*-4-Me-pyridine), 25.5, 21.4 (*C*H_3, 4-Me-pyridine), 15.3. Anal. Calcd. for $C_{54}H_{62}N_6Ge_4Cl_4$: C, 51.90; H, 5.19; N, 6.98. Found: C, 52.09; H, 5.16; N, 6.92.

Synthesis of (GeCl)$_4$(μ-κ^1:κ^1-DAP^{Dipp})$_2$ (9)

A 20 mL of vial was charged with **1** (0.0557 g, 0.0542 mmol) and $GeCl_2$·dioxane (0.0282 g, 0.1219 mmol), and 4 mL of Et_2O was added as solvent. The reaction mixture was allowed to stir for two hours at room temperature. At this point, the orange solution was obtained, and the solvent was removed by *vacuo*. The crude material was washed with the mixture of *n*-hexane and Et_2O in 4:1 ratio to afford **9** as an orange solid after dried by *vacuo* (0.0374 g, 0.0284 mmol, 52.4 %). X-ray quality crystals of **9** were obtained from evaporation of Et_2O at room temperature. ^{1}H NMR (500 MHz, C_6D_6, 298K) δ 7.23-7.20 (m, 12H, 2,6-$^iPr_2C_6\mathit{H}_3$), 5.04 (s, 4H, 4-$CH_3C_5\mathit{H}_2N$), 3.66 (septet, 8H, *H*CMe$_2$), 1.42 (d, 24H, CH*Me*$_2$), 1.31 (s, 6H, 4-$C\mathit{H}_3C_5H_2N$), 1.20 (d, 24H, CH*Me*$_2$). $^{13}C\{^1H\}$ NMR (126 MHz, C_6D_6, 298K) δ 159.8, 154.1, 148.9, 134.1, 129.1, 124.6, 97.9 (*C*H_2, *meta*-4-Me-pyridine), 29.3, 24.8, 21.2 (*C*H_3, 4-Me-pyridine). Anal. Calcd. for $C_{60}H_{78}N_6Ge_4Cl_4$: C, 54.78; H, 5.98; N, 6.39. Found: C, 55.231; H, 6.056; N, 6.253.

Synthesis of [K(C_7H_8)]$_2$Ge$_6$(μ_3-κ^1:κ^1:κ^1-DAP^{Dep})$_2$(μ_4-κ^1:κ^1:κ^1:η^2-DAP^{Dep})$_2$ (10)

Method 1: A 20 mL of vial was charged with **8** (0.0295g, 0.0245 mmol) and KC_8 (0.0221 g, 0.1635 mmol), and 2 mL of THF was added as solvent. The reaction mixture was allowed to stir for 4 hours at room temperature to give a brown suspension. The brown suspension was concentrated under vacuum. The residue was extracted with 2 mL of Et_2O for three times and filtered through a pad of *Celite* to remove insoluble material. The orange filtrate was concentrated under vacuum to give an orange solid. The crystals of **10** were obtained from evaporation of Et_2O and few drops of toluene at room temperature (0.0085 g, 0.0038 mmol, 31.0 %).

Method 2: The mixture of **5** (0.0712 g, 0.0720 mmol) and KC_8 (0.0111 g, 0.0821 mmol) was taken into

a 20 mL of vial and 4 mL of THF were added at room temperature. The reaction mixture was allowed to stir for 4 hours to give orange suspension. All volatiles were removed by *vacuo*. The residue was extracted with 2 mL of Et_2O for three times, and the insoluble material was filtered off through a pad of *Celite*. The yellow filtrate was concentrated under vacuum to give a yellow solid (0.0216 g, 0.0096 mmol, 26.7%). X-ray quality crystals of **10** were obtained from evaporation of *n*-hexane and few drops of toluene at room temperature. ^{1}H NMR (500 MHz, C_6D_6, 298K) δ 7.27-7.01 (m, 24H, 2,6-^{i}$Pr_2C_6H_3$), 5.14 (s, 4H, 4-$CH_3C_5\mathit{H}_2N$), 4.75 (s, 4H, 4-$CH_3C_5\mathit{H}_2N$), 3.27-2.18 (m, 32H, 2,6-$(C\mathit{H}_2CH_3)_2C_6H_3$), 1.65 (s, 12H, 4-$C\mathit{H}_3C_5H_2N$), 1.17 (t, 48H, 2,6-$(CH_2C\mathit{H}_3)_2C_6H_3$). ^{13}C{^{1}H} NMR (126 MHz, C_6D_6, 298K) δ 163.6, 163.4, 151.5, 150.4, 143.4, 141.9, 140.6, 136.5, 136.0, 126.6,126.4, 126.2, 125.5, 125.1, 120.8, 97.5 ($\mathit{C}H_2$, *meta*-4-Me-pyridine), 88.6 ($\mathit{C}H_2$, *meta*-4-Me-pyridine), 26.9, 24.7, 24.0, 21.7 ($\mathit{C}H_3$, 4-Me-pyridine), 15.3, 14.5, 14.4. Anal. Calcd. for $C_{104}H_{124}N_{12}K_2Ge_6$: C, 60.75; H, 6.08; N, 8.17. Found: C, 61.34; H, 5.98; N, 8.62.

Synthesis of $[K(THF)]_2Ge_6(\mu_3\text{-}\kappa^1\text{:}\kappa^1\text{:}\kappa^1\text{-DAP}^{Dipp})_2(\mu_4\text{-}\kappa^1\text{:}\kappa^1\text{:}\kappa^1\text{:}\eta^3\text{-DAP}^{Dipp})_2$ (11)

The mixture of **10** (0.124 g, 0.0942 mmol) and KC_8 (0.1138 g, 0.8419 mmol) was taken into a 20 mL of vial and 4 mL of THF were added at room temperature. The reaction mixture was allowed to stir for 4 hours to give an orange suspension. All volatiles were removed by *vacuo*. The residue was extracted with 2 mL of *n*-hexane for three times, and the insoluble material was filtered off through a pad of *Celite*. The orange filtrate was concentrated under vacuum to give an orange solid (0.0417 g, 0.0172 mmol, 36.5%). X-ray quality crystals of **11** were obtained from evaporation in a mixture of *n*-hexane and THF at room temperature. ^{1}H NMR (500 MHz, C_6D_6, 298K) δ 7.24-7.04 (m, 24H, 2,6-^{i}$Pr_2C_6\mathit{H}_3$), 5.32 (s, 4H, 4-$CH_3C_5\mathit{H}_2N$), 5.18 (s, 4H, 4-$CH_3C_5\mathit{H}_2N$), 3.65 (septet, 3H, *H*CMe_2), 3.40 (septet, 2H, *H*CMe_2), 3.33 (septet, 2H, *H*CMe_2), 3.13 (septet, 2H, *H*CMe_2), 3.02 (septet, 3H, *H*CMe_2), 2.96 (septet, 3H, *H*CMe_2), 1.47 (d, 12H, $CH\mathit{Me}_2$), 1.34 (s, 12H, 4-$C\mathit{H}_3C_5H_2N$), 1.29 (d, 12H, $CH\mathit{Me}_2$), 1.22 (d, 12H, $CH\mathit{Me}_2$), 1.19 (d, 12H, $CH\mathit{Me}_2$), 1.13 (d, 12H, $CH\mathit{Me}_2$), 1.09 (d, 12H, $CH\mathit{Me}_2$), 1.05 (d, 12H, $CH\mathit{Me}_2$), 0.77 (d, 12H, $CH\mathit{Me}_2$). ^{13}C{^{1}H} NMR (500 MHz, *d*$_8$-THF, 298K) δ 162.4, 160.9, 153.1, 147.9, 147.4, 147.1, 145.8, 143.1, 142.9, 127.3, 126.9, 125.9, 125.5, 125.0, 124.6, 123.6, 122.6, 101.8 ($\mathit{C}H_2$, *meta*-4-Me-pyridine), 101.2 ($\mathit{C}H_2$, *meta*-4-Me-pyridine), 30.2, 28.8, 28.4, 28.3, 26.5, 25.7, 24.5, 24.2, 23.9, 22.9, 21.0 ($\mathit{C}H_3$, 4-

Me-pyridine). Anal. Calcd. for $C_{120}H_{156}N_{12}Ge_6K_2$: C, 63.20; H, 6.89; N, 7.37. Found: C, 63.263; H, 7.173; N, 7.074.

Synthesis of [K(THF)]$_2$Ge$_2$(μ_4-κ^1:κ^1:κ^1:η^2-DAP^{Dipp})$_2$ (12)

12 was synthesized according to modified literature procedure.[3] Addition of 5 mL of THF into the reaction mixture of **1** (0.2754g, 0.2677 mmol) and KC_8 (0.1095 g, 0.8100 mmol) at room temperature resulting in the formation of yellow green suspension immediately. After stirring for 1 hour, volatiles were removed under vacuum, and 6 mL of the solution of *n*-hexane was added into the residue. The suspension was filtered through a pad of *Celite*. The filtrate was concentrated by *vacuo* to give an orange powder (0.2214 g, 0.1770 mmol, 66.1%). ^{1}H NMR (500 MHz, C_6D_6, 298K) δ 7.24 (d, 2H, *meta*-2,6-^{i}Pr$_2$C$_6$*H*$_3$), 7.20 (d, 2H, *meta*-2,6-^{i}Pr$_2$C$_6$*H*$_3$), 7.14 (d, 2H, *meta*-2,6-^{i}Pr$_2$C$_6$*H*$_3$), 7.07 (t, 2H, *para*-2,6-^{i}Pr$_2$C$_6$*H*$_3$), 6.81 (t, 2H, *para*-2,6-^{i}Pr$_2$C$_6$*H*$_3$), 6.73 (d, 2H, *meta*-2,6-^{i}Pr$_2$C$_6$*H*$_3$), 4.91 (s, 2H, 4-CH$_3$C$_5$*H*$_2$N), 4.51 (s, 2H, 4-CH$_3$C$_5$*H*$_2$N), 3.92 (septet, 2H, *H*CMe$_2$), 3.50 (septet, 2H, *H*CMe$_2$), 3.25 (septet, 2H, *H*CMe$_2$), 2.99 (septet, 2H, *H*CMe$_2$), 1.65 (s, 6H, 4-C*H*$_3$C$_5$H$_2$N), 1.35 (d, 6H, CH*Me*$_2$), 1.35 (d, 6H, CH*Me*$_2$), 1.34 (d, 6H, CH*Me*$_2$), 1.21 (d, 12H, CH*Me*$_2$), 1.10 (d, 6H, CH*Me*$_2$), 0.94 (d, 6H, CH*Me*$_2$), 0.81 (d, 6H, CH*Me*$_2$). ^{13}C{^{1}H} NMR (126 MHz, C_6D_6, 298K) δ 163.1, 162.3, 150.6, 150.0, 149.5, 148.2, 145.8, 144.2, 141.1, 125.4, 124.4, 124.1, 123.7, 123.3, 121.2, 96.3 (*C*H$_2$, *meta*-4-Me-pyridine), 90.8 (*C*H$_2$, *meta*-4-Me-pyridine), 28.6, 28.2, 28.1, 27.8, 27.4, 26.1, 25.8, 25.7, 24.6, 24.5, 23.5, 22.9, 21.2 (*C*H$_3$, 4-Me-pyridine). Anal. Calcd. for $C_{60}H_{78}N_6Ge_2K_2$: C, 65.11; H, 7.10; N, 7.59. Found: C, 65.55; H, 7.34; N, 7.28.

Synthesis of (SnCl)$_2$Ge$_2$(μ_3-κ^1:κ^1:κ^1-DAP^{Dipp})$_2$ (15)

A 20 mL of vial was charged with **12** (0.2314g, 0.2091 mmol) and $SnCl_2$ (0.0932 g, 0.4915 mmol), and 2 mL of THF was added as solvent. The reaction mixture was allowed to stir for 1 hour at room temperature to give a yellow suspension. The yellow suspension was concentrated under vacuum. The residue was extracted with 2 mL of *n*-hexane for three times and filtered through a pad of *Celite* to remove insoluble material. X-ray quality of **15** were obtained from slow evaporation in a mixture of hexane and Et_2O in 1:2 ratio at room temperature. The crystals were washed with *n*-hexane, and all

volatiles were removed by *vacuo* to give a yellow solid (0.0698g, 0.0522mmol, 24.9%). ^{1}H NMR (500 MHz, C_6D_6, 298K) δ 7.22-7.06 (m, 12H, 2,6-^{i}Pr$_2$C$_6$*H*$_3$), 5.25 (s, 2H, 4-CH$_3$C$_5$*H*$_2$N), 4.92 (s, 2H, 4-CH$_3$C$_5$*H*$_2$N), 3.55 and 3.49 (septet, 4H, *H*CMe$_2$), 3.52-3.47 (septet, 2H, *H*CMe$_2$), 3.07 (septet, 2H, *H*CMe$_2$), 2.35 (septet, 2H, *H*CMe$_2$), 1.40 (d, 6H, CH*Me*$_2$), 1.33 (d, 6H, CH*Me*$_2$), 1.31 (s, 6H, 4-C*H*$_3$C$_5$H$_2$N), 1.20 (d, 6H, CH*Me*$_2$), 1.17 (d, 6H, CH*Me*$_2$), 1.13 (d, 6H, CH*Me*$_2$), 1.07 (d, 6H, CH*Me*$_2$), 0.85 (d, 6H, CH*Me*$_2$), 0.79 (d, 6H, CH*Me*$_2$). ^{13}C{^{1}H} NMR (126 MHz, C_6D_6, 298K) δ 161.5, 161.4, 153.7, 147.5, 147.4, 145.8, 145.1, 141.7, 141.3, 129.5, 127.3, 126.1, 125.6, 125.2, 124.7, 100.9 (*C*H$_2$, *meta*-4-Me-pyridine), 97.3 (*C*H$_2$, *meta*-4-Me-pyridine), 30.2, 28.7, 28.1, 27.9, 27.4, 26.4, 26.1, 25.9, 24.8, 24.4, 24.1, 23.5, 20.8 (*C*H$_3$, 4-Me-pyridine). ^{119}Sn{^{1}H} NMR (187 MHz, C_6D_6, 298K) δ −245.26. Anal. Calcd. for $C_{60}H_{78}N_6Ge_2Sn_2Cl_2$: C, 53.91; H, 5.88; N, 6.29. Found: C, 54.30; H, 6.09; N, 6.20.

Synthesis of [K(Et$_2$O)]$_2$Ge$_4$Sn$_2$(μ_3-κ^1:κ^1:κ^1-DAP^{Dipp})$_2$(μ_4-κ^1:κ^1:κ^1:η^2-DAP^{Dipp})$_2$ (16)

The mixture of **15** (0.1641 g, 0.1227 mmol) and 0.5 wt% K/Hg (2.8876 g, 0.3694 mmol) was taken into a 20 mL of vial and 6 mL of the solution of Et$_2$O and THF in 2:1 ratio was added at room temperature. The reaction mixture was allowed to stir for 3 hours to give earthy yellow suspension. The crude material was collected after filtration, and all volatiles were removed by *vacuo*. The residue was extracted with 2 mL of *n*-hexane for three times, and the insoluble material was filtered off through a pad of *Celite*. The orange filtrate was concentrated under vacuum to give an earthy yellow solid (0.1128 g, 0.0447 mmol, 72.9%). X-ray quality crystals of **16** were obtained from slow evaporation in a mixture of *n*-hexane and Et$_2$O at room temperature. ^{1}H NMR (500 MHz, C_6D_6, 298K) δ 7.24-7.06 (m, 24H, 2,6-^{i}Pr$_2$C$_6$*H*$_3$), 5.30 (s, 2H, 4-CH$_3$C$_5$*H*$_2$N), 5.24 (s, 2H, 4-CH$_3$C$_5$*H*$_2$N), 5.22 (s, 2H, 4-CH$_3$C$_5$*H*$_2$N), 5.13 (s, 2H, 4-CH$_3$C$_5$*H*$_2$N), 3.54 (septet, 2H, *H*CMe$_2$), 3.37 (septet, 2H, *H*CMe$_2$), 3.07 (septet, 12H, *H*CMe$_2$), 1.45 (d, 12H, CH*Me*$_2$), 1.42 (d, 12H, CH*Me*$_2$), 1.34 (s, 12H, 4-C*H*$_3$C$_5$H$_2$N), 1.28 (d, 24H, CH*Me*$_2$), 1.16 (d, 12H, CH*Me*$_2$), 1.13 (d, 24H, CH*Me*$_2$), 0.77 (d, 12H, CH*Me*$_2$). ^{13}C{^{1}H} NMR (126 MHz, C_6D_6, 298K) δ 162.4, 160.9, 153.1, 147.9, 147.4, 147.1, 145.8, 143.1, 142.9, 127.3, 126.9, 125.9, 125.5, 125.0, 124.6, 123.6, 122.6, 101.8 (*C*H$_2$, *meta*-4-Me-pyridine), 101.2 (*C*H$_2$, *meta*-4-Me-pyridine), 30.2, 28.8, 28.4, 26.5, 25.7, 24.5, 24.2, 23.9, 22.9, 21.0 (*C*H$_3$, 4-Me-pyridine). ^{119}Sn{^{1}H} NMR (187 MHz, C_6D_6, 298K) δ −94.66. Anal. Calcd.

for $C_{120}H_{156}N_{12}Ge_4Sn_2K_2$: C, 60.74; H, 6.63; N, 7.08. Found: C, 61.35; H, 6.87; N, 7.08.

X-ray crystallography

Data collection of **2−6, 8**, **9**, **11** and **15−16** were carried out using the SMART program on a Bruker SMART Apex II diffractometer with CCD area detector and multi-layer mirror monochromated MoK*α* radiation (λ = 0.71073 Å) at 200(2) K or CuKα radiation (λ = 1.54184 Å) at 100(1) K. Cell parameters were retrieved and refined using *DENZO-SMN* software[6] on all observed reflections. Data reduction was performed with the *DENZO-SMN* software as well. An empirical absorption was based on the symmetry-equivalent reflections and applied the data using the *SORTAV* program.[7] Using *SHELXTL* program[8] on PC computer made the structure analysis. The structure was solved by using the *SHELXS-97* program and refined by using *SHELXL-97* program by full-matrix least squares on F^2 values.[9–11] All of non-hydrogen atoms are refined anisotropically. Hydrogen atoms attached to the carbons were fixed at calculated positions and refined using a riding mode. **10** was collected on Rigaku XtaLAB HyPix-Arc 150 diffractometer with Cu-K*α* radiation (λ = 1.54178 Å) at 100(10) K. The structure determinations and refinements were carried out using the *SHELXS*[10] and *SHELXL*[11] programs respectively on the Olex2 interface.[12] The structures were solved using direct methods, which yielded the positions of all nonhydrogen atoms. Hydrogen atoms were placed in calculated positions in the final structure refinement. Crystallographic refinement parameters are listed in Table *S1*−Table *S3*.

2. Details of crystal data

Table *S*1. Crystal and intensity collection data for complexes **2**–**5**.

Complex	**2**	**3**	**4**	**5**
Formula	$C_{55}H_{62}N_6Ge_2$	$C_{52}H_{62}N_6Ge_2$	$C_{48}H_{54}N_6Ge_3$	$C_{52}H_{62}N_6Ge_3$
Crystal system	Monoclinic			
Formula weight	952.29	916.26	932.74	988.85
Space group	P 2/n	P 21/c	P 21/c	P 21/c
a, Å	21.0754(9)	17.4841(8)	14.2780(3)	17.3780(3)
b, Å	8.5083(3)	16.8732(7)	19.8325(4)	16.7069(3)
c, Å	27.4464(15)	16.5793(6)	15.7105(3)	17.1615(4)
α, deg	90			
β, deg	95.243(2)	103.2130(10)	93.6360(10)	104.1120(10)
γ, deg	90			
V, Å^3	4901.0(4)	4761.6(3)	4439.76(15)	4832.17(16)
Z	4			
Cryst dimens, mm^3	0.65 x 0.13 x 0.08	0.31 x 0.24 x 0.05	0.31 x 0.20 x 0.11	0.40 x 0.22 x 0.06
Kα radiation: γ, Å	0.71073			
θrang, deg	2.34 to 25.15	2.39 to 25.05	2.13 to 25.05	2.42 to 25.04
Limiting indices	-25<=h<=25<br>-10<=k<=10<br>-32<=l<=32	-20<=h<=20<br>-19<=k<=20<br>-19<=l<=18	-17<=h<=16<br>-23<=k<=23<br>-17<=l<=18	-20<=h<=20<br>-19<=k<=19<br>-20<=l<=20
Reflections collected	79158	66461	50616	68499
Independent reflections	8716 [R_{int} = 0.0594]	8396 [R_{int} = 0.0673]	7809 [R_{int} = 0.0383]	8506 [R_{int} = 0.0508]
Max. and min. transmission	0.9053 and 0.4926	0.9377 and 0.6881	0.8053 and 0.5679	0.8948 and 0.5178
Refinement method	Full-matrix least-squares on F^2			
Data / restraints / parameters	8716 / 0 / 583	8396 / 12 / 535	7809 / 0 / 514	8506 / 0 / 534
GOF	1.046	1.071	1.055	1.057
Final R indices [I>2σ(I)]	R_1 = 0.0314, wR_2 = 0.0712	R_1 = 0.0819, wR_2 = 0.2038	R_1 = 0.0329, wR_2 = 0.0784	R_1 = 0.0339, wR_2 = 0.0757
R indices (all data)	R_1 = 0.0501, wR_2 = 0.0828	R_1 = 0.1152, wR_2 = 0.2332	R_1 = 0.0467, wR_2 = 0.0893	R_1 = 0.0542, wR_2 = 0.0879
Largest diff. peak and hole	0.353 and -0.375 e.Å^{-3}	2.833 and -0.896 e.Å^{-3}	0.535 and -0.491 e.Å^{-3}	0.918 and -0.789 e.Å^{-3}

Table *S*2. Crystal and intensity collection data for complexes **(6•Et$_2$O), (6•5C$_6$D$_6$), 8** and **9**.

Complex	**(6•Et$_2$O)**	**(6•5C$_6$D$_6$)**	**8**	**9**
Formula	$C_{56}H_{72}N_6Ge_3InCl_3O$	$C_{82}H_{92}N_6Ge_3InCl_3$	$C_{52}H_{62}N_6Ge_4Cl_4$	$C_{60}H_{78}N_6Ge_4Cl_4$
Crystal system	Monoclinic	Triclinic	Monoclinic	Orthorhombic
Formula weight	1284.14	1600.56	1203.24	1315.44
Space group	P 21/n	P -1	C 2/c	P b c n
a, Å	12.9022(4)	15.017(2)	20.5412(5)	20.7840(12)
b, Å	26.1432(6)	15.127(2)	18.2801(4)	16.2199(10)
c, Å	19.6103(5)	17.601(2)	14.7639(4)	19.0520(11)
α, deg	90	85.624(4)	90	90
β, deg	94.4390(10)	75.700(4)	103.3840(10)	90
γ, deg	90	86.096(5)	90	90
V, Å^3	6594.8(3)	3858.1(9)	5393.2(2)	6422.7(7)
Z	4	2	4	4
Cryst dimens, mm^3	0.74 x 0.16 x 0.11	0.75 x 0.58 x 0.55	0.45 x 0.41 x 0.28	0.26 x 0.24 x 0.09
Kα radiation: γ, Å	0.71073			
θrang, deg	1.99 to 25.03	2.05 to 25.13	2.84 to 25.03	1.59 to 25.02
Limiting indices	-15<=h<=15<br>-31<=k<=30<br>-23<=l<=23	-17<=h<=17<br>-18<=k<=18<br>-20<=l<=20	-24<=h<=24<br>-21<=k<=21<br>-17<=l<=17	-22<=h<=24<br>-18<=k<=19<br>-22<=l<=22
Reflections collected	128165	140197	47571	38500
Independent reflections	11639 [R_{int} = 0.0808]	13651 [R_{int} = 0.0480]	4704 [R_{int} = 0.0363]	5655 [R_{int} = 0.1080]
Max. and min. transmission	0.8217 and 0.3401	0.4726 and 0.3794	0.5475 and 0.4057	0.8362 and 0.6164
Refinement method	Full-matrix least-squares on F^2			
Data / restraints / parameters	11639 / 1 / 625	13651 / 0 / 817	4704 / 7 / 277	5655 / 0 / 334
GOF	0.764	1.009	1.034	1.021
Final R indices [I > 2σ(I)]	R_1 = 0.0358,<br>wR_2 = 0.0934	R_1 = 0.0289,<br>wR_2 = 0.0743	R_1 = 0.0340,<br>wR_2 = 0.0803	R_1 = 0.0481,<br>wR_2 = 0.1024
R indices (all data)	R_1 = 0.0461,<br>wR_2 = 0.1011	R_1 = 0.0342,<br>wR_2 = 0.0789	R_1 = 0.0387,<br>wR_2 = 0.0842	R_1 = 0.0991,<br>wR_2 = 0.1252
Largest diff. peak and hole	1.013 and -0.938 e.Å^{-3}	1.260 and -0.708 e.Å^{-3}	0.685 and -0.654 e.Å^{-3}	0.638 and -0.683 e.Å^{-3}

Table *S*3. Crystal and intensity collection data for complexes **10**, **11**, **15** and **16**.

Complex	**10**	**11**	**15**	**16**
Formula	$C_{59}H_{70}Ge_3KN_6$	$C_{128}H_{172}N_{12}O_2Ge_6K_2$	$C_{60}H_{78}Cl_2Ge_2N_6Sn_2$	$C_{136}H_{196}N_{12}O_4Ge_4Sn_2K_2$
Crystal system	triclinic	Triclinic	Monoclinic	Monoclinic
Formula weight	1120.08	2424.52	1336.74	2668.99
Space group	P-1	P-1	P 21/n	P 21/c
a, Å	14.0678(4)	13.4215(3)	10.1866(4)	14.4926(5)
b, Å	14.7790(5)	14.4964(3)	45.1350(18)	16.8204(6)
c, Å	15.5226(5)	19.3299(5)	15.4786(6)	28.9299(8)
α, deg	69.128(3)	79.1680(10)	90	90
β, deg	88.246(2)	77.8590(10)	97.208(2)	102.3110(10)
γ, deg	79.767(3)	72.4930(10)	90	90
V, $Å^3$	2965.65(17)	3474.98(14)	7060.4(5)	6890.1(4)
Z	2	1	4	2
Cryst dimens, mm^3	0.11 × 0.09 × 0.08	0.25 x 0.17 x 0.04	0.32 x 0.26 x 0.06	0.24 x 0.15 x 0.06
Kα radiation: γ, Å	1.54184	0.71073	0.71073	0.71073
θrang, deg	4.133 to 72.927	2.47 to 25.07	1.40 to 25.03	2.12 to 25.04
Limiting indices	$-14 \leq h \leq 17$<br>$-17 \leq k \leq 17$<br>$-18 \leq l \leq 19$	-16<=h<=15<br>-17<=k<=17<br>-23<=l<=22	-12<=h<=12<br>-53<=k<=33<br>-18<=l<=17	-16<=h<=17<br>-20<=k<=20<br>-34<=l<=34
Reflections collected	37891	83850	46281	103385
Independent reflections	11142 [R_{int} = 0.0234]	12211 [R_{int} = 0.0406]	12376 [R_{int} = 0.0521]	12166 [R_{int} = 0.0726]
Max. and min. transmission	1.0000 and 0.8520	0.9465 and 0.7228	0.9073 and 0.6197	0.9243 and 0.7405
Refinement method	Full-matrix least-squares on F^2			
Data / restraints / parameters	11142/58/686	12211 / 0 / 668	12376 / 0 / 649	12166 / 2 / 719
GOF	1.050	1.008	0.592	1.136
Final R indices [I>2σ(I)]	R_1 = 0.0359, wR_2 = 0.0956	R_1 = 0.0352, wR_2 = 0.1016	R_1 = 0.0402, wR_2 = 0.1134	R_1 = 0.0636, wR_2 = 0.1639
R indices (all data)	R_1 = 0.0447, wR_2 = 0.0994	R_1 = 0.0444, wR_2 = 0.1082	R_1 = 0.0587, wR_2 = 0.1300	R_1 = 0.1015, wR_2 = 0.2036
Largest diff. peak and hole	1.26 and -0.59 e $Å^{-3}$	0.924 and -0.480 e.$Å^{-3}$	1.010 and -0.835 e.$Å^{-3}$	2.554 and -1.624 e.$Å^{-3}$

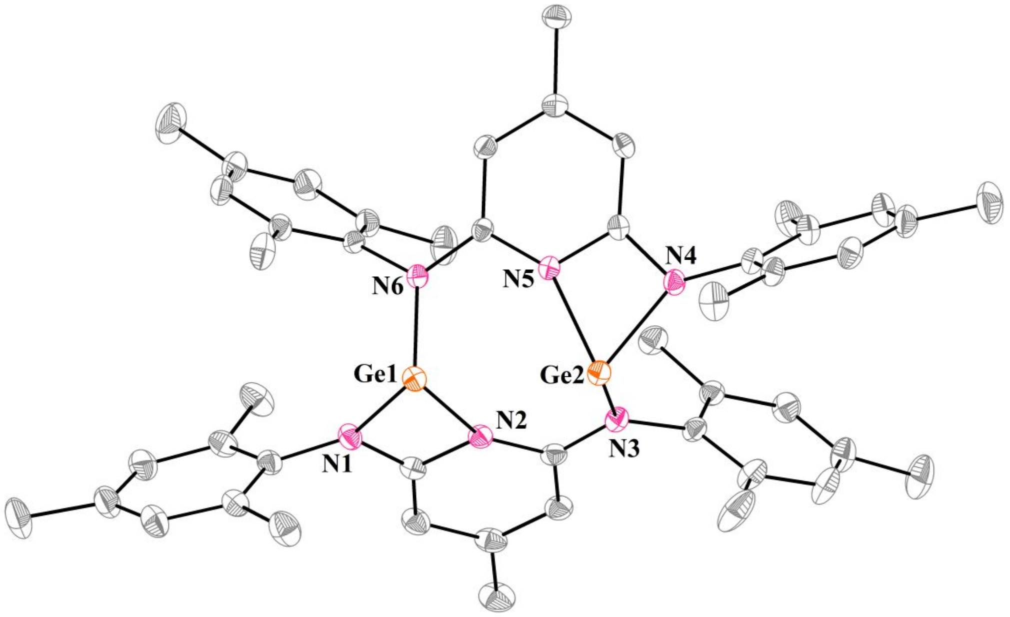

Figure *S*1. The solid-state molecular structure of **2** with thermal ellipsoids at 30% probability. The hydrogen atoms and Toluene solvate have been omitted for clarity. Selected bond lengths (Å) and angles (˚): Ge1−N1, 1.969(2); Ge1−N2, 2.0788(19); Ge1−N6, 1.954(2); Ge2−N3, 1.964(2); Ge2−N4, 1.985(2); Ge2−N5, 2.0828(19); N1−Ge1−N6, 97.48(9); N2−Ge1−N6, 93.58(8).

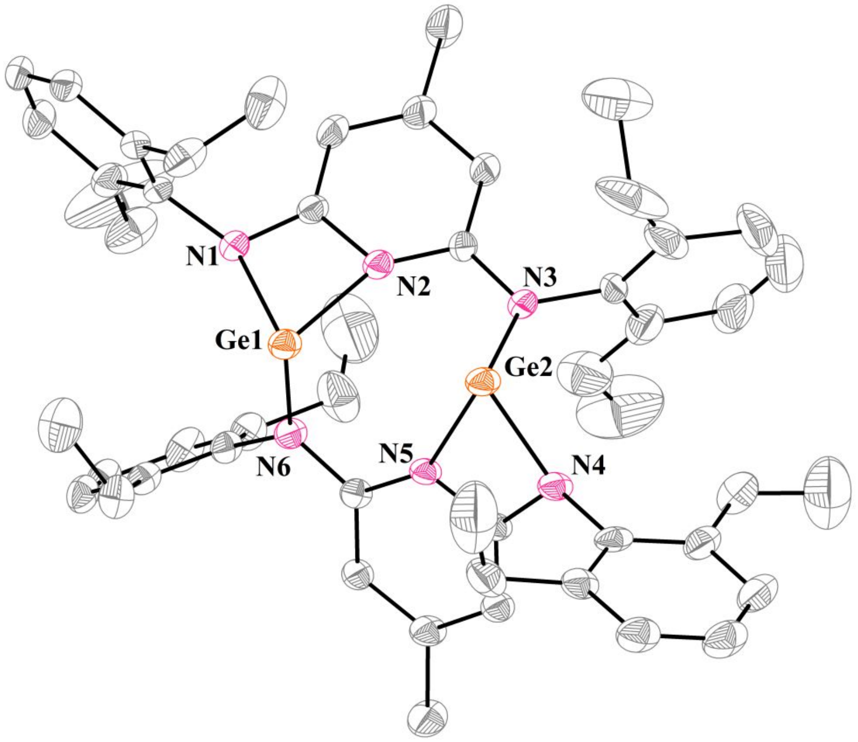

Figure *S*2. The solid-state molecular structure of **3** with thermal ellipsoids at 30% probability. The hydrogen atoms have been omitted for clarity. Selected bond lengths (Å) and angles (˚): Ge1−N1, 1.976(5); Ge1−N2, 2.084(6); Ge1−N6, 1.965(6); Ge2−N3, 1.956(6); Ge2−N4, 1.970(6); Ge2−N5, 2.091(5); N3−Ge2−N4, 100.5(3); N3−Ge2−N5, 93.6(2).

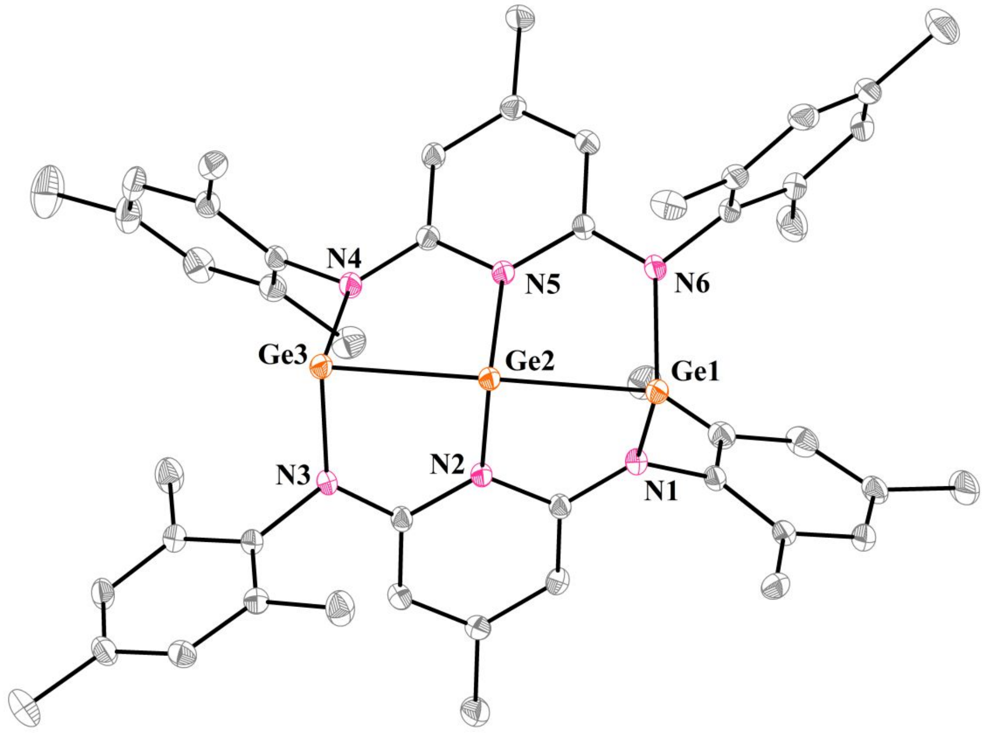

Figure *S*3. The solid-state molecular structure of **4** with thermal ellipsoids at 30% probability. The hydrogen atoms have been omitted for clarity. Selected bond lengths (Å) and angles (˚) :Ge1−Ge2, 2.5060(4); Ge2−Ge3, 2.5106(4); Ge1−N1, 2.031(3); Ge1−N6, 1.981(3); Ge2−N2, 1.971(2); Ge2−N5, 1.973(3); Ge3−N3, 1.960(3); Ge3−N4, 2.021(3), Ge1−Ge2−Ge3, 177.826(18); N1−Ge1−N6, 99.62(11); N2−Ge2−N5, 100.42(10); N3−Ge3−N4 98.82(11); N6−Ge1−Ge2, 83.94(7); N1−Ge1−Ge2, 80.84(7); Ge2−Ge3−N3,84.31(7); Ge2−Ge3−N4, 80.96(7).

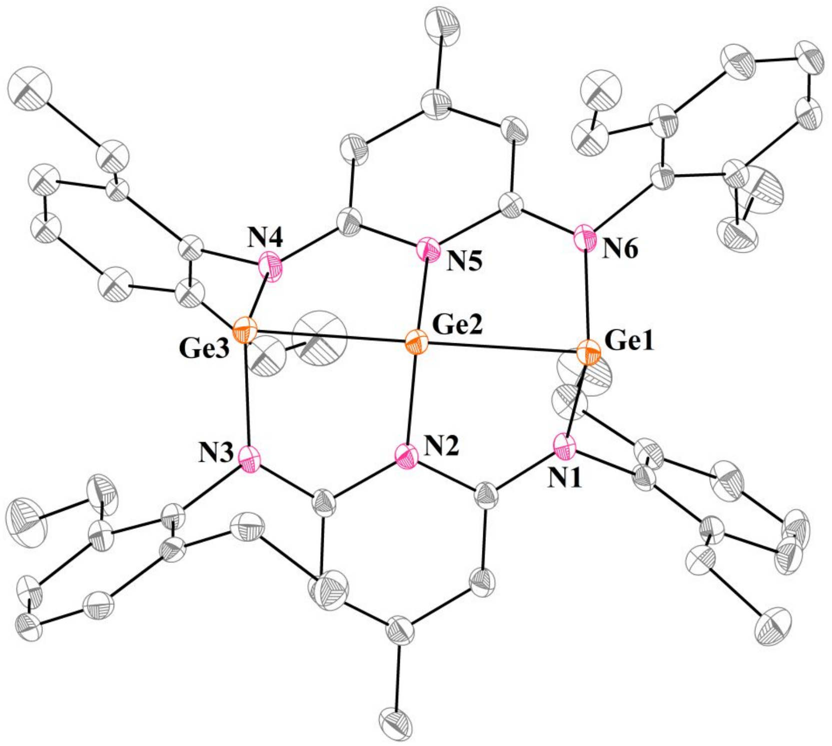

Figure *S*4. The solid-state molecular structure of **5** with thermal ellipsoids at 30% probability. The hydrogen atoms have been omitted for clarity. Selected bond lengths (Å) and angles (˚) Ge1−Ge2, 2.5042(4); Ge2−Ge3, 2.4986(4); Ge1−N1, 2.023(2); Ge1−N6, 1.965(2); Ge2−N2, 1.968(2); Ge2−N5, 1.965(2); Ge3−N3, 1.966(3); Ge3−N4, 2.026(3); Ge1−Ge2−Ge3, 176.609(19); N1−Ge1−N6, 100.11(10); N2−Ge2−N5, 101.00(10); N3−Ge3−N4, 99.37(11); Ge2−Ge1−N1, 80.61(7); Ge2−Ge1−N6, 84.14(7); N3−Ge3−Ge2, 84.07(7); N4−Ge3−Ge2, 80.65(7).

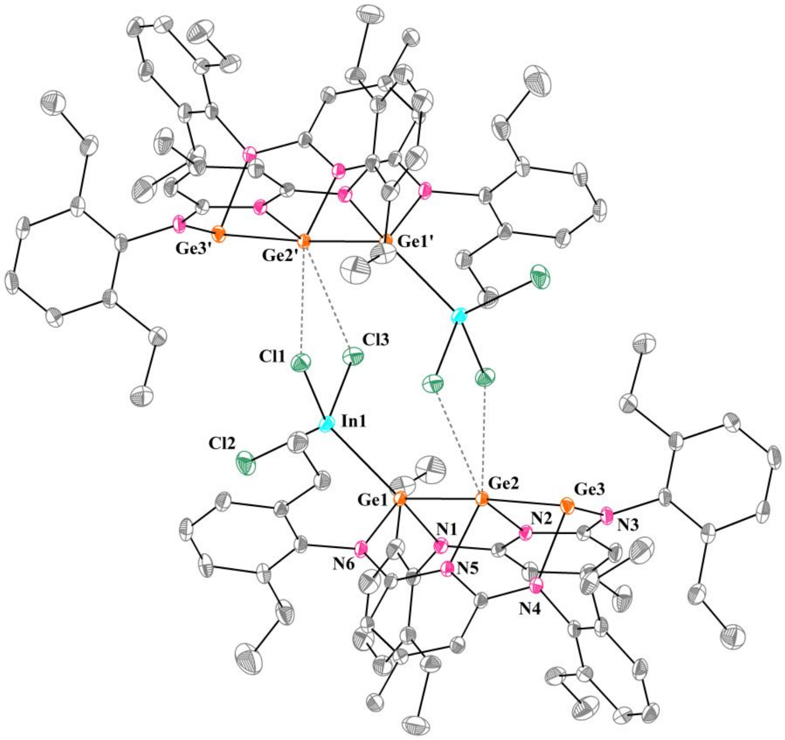

Figure *S*5. The solid-state molecular structure of **6□5C_6D_6** with thermal ellipsoids at 30% probability. The hydrogen atoms and C_6H_6 have been omitted for clarity. Selected bond lengths (Å) and angles (˚): Ge1−Ge2, 2.4047(4); Ge2−Ge3, 2.5212(4); In1−Ge1, 2.6383(4); In1−Cl1, 2.3993(7); In1−Cl2, 2.3608(7); In1−Cl3, 2.3842(8); Ge1−N1, 1.9290(19); Ge1−N6, 1.897(2);Ge2−N2, 1.9820(19); Ge2−N5, 2.003(2); Ge3−N3, 1.961(2); Ge3−N4, 2.033(2); Ge2'···Cl1, 3.4724(8); Ge2'···Cl3, 3.3373(8); Ge1−Ge2−Ge3, 176.260(13); In1−Ge1−Ge2, 137.140(13); N1−Ge1−N6, 105.16(9); N2−Ge2−N5, 99.02(8); N3−Ge3−N4, 97.59(9); Ge2−Ge3−N3, 83.05(6); Ge2−Ge3−N4, 79.47(6).

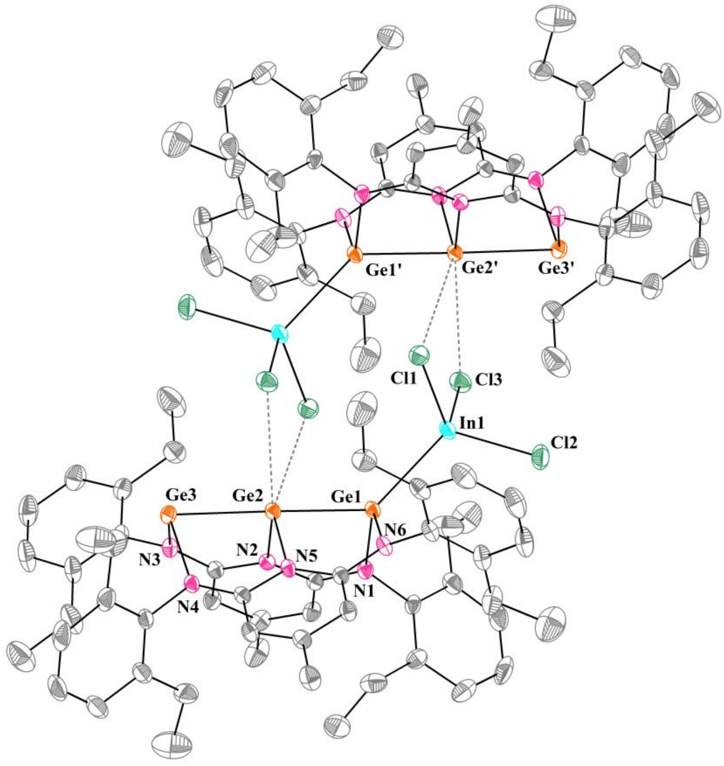

Figure *S*6. The solid-state molecular structure of **6□Et$_2$O** with thermal ellipsoids at 30% probability. The hydrogen atoms and Et$_2$O have been omitted for clarity. Selected bond lengths (Å) and angles (˚): Ge1−Ge2, 2.3987(5); Ge2−Ge3, 2.5031(5); In1−Ge1, 2.6217(4); In1−Cl1, 2.4018(9); In1−Cl2, 2.3580(10); In1−Cl3, 2.3805(10); Ge1−N1, 1.915(3); Ge1−N6, 1.893(3);Ge2−N2, 1.976(3); Ge2−N5, 1.989(3); Ge2'···Cl1, 3.4091(10); Ge2'···Cl3, 3.3514(11); Ge3−N3, 1.973(3); Ge3−N4, 2.043(3); Ge1−Ge2−Ge3, 178.77(2); In1−Ge1−Ge2, 134.405(18); N1−Ge1−N6, 104.69(12); N2−Ge2−N5, 98.92(11); N3−Ge3−N4, 99.18(11); Ge2−Ge3−N3, 83.40(8); Ge2−Ge3−N4, 79.60(7).

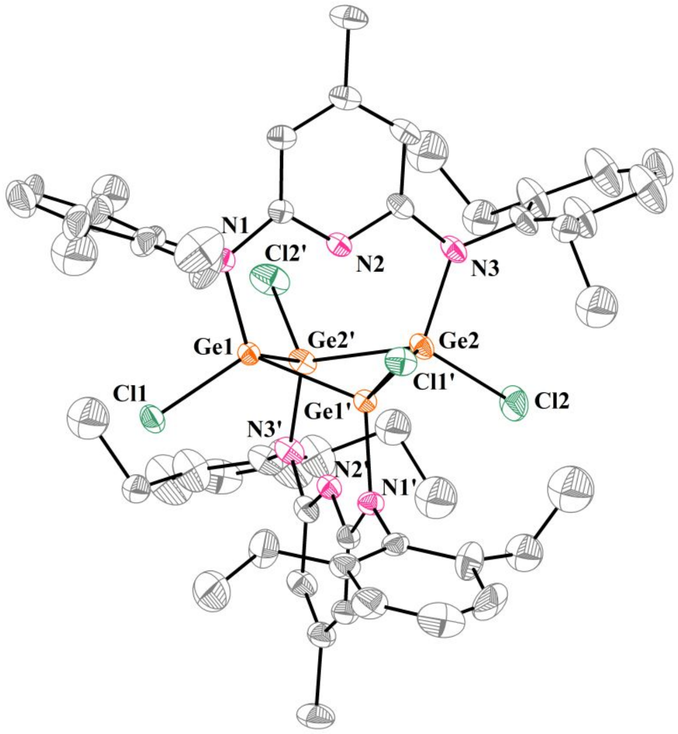

Figure *S*7. The solid-state molecular structure of **8** with thermal ellipsoids at 30% probability. The hydrogen atoms have been omitted for clarity. Selected bond lengths (Å) and angles (˚): Ge1−Ge1', 2.4354(6); Ge1−Ge2', 2.4460(4); Ge2−Ge2', 2.4438(7); Ge1'−Ge2, 2.4460(4); Ge1−N1, 1.867(3); Ge2−N3, 1.870(3); Ge1−Cl1, 2.1567(8); Ge2−Cl2, 2.1466(10); Ge1'−Ge1−Ge2', 86.787(11); Ge1'−Ge2−Ge2', 86.603(11).

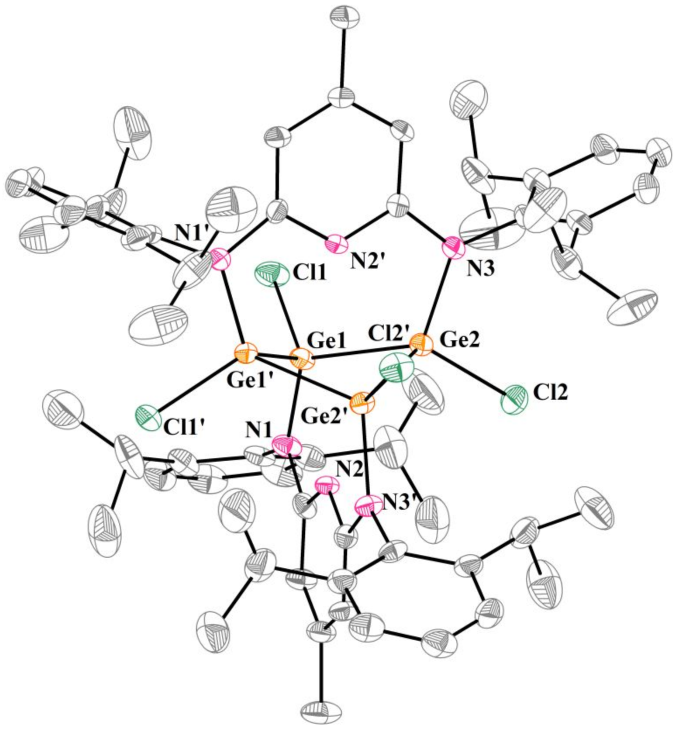

Figure *S*8. The solid-state molecular structure of **9** with thermal ellipsoids at 30% probability. The hydrogen atoms have been omitted for clarity. Selected bond lengths (Å) and angles (˚): Ge1−Ge2, 2.4677(7); Ge1−Ge1', 2.4442(10); Ge2−Ge2', 2.4676(10); Ge1'−Ge2', 2.4677(7); Ge2−Cl2, 2.1507(13); Ge1−Cl1, 2.1581(14); Ge2−N3, 1.874(4); Ge1−N1, 1.867(4); Ge1−Ge2−Ge2', 86.293(18); Ge1'−Ge1−Ge2, 86.805(16).

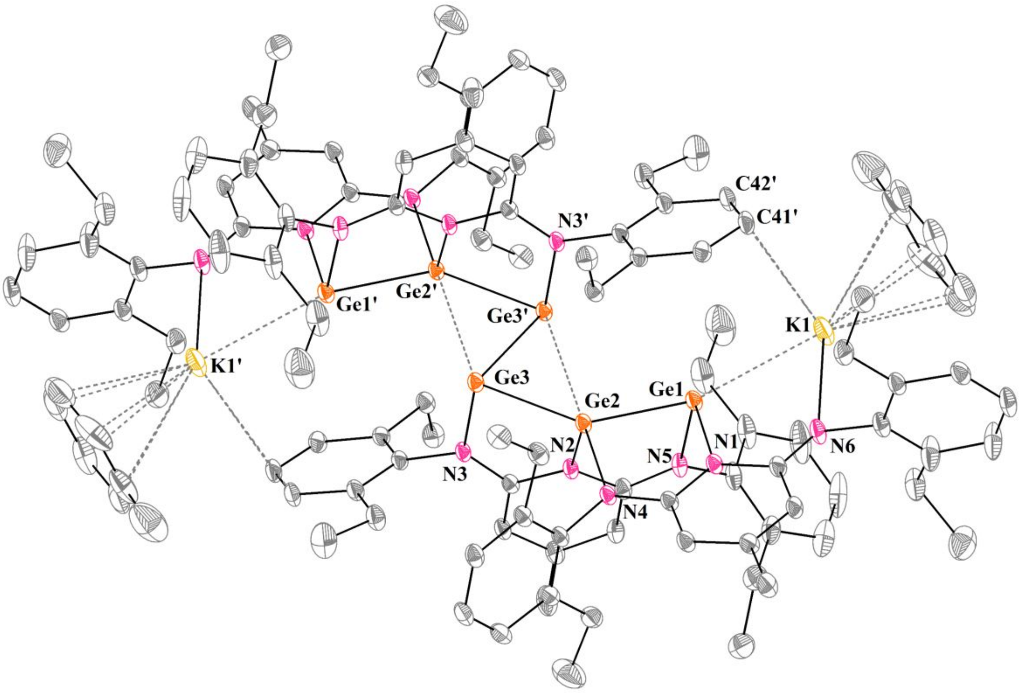

Figure *S*9. The solid-state molecular structure of **10** with thermal ellipsoids at 30% probability. The hydrogen atoms have been omitted for clarity. Selected bond lengths (Å) and angles (˚): Ge1−Ge2, 2.4398(4); Ge2−Ge3, 2.4421(4); Ge3−Ge3', 2.7216(6); Ge2'□□□ Ge3, 3.1328(4); Ge1□□□K1, 3.1681(12); K1□□□C41', 3.397(4); K1□□□C42', 3.518(4); Ge3−N3, 2.035(2); Ge1−N2, 2.024(2); Ge1−N4, 2.004(2); Ge2−N4, 1.954(2); Ge2−N2, 1.968(2); K1−N6, 2.734(3), Ge1−Ge2−Ge3, 144.332(15), Ge2−Ge3−Ge3', 74.479(14), Ge3'−Ge3−N3, 91.95(6), N2−Ge2−N4, 98.38(9), N1−Ge1−N5, 95.84(9), Ge1−K1−N1, 59.08(5).

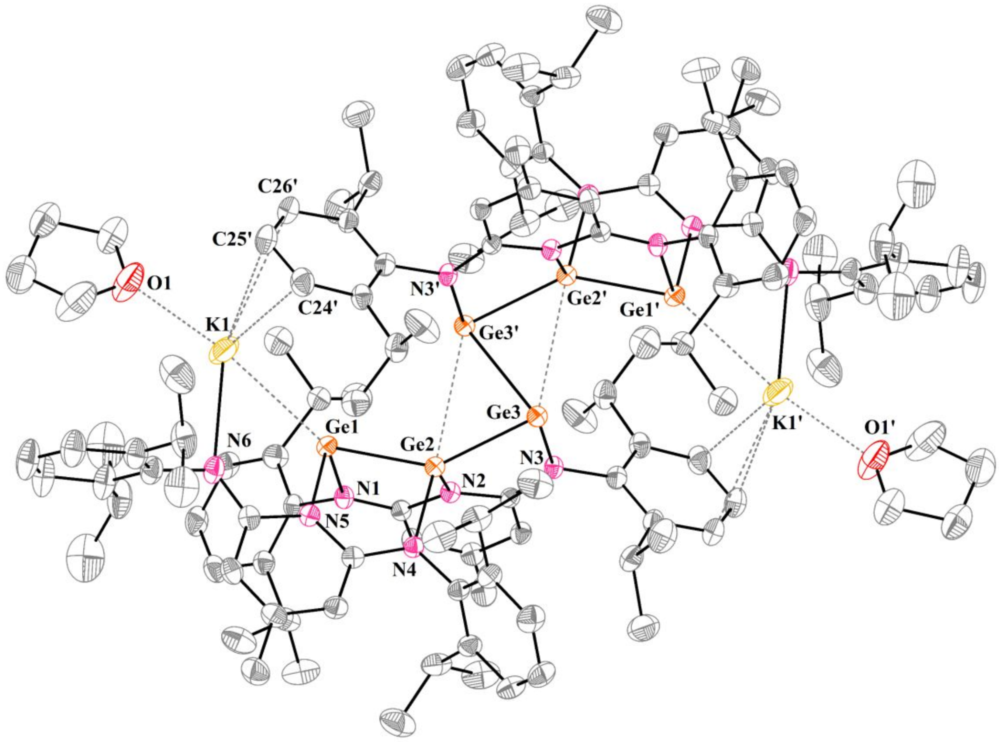

Figure *S*10. The solid-state molecular structure of **11** with thermal ellipsoids at 30% probability. The hydrogen atoms have been omitted for clarity. Selected bond lengths (Å) and angles (˚): Ge1−Ge2, 2.4446(4); Ge2−Ge3, 2.4734(4); Ge3−Ge3', 2.7634(5); Ge2'□□□ Ge3, 3.2895(3); Ge1□□□K1, 3.1870(8); K1□□□C24', 3.233(4); K1□□□C25', 3.063(4); K1□□□C26', 3.388(4); K1-O1, 2.563(3); Ge2−N2, 1.9671(19); Ge2−N4, 1.960(2); Ge1−N1, 1.993(2); Ge1−N5, 2.016(2); Ge3−N3, 2.059(2); K1−N6, 2.683(3); Ge2−Ge3−Ge3', 77.609(12); Ge1−Ge2−Ge3, 141.969(14); Ge2−Ge1−K1, 136.68(2); Ge1−K1−O1, 155.82(9); N3−Ge3−Ge3', 98.34(6); N1−Ge1−N5, 98.27(8); N2−Ge2−N4, 99.46(9).

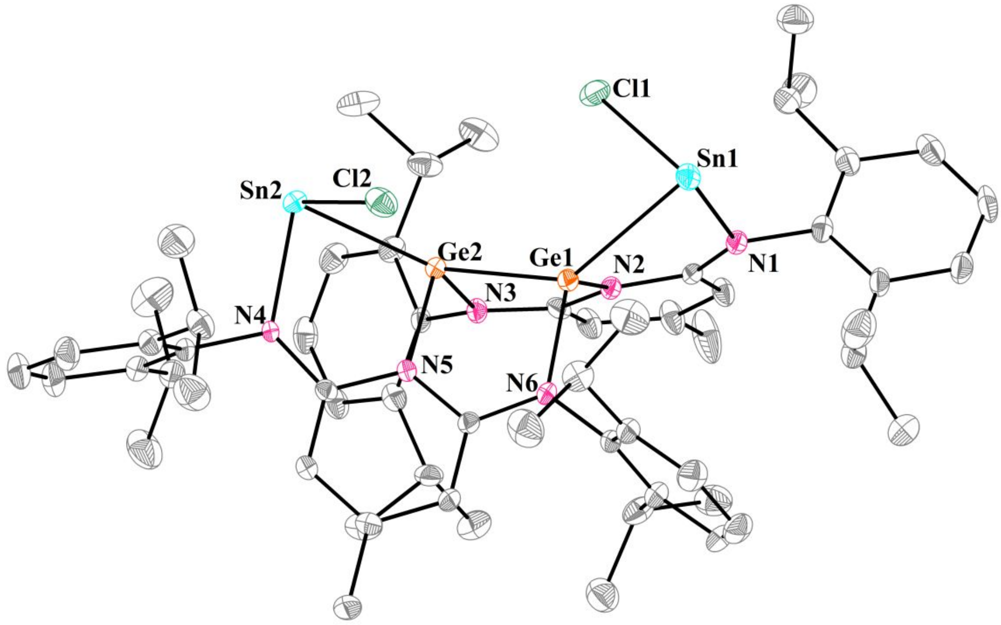

Figure *S*11. The solid-state molecular structure of **15** with thermal ellipsoids at 30% probability. The hydrogen atoms have been omitted for clarity. Selected bond lengths (Å) and angles (˚): Ge1−Ge2, 2.3735(6); Sn1−Ge1, 2.6807(6); Sn2−Ge2, 2.6807(5); Sn1−Cl1, 2.4919(13); Sn2−Cl2, 2.4989(15); Ge1−N2, 1.989(4); Ge1−N6, 1.927(4); Ge2−N3, 1.925(4); Ge2−N5, 1.987(4); Sn1−N1, 2.170(4); Sn2−N4, 2.173(4); Sn1−Ge1−Ge2, 135.52(2); Sn2−Ge2−Ge1, 135.35(2), Cl1−Sn1−N1, 93.47(10); Ge1−Sn1−N1, 76.91(10); Cl2−Sn2− N4, 93.15(11); Ge2−Sn2−N4, 77.24(9).

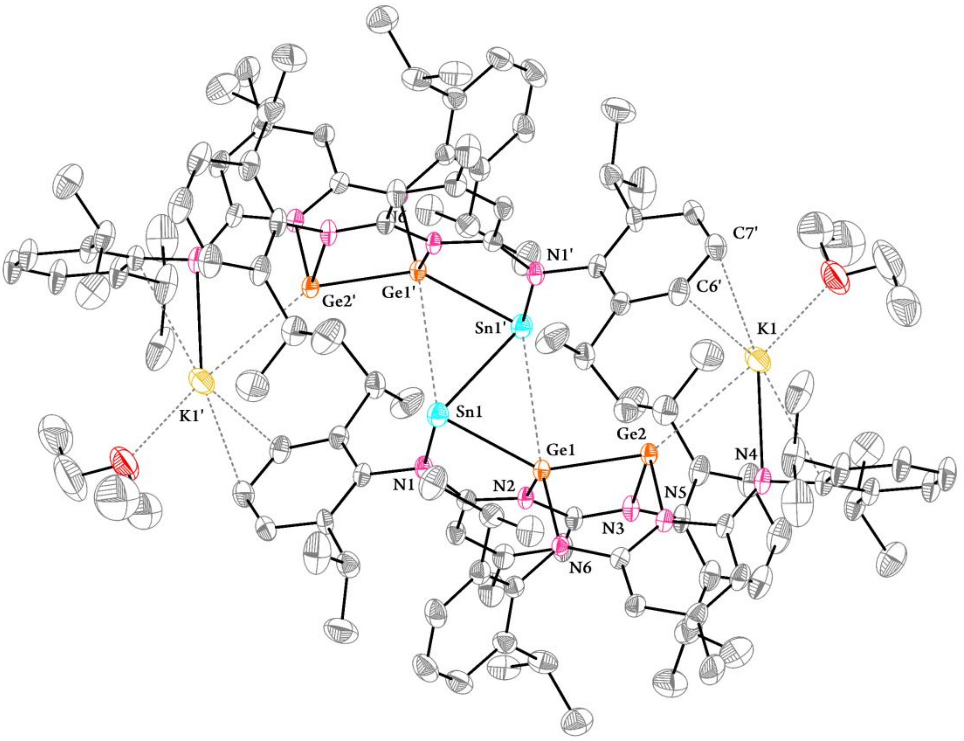

Figure *S*12. The solid-state molecular structure of **16** with thermal ellipsoids at 30% probability. The hydrogen atoms have been omitted for clarity. Selected bond lengths (Å) and angles (˚): Sn1−Sn1', 2.9718(9); Sn1−Ge1, 2.5865(7); Ge1−Ge2, 2.4473(8); Sn1□□□Ge1', 3.3944(8); Ge2□□□K1, 3.2605(18); Sn1−N1, 2.195(5); 2.021(5); K1□□□C7', 3.194(8); K1□□□C6', 3.285(7), K1−O1, 2.669(6); K1−N4, 2.729(6); K1−O1 2.669(6); Ge1−N2, 1.984(4); Ge1−N6, 1.953(5); Ge2−N3, 1.996(5); Ge2−N5, Sn1'−Sn1−Ge1, 74.93(2); Sn1−Ge1−Ge2, 142.21(3); Ge1−Ge2−K1, 137.51(4); Sn1'−Sn1−N1, 93.71(13); Ge1−Sn1−N1, 80.38(12); N2−Ge1−N6, 103.7(2); N3−Ge2−N5, 97.0(2).

3. NMR spectra

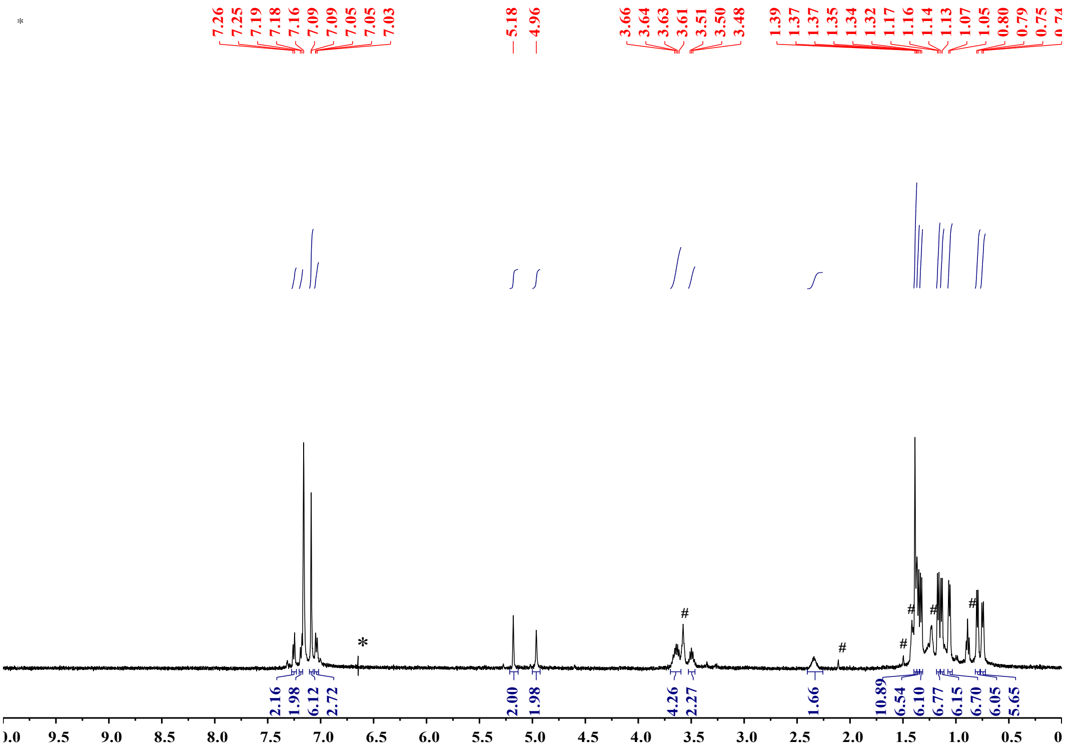

Figure *S*13. ^{1}H NMR spectrum of **1** (500 MHz, C_6D_6, 298K). (*: ghost peak; #: Hexane, Et_2O, Toluene and THF)

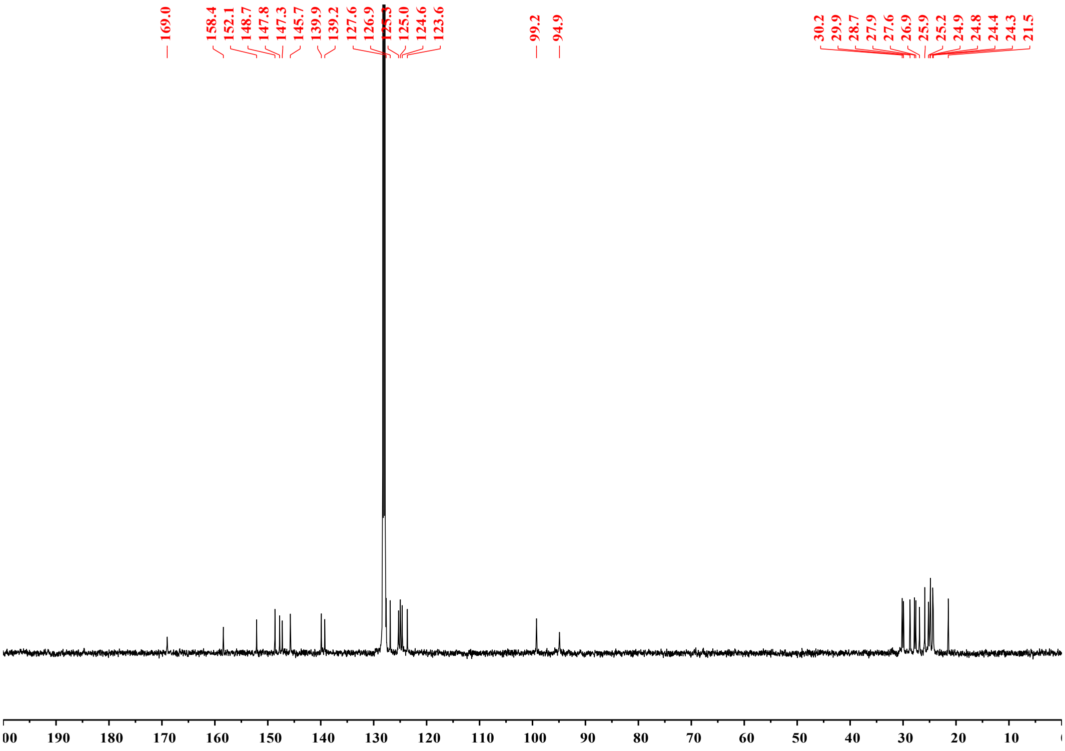

Figure *S*14. $^{13}C\{^{1}H\}$ NMR spectrum of **1** (126 MHz, C_6D_6, 298K).

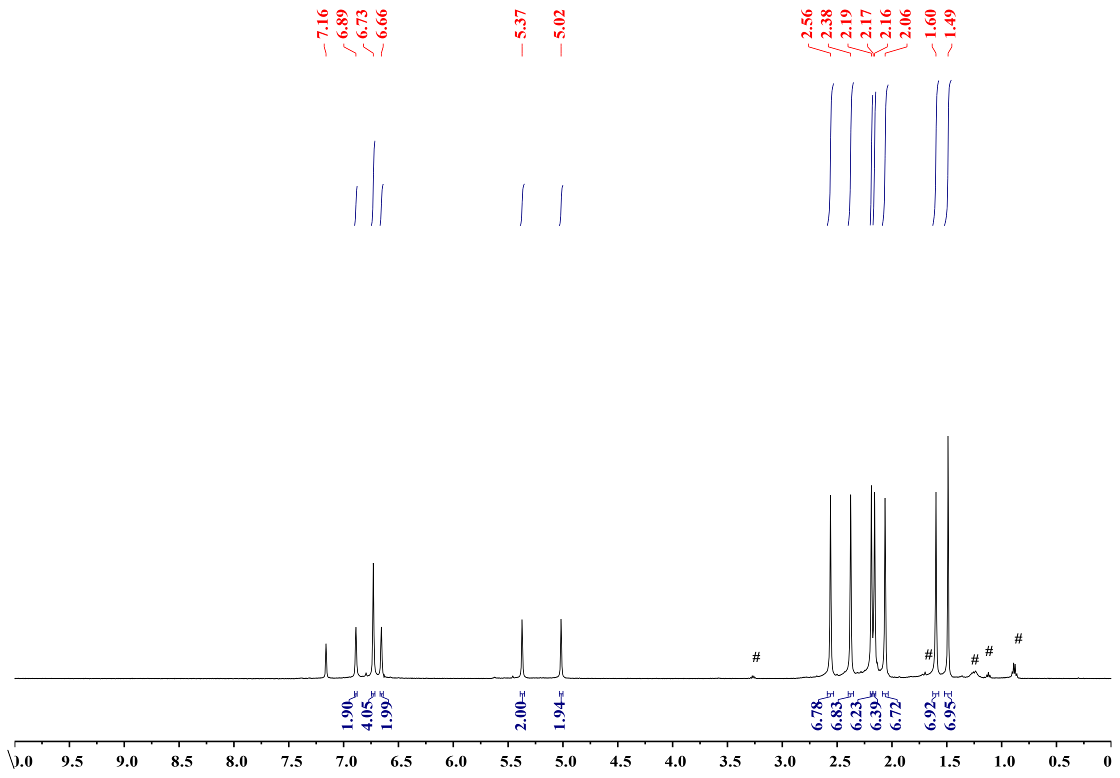

Figure *S*15. ^{1}H NMR spectrum of **2** (500 MHz, C_6D_6, 298K). ($^{\#}$: Hexane and Et_2O)

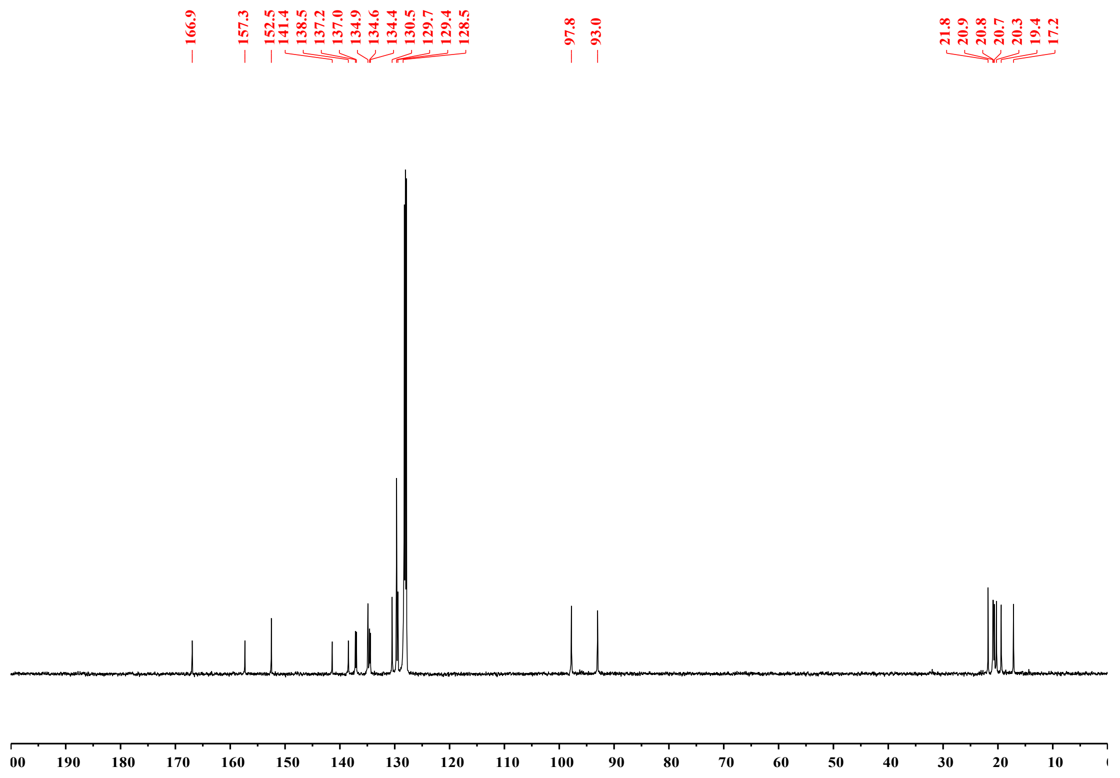

Figure *S*16. $^{13}C\{^{1}H\}$ NMR spectrum of **2** (126 MHz, C_6D_6, 298K).

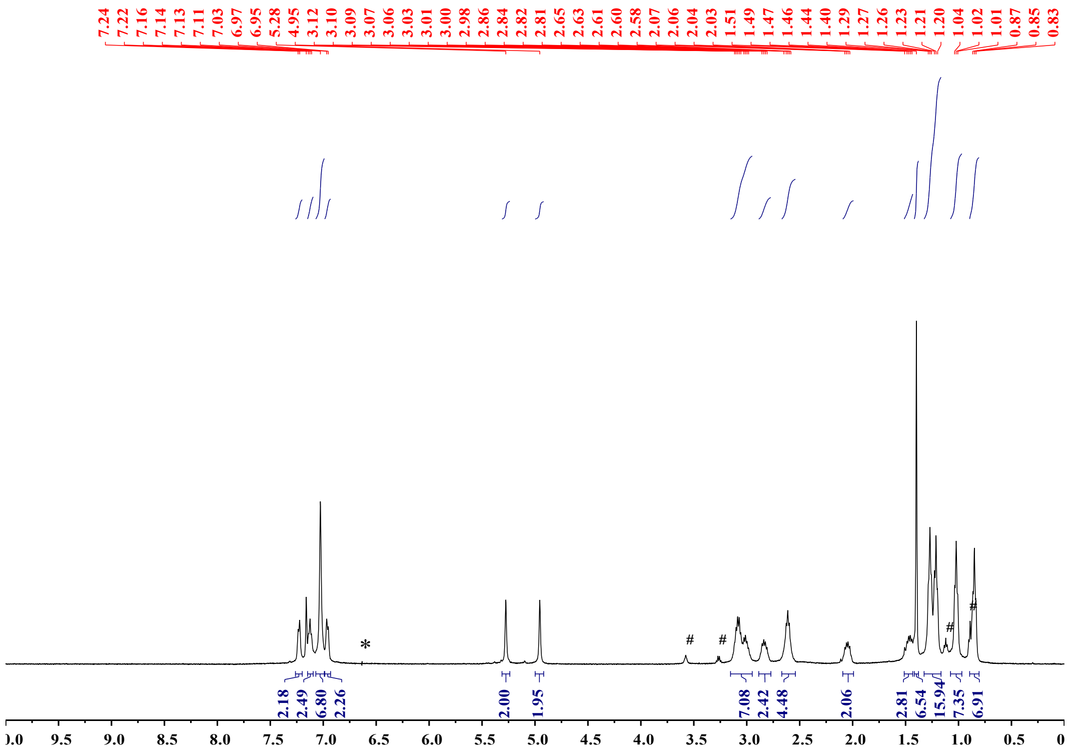

Figure *S*17. ^{1}H NMR spectrum of **3** (500 MHz, C_6D_6, 298K). (*: ghost peak; #: Hexane, Et_2O and THF)

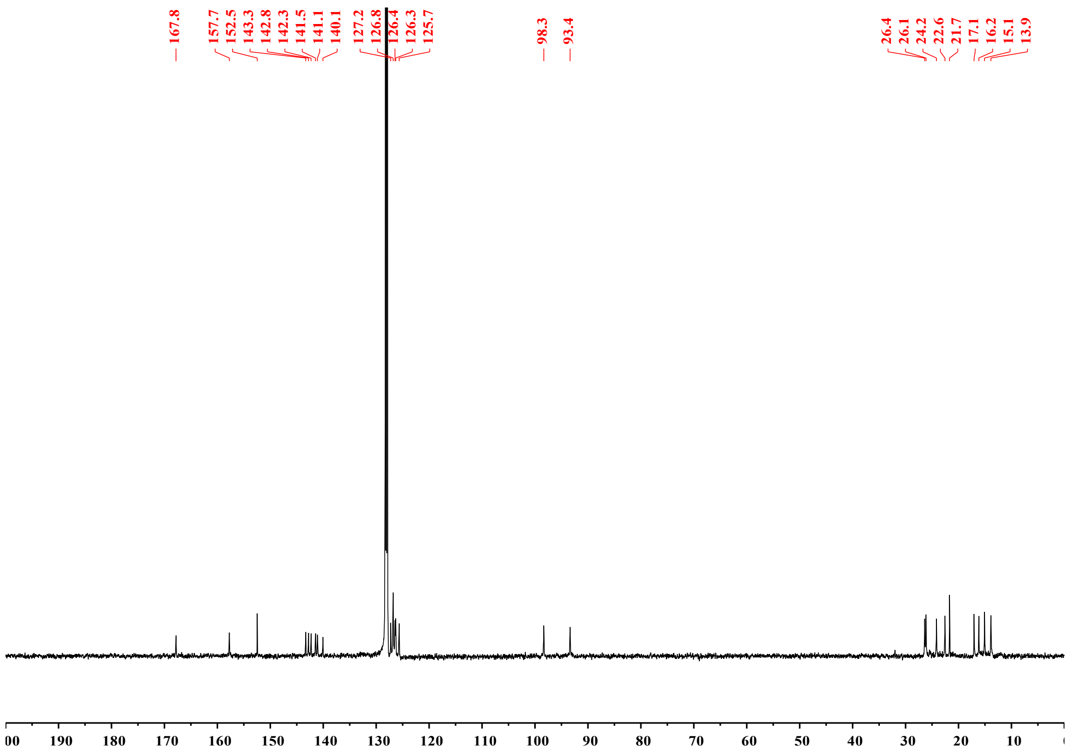

Figure *S*18. $^{13}C\{^{1}H\}$ NMR spectrum of **3** (126 MHz, C_6D_6, 298K).

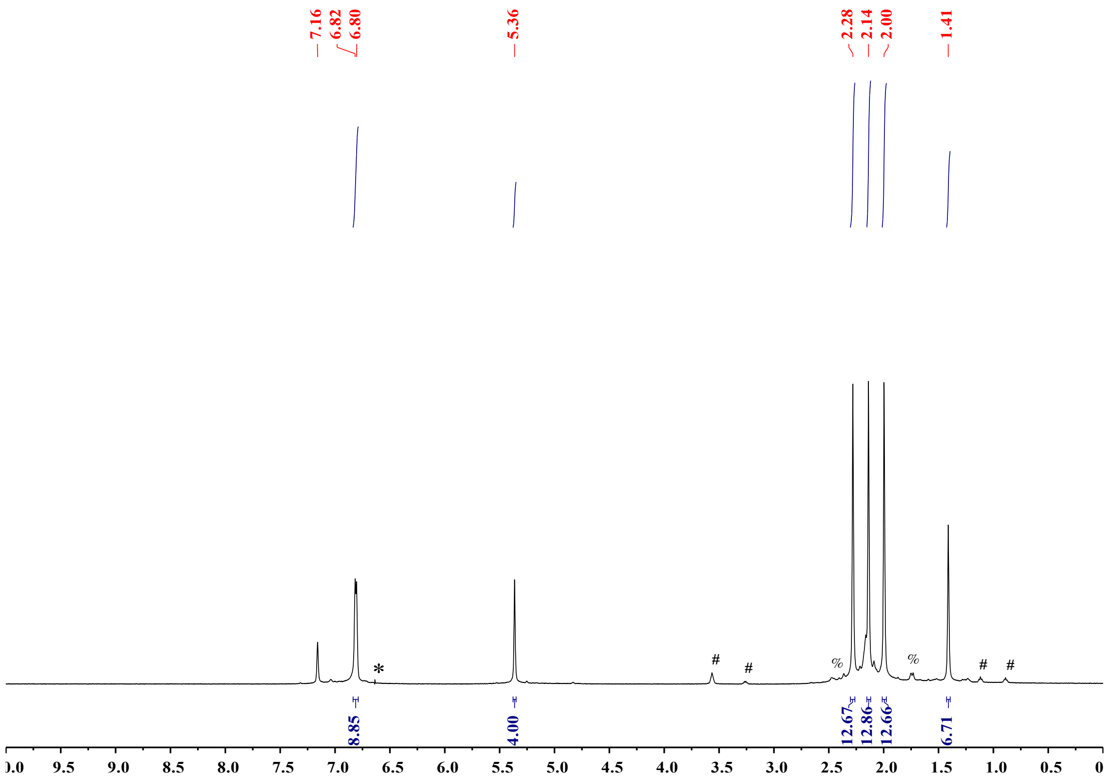

Figure *S*19. ^{1}H NMR spectrum of **4** (500 MHz, C_6D_6, 298K). (*: ghost peak; #: Hexane, Et_2O and THF; %: impurity.)

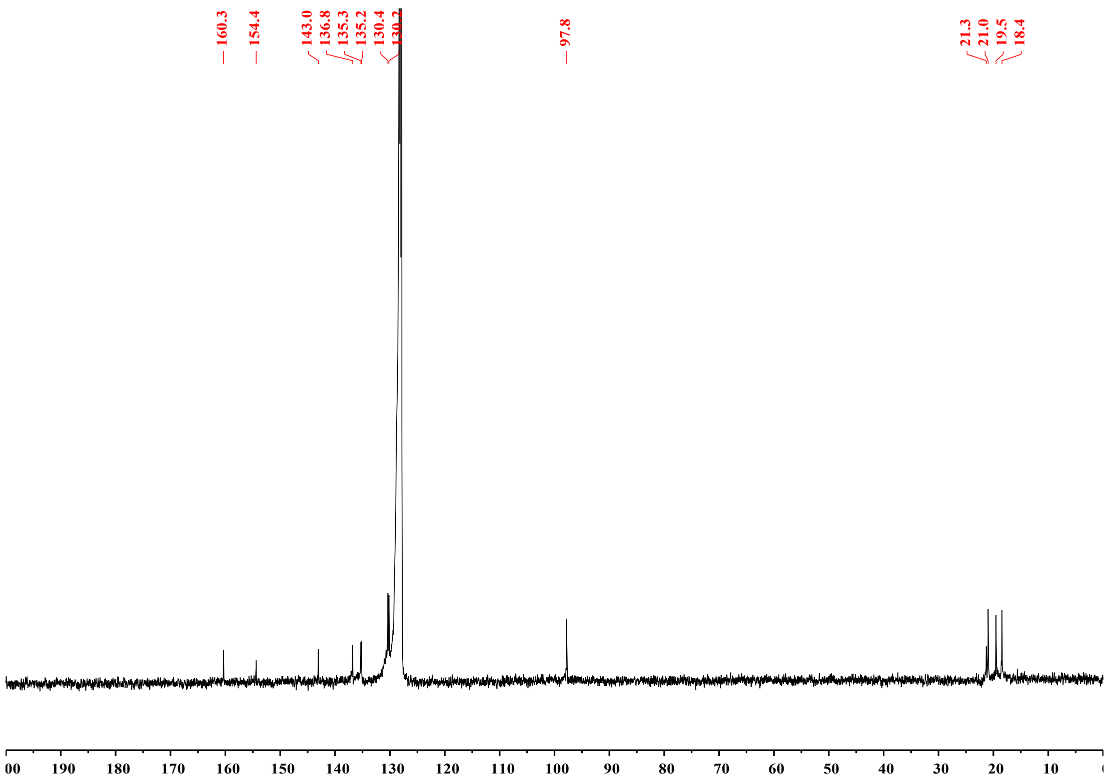

Figure *S*20. $^{13}C\{^{1}H\}$ NMR spectrum of **4** (126 MHz, C_6D_6, 298K).

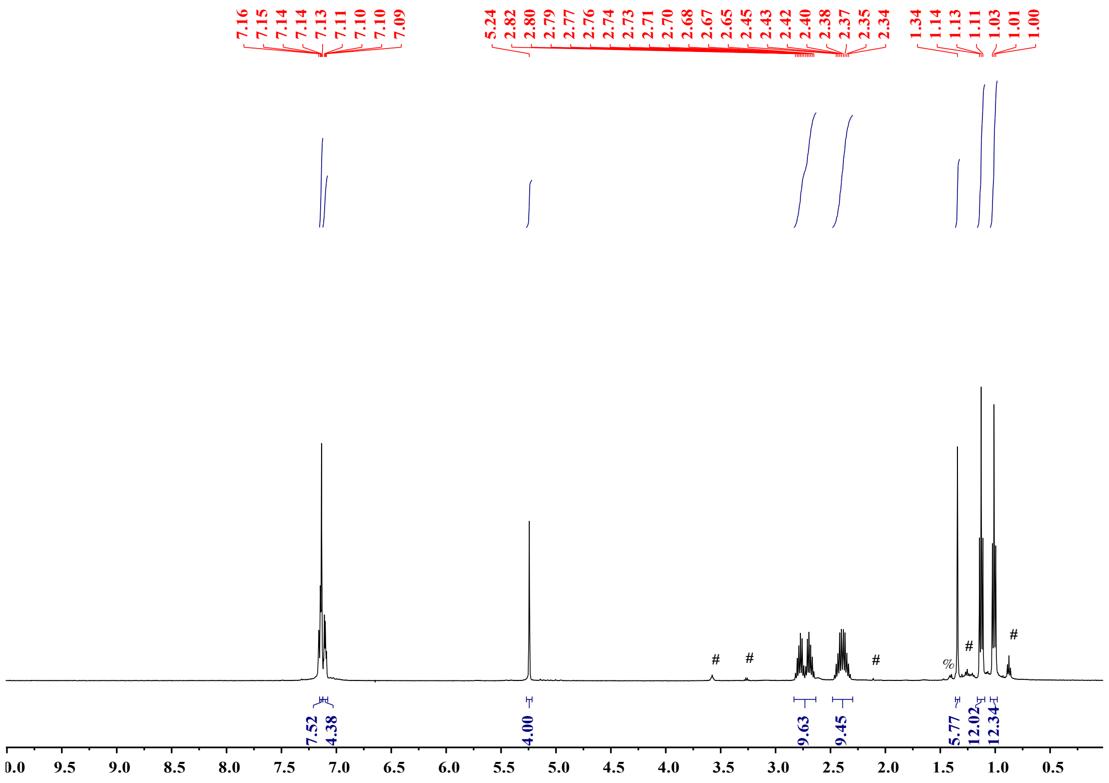

Figure S21. ^{1}H NMR spectrum of **5** (500 MHz, C_6D_6, 298K). (#: Hexane, Et_2O, Toluene and THF; %: impurity.)

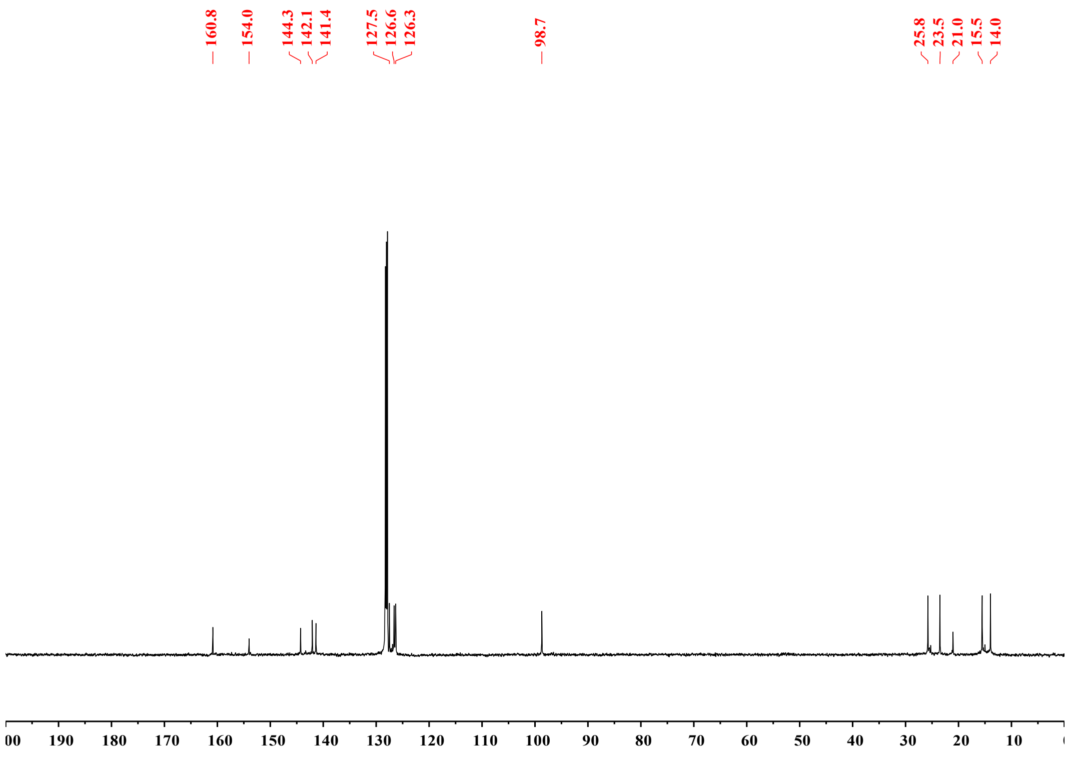

Figure S22. $^{13}C\{^1H\}$ NMR spectrum of **5** (126 MHz, C_6D_6, 298K).

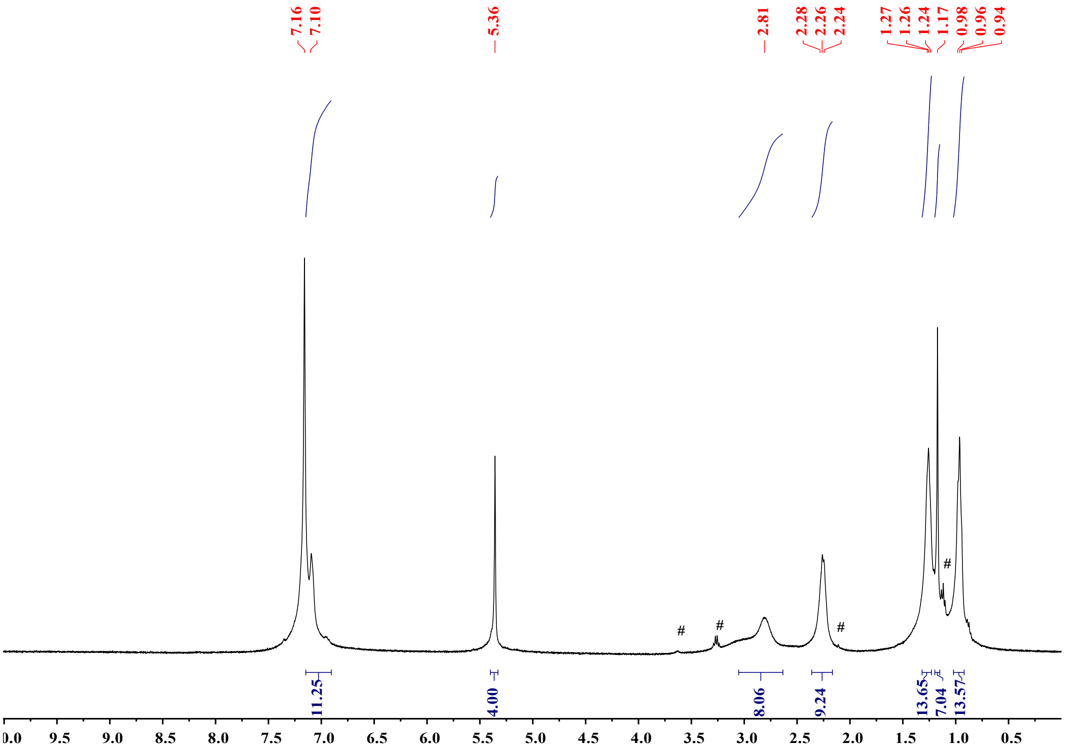

Figure *S*23. ^{1}H NMR spectrum of **6** (400 MHz, C_6D_6, 298K). ($^{\#}$: Et_2O, Toluene and THF)

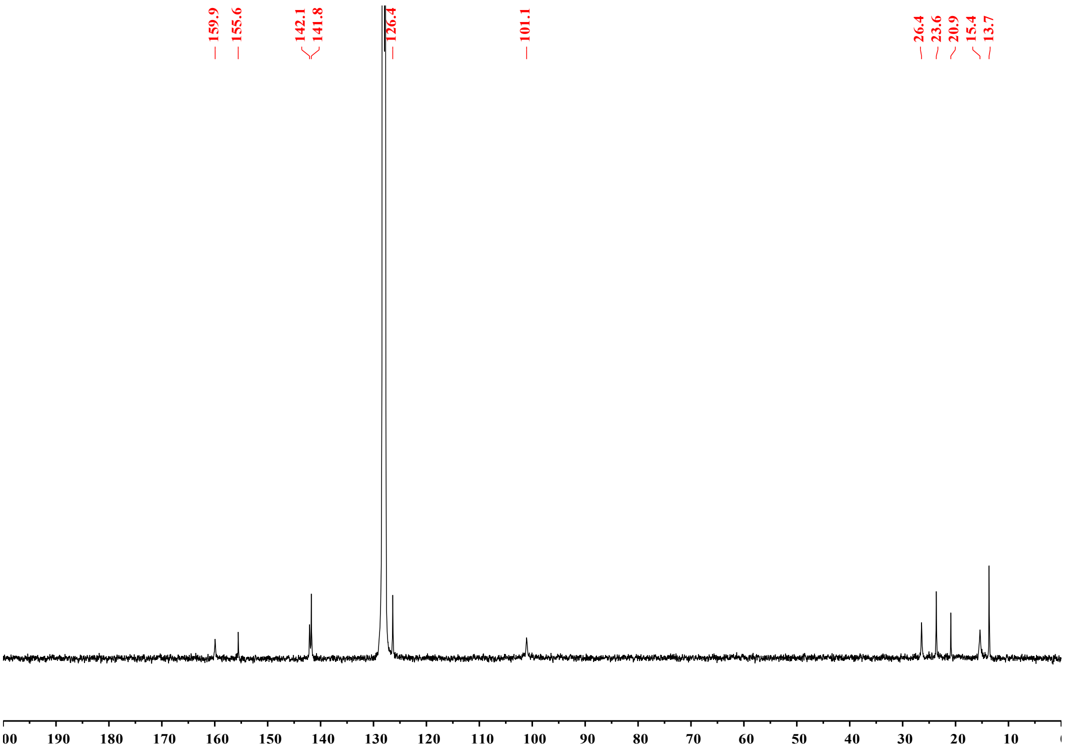

Figure *S*24. $^{13}C\{^{1}H\}$ NMR spectrum of **6** (101 MHz, C_6D_6, 298K).

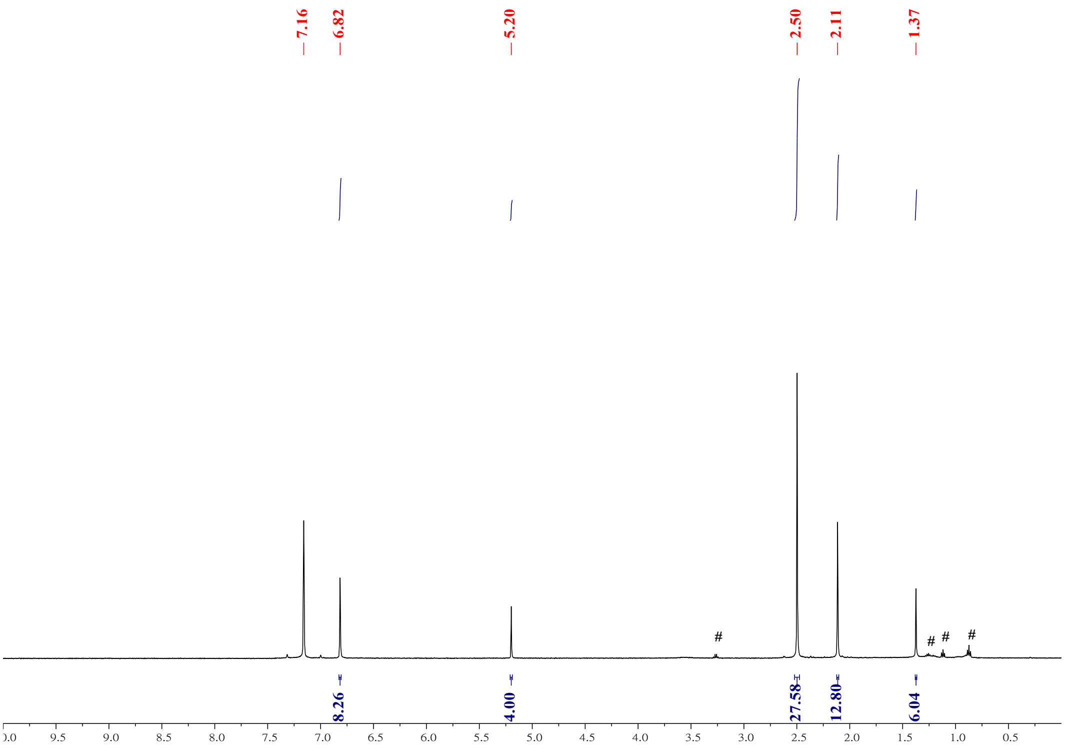

Figure S25. ^{1}H NMR spectrum of **7** (500 MHz, C_6D_6, 298K). ($^{\#}$: Hexane, Et_2O and THF)

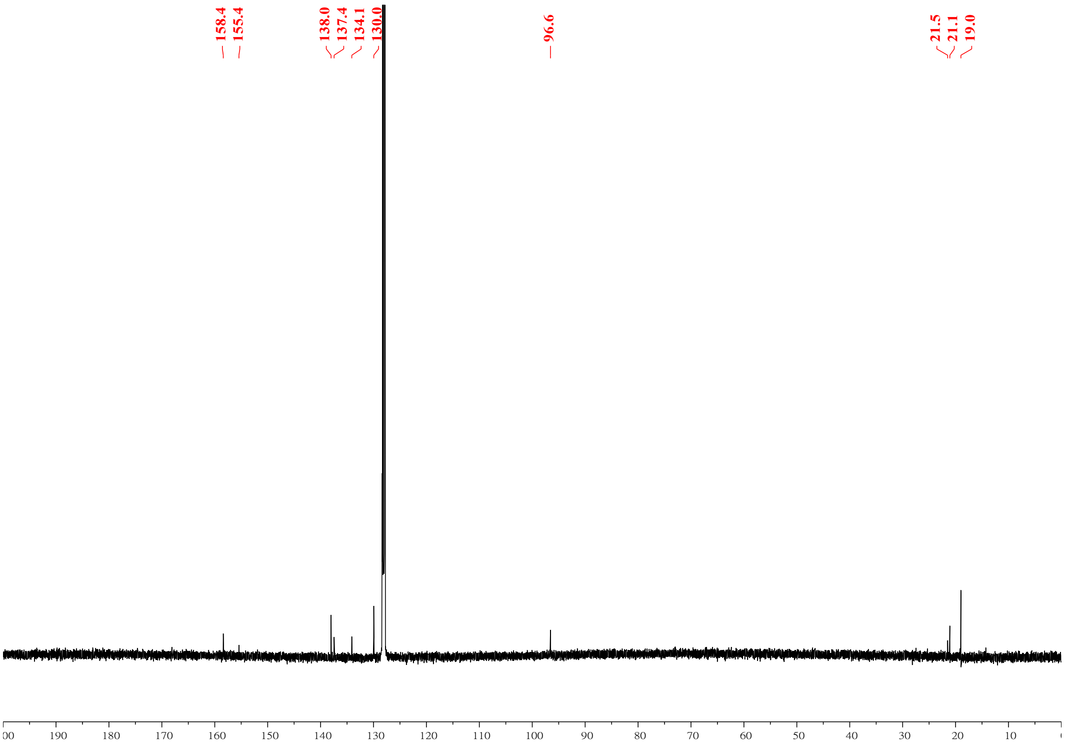

Figure S26. $^{13}C\{^{1}H\}$ NMR spectrum of **7** (126 MHz, C_6D_6, 298K).

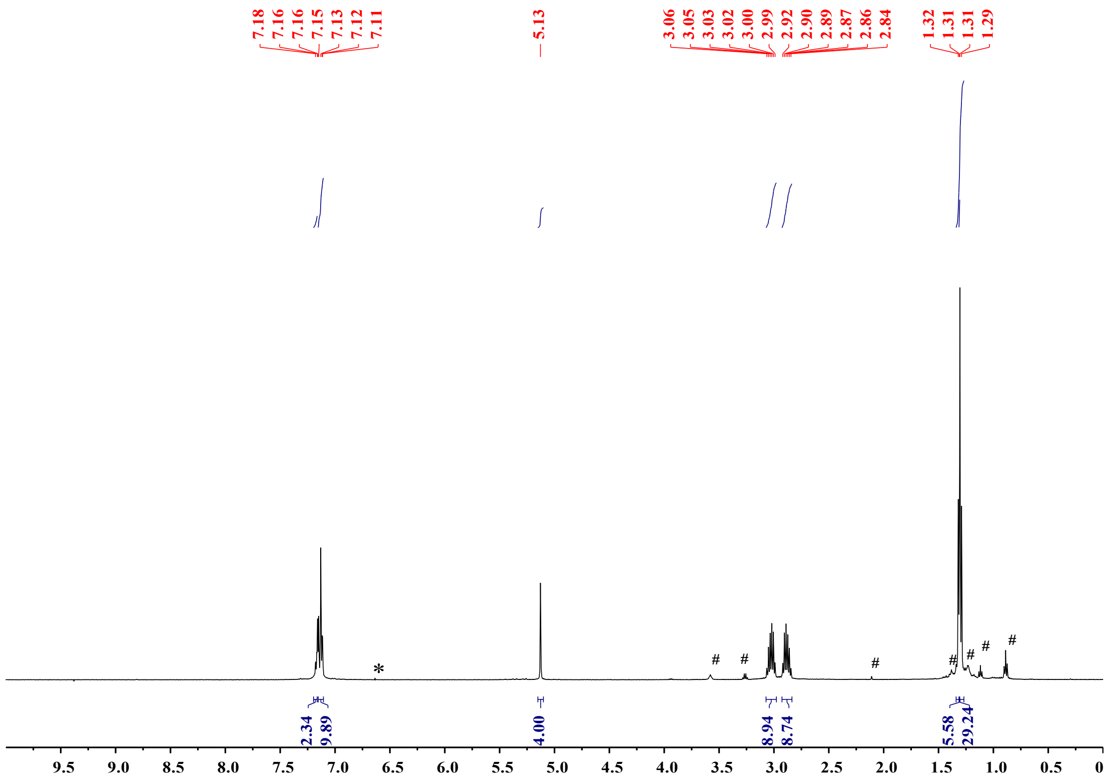

Figure *S*27. ^{1}H NMR spectrum of **8** (500 MHz, C_6D_6, 298K). ($^{\#}$: Hexane, Et_2O, Toluene and THF)

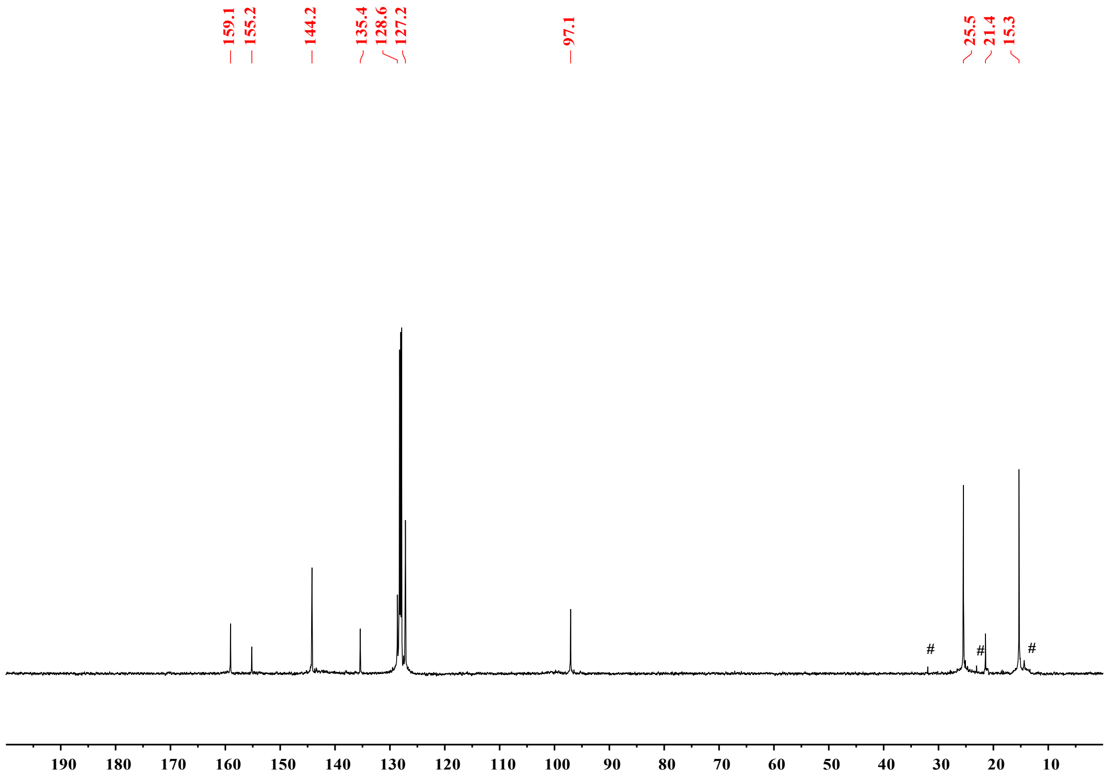

Figure *S*28. $^{13}C\{^{1}H\}$ NMR spectrum of **8** (126 MHz, C_6D_6, 298K). ($^{\#}$: Hexane)

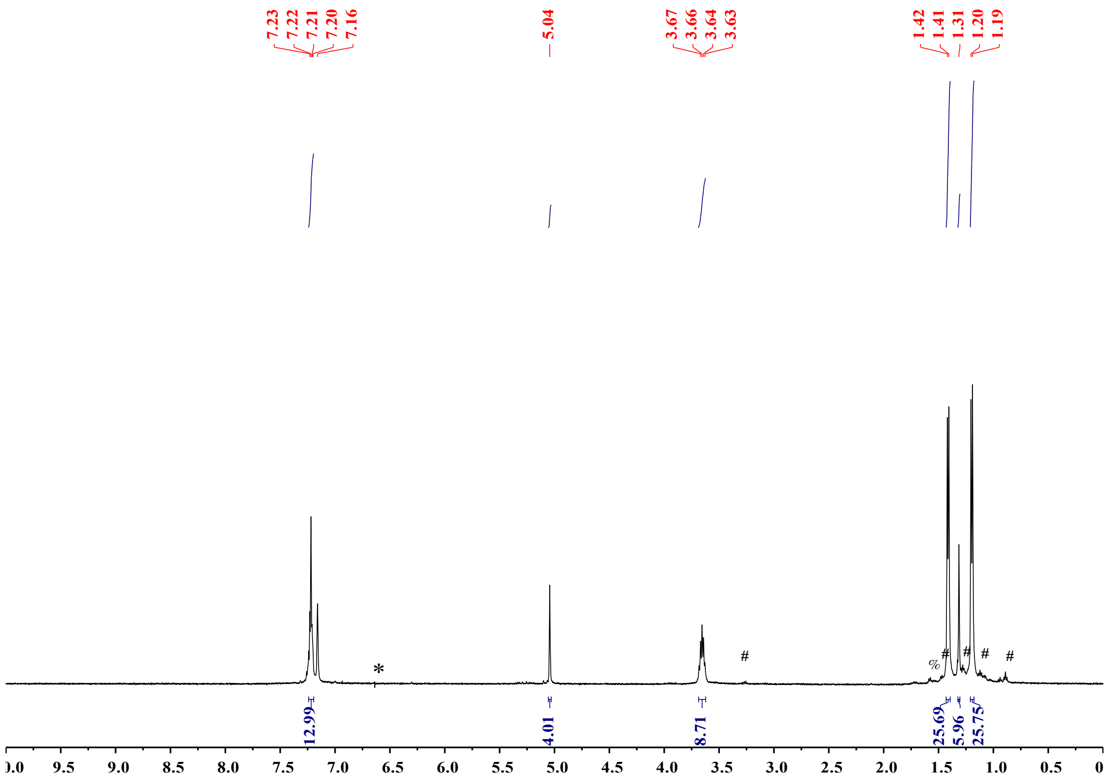

Figure *S*29. ^{1}H NMR spectrum of **9** (500 MHz, C_6D_6, 298K). (*: ghost peak; #: Hexane, Et_2O, Toluene and THF; %: impurity.)

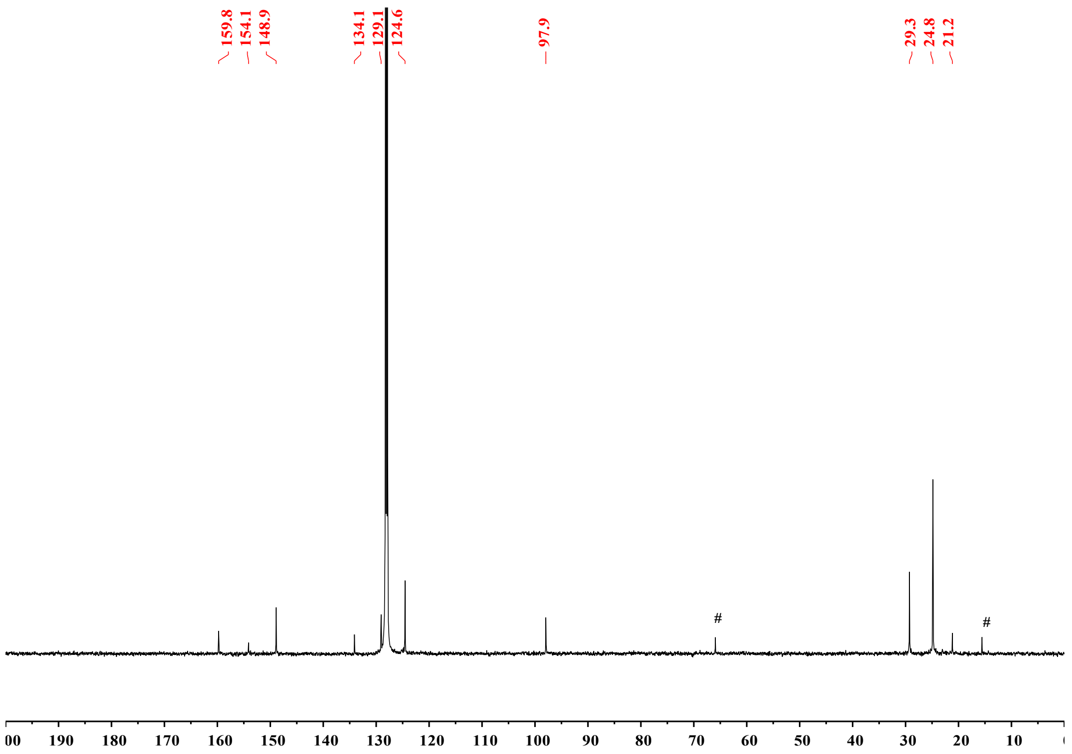

Figure *S*30. $^{13}C\{^{1}H\}$ NMR spectrum of **9** (126 MHz, C_6D_6, 298K). (#: Et_2O)

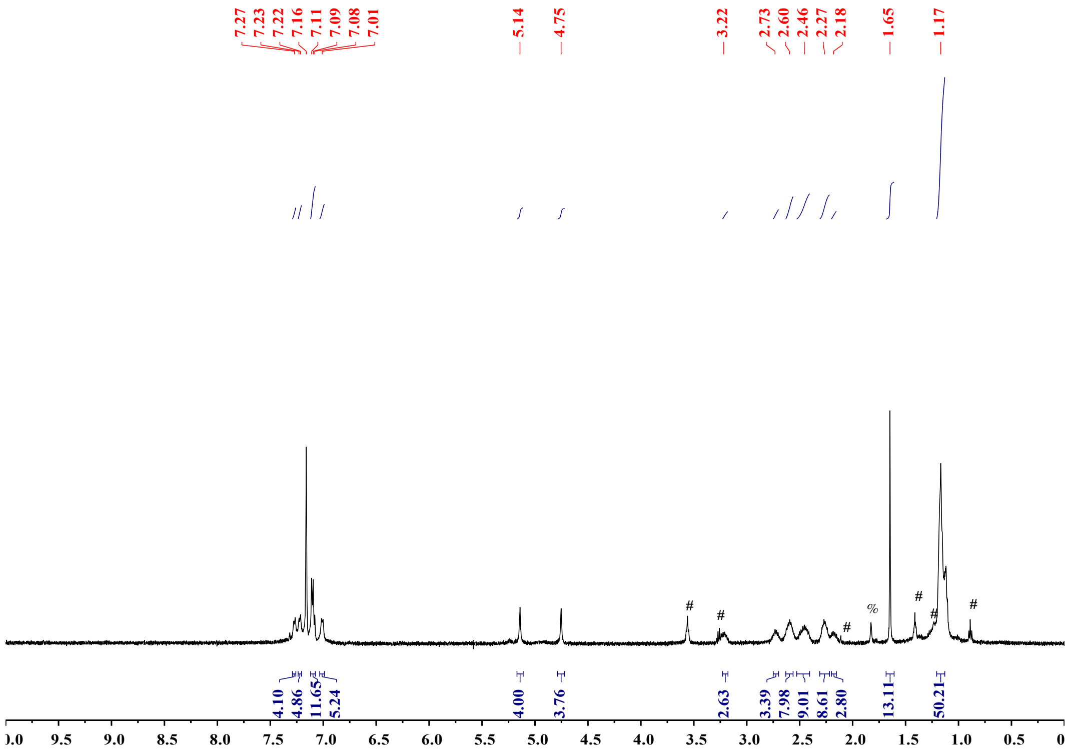

Figure *S*31. ^{1}H NMR spectrum of **10** (500 MHz, C_6D_6, 298K). ($^{\#}$: Hexane, Et_2O, Toluene and THF; $^{\%}$: impurity.)

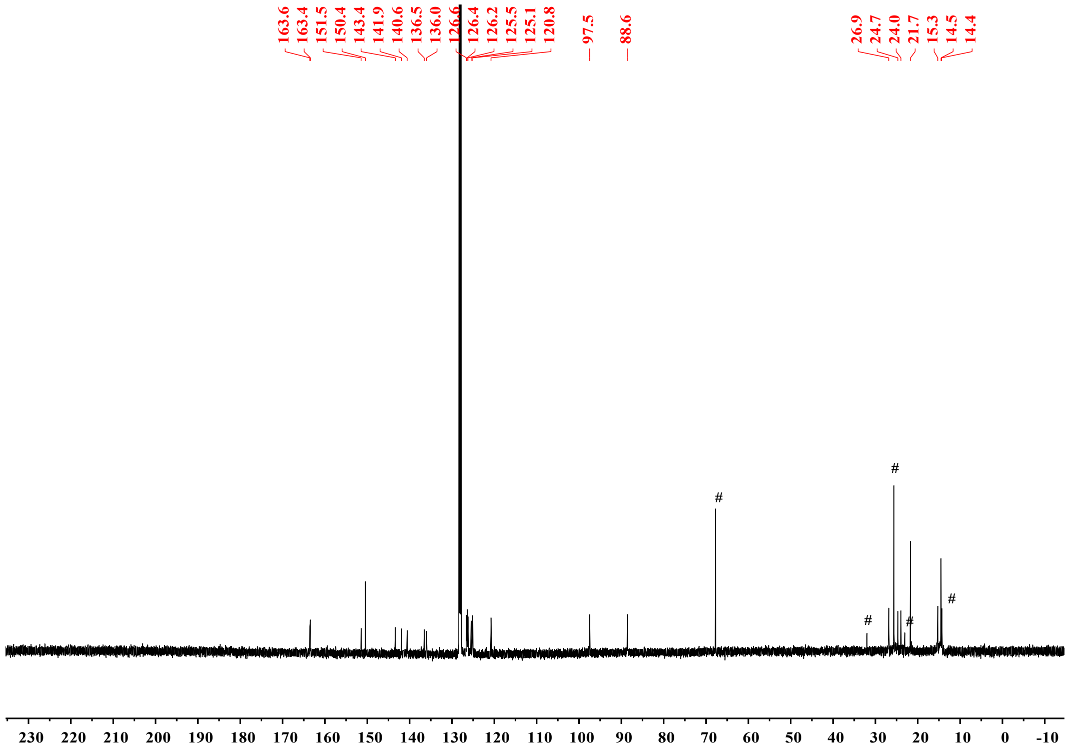

Figure *S*32. $^{13}C\{^{1}H\}$ NMR spectrum of **10** (126 MHz, C_6D_6, 298K). ($^{\#}$: Hexane and Et_2O)

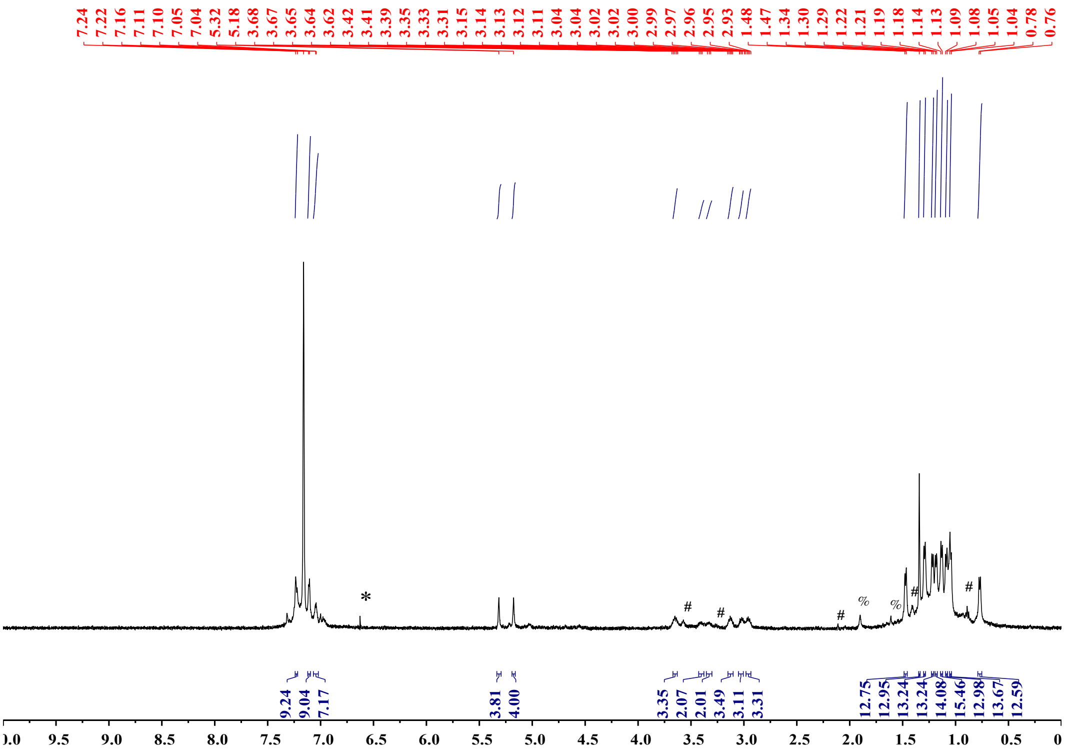

Figure *S*33. ^{1}H NMR spectrum of **11** (500 MHz, C_6D_6, 298K). (*: ghost peak; #: Hexane, Et_2O, Toluene and THF; %: impurity.)

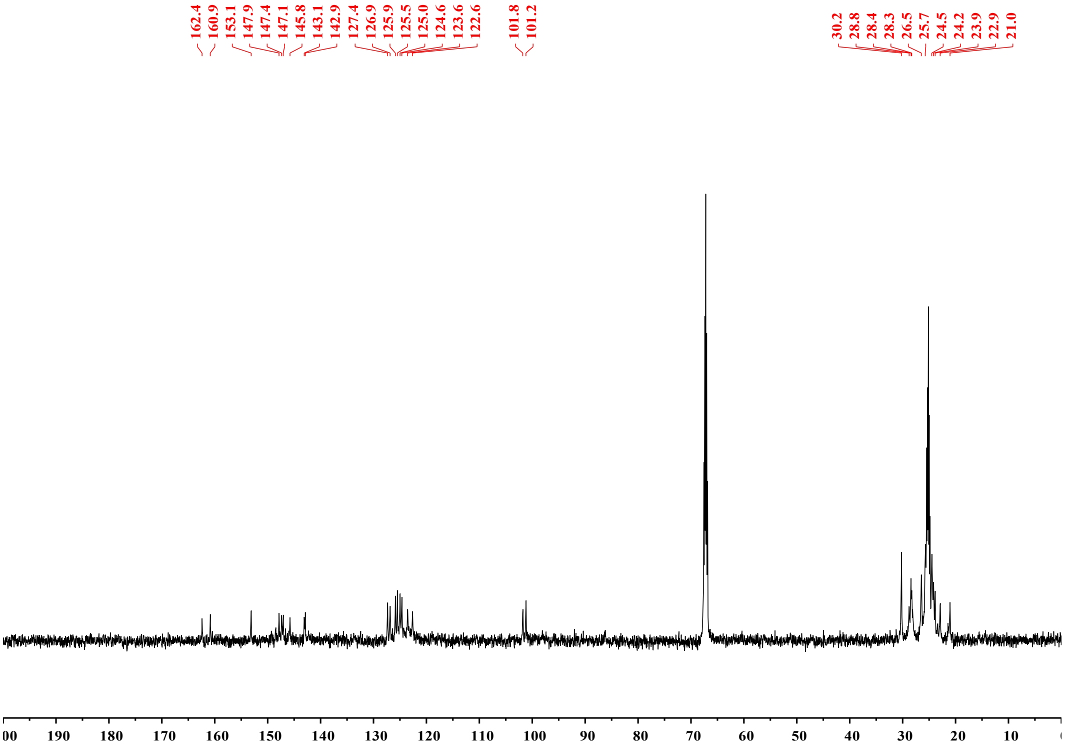

Figure *S*34. $^{13}C\{^{1}H\}$ NMR spectrum of **11** (126 MHz, d_8-THF, 298K).

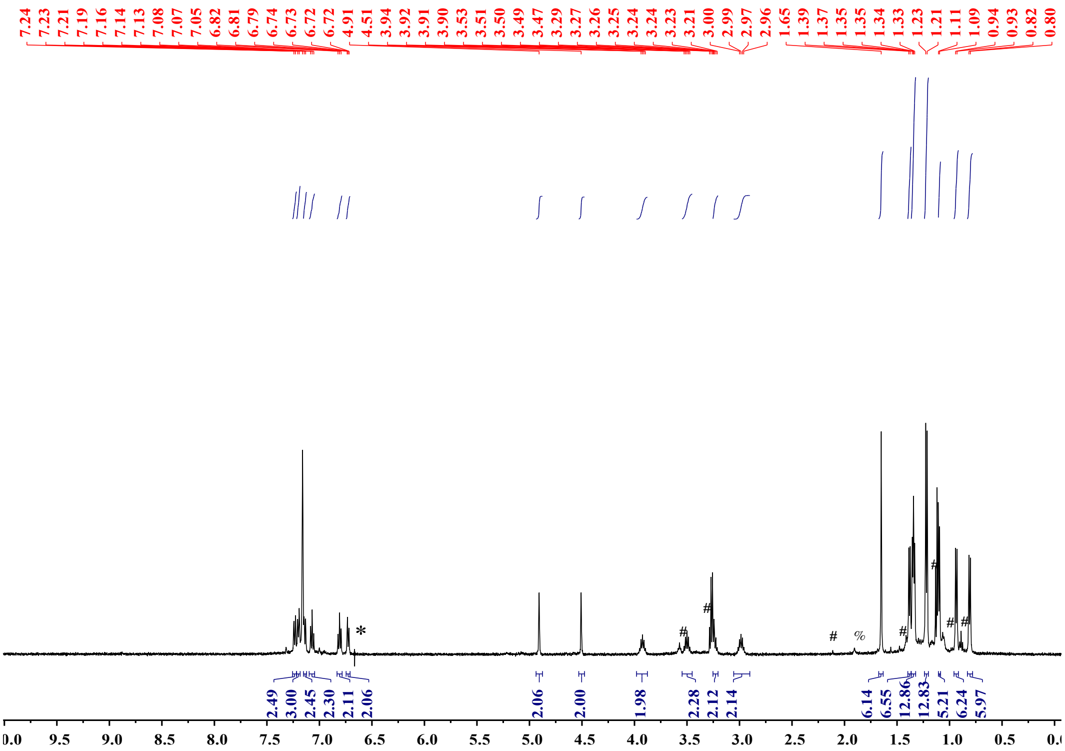

Figure *S*35. ^{1}H NMR spectrum of **12** (500 MHz, C_6D_6, 298K). (*: ghost peak; $^{\#}$: Hexane, Et_2O, Toluene and THF; $^{\%}$: impurity.)

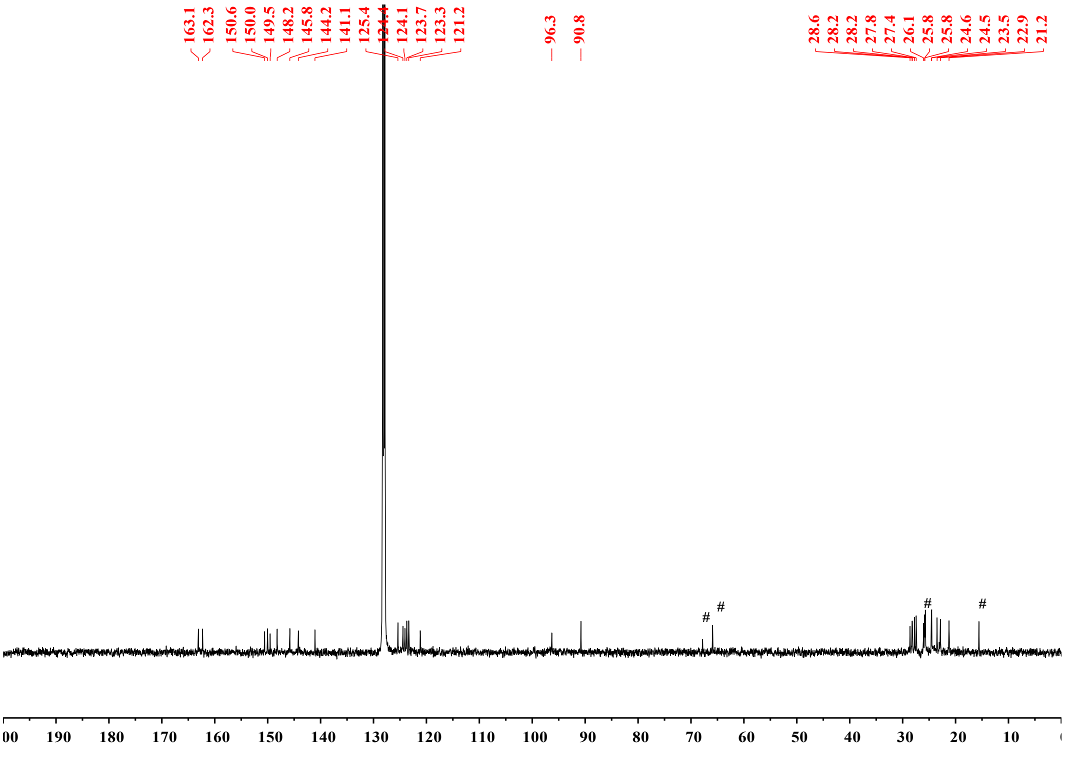

Figure *S*36. $^{13}C\{^{1}H\}$ NMR spectrum of **12** (126 MHz, C_6D_6, 298K). ($^{\#}$: Et_2O and THF)

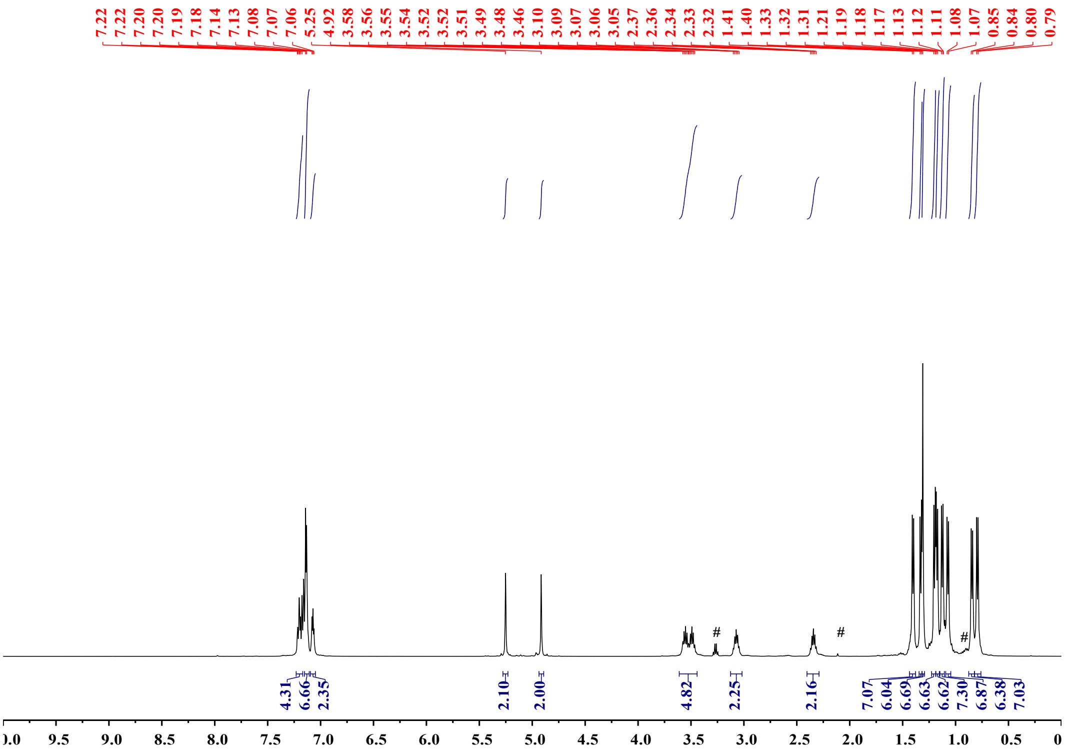

Figure *S*37. ^{1}H NMR spectrum of **15** (500 MHz, C_6D_6, 298K). ($^{\#}$: Hexane, Et_2O and Toluene)

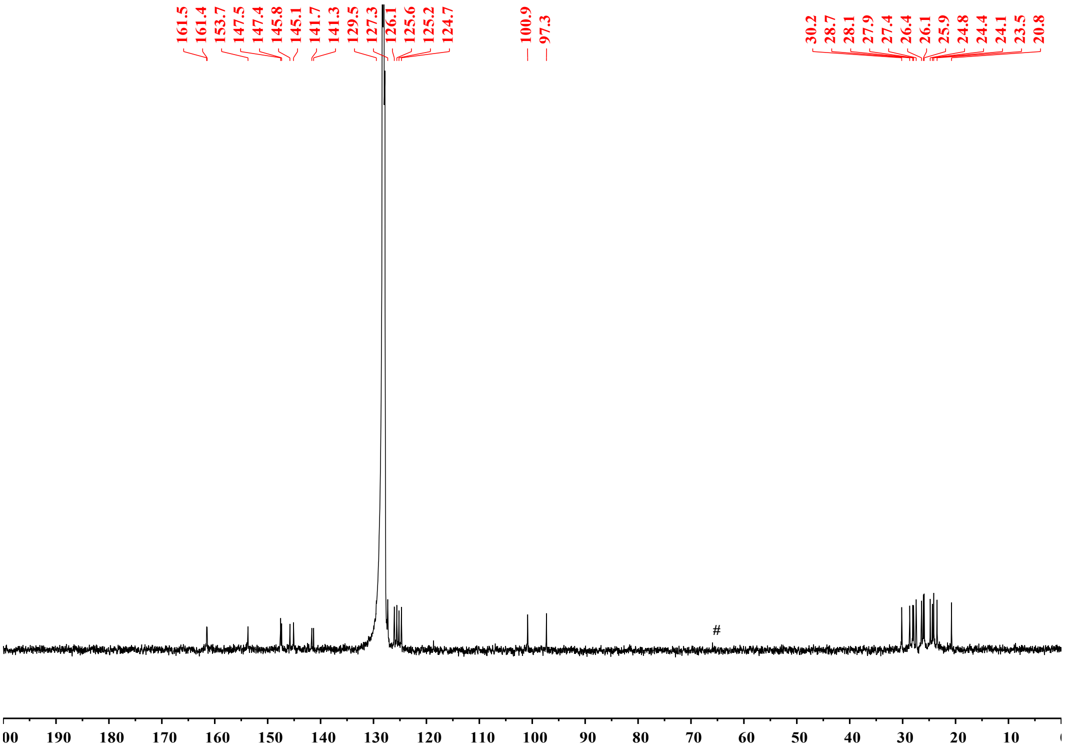

Figure *S*38. $^{13}C\{^{1}H\}$ NMR spectrum of **15** (126 MHz, C_6D_6, 298K). ($^{\#}$: Et_2O)

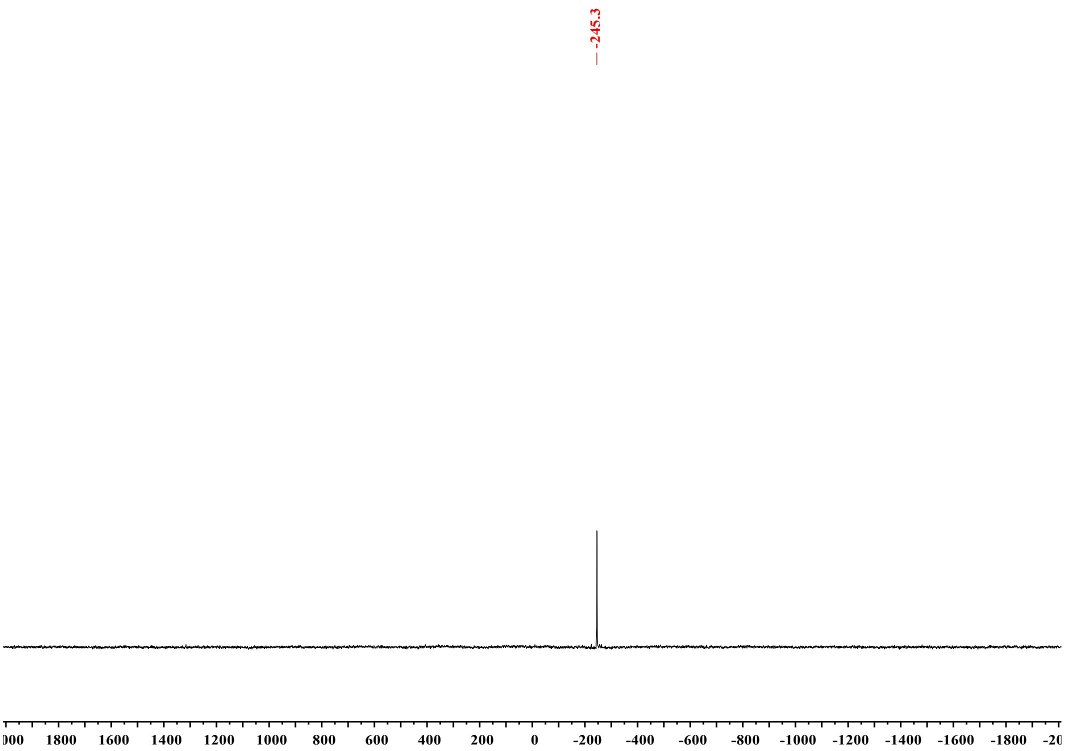

Figure *S*39. ^{119}Sn{^{1}H} NMR spectrum of **15** (187 MHz, C_6D_6, 298K).

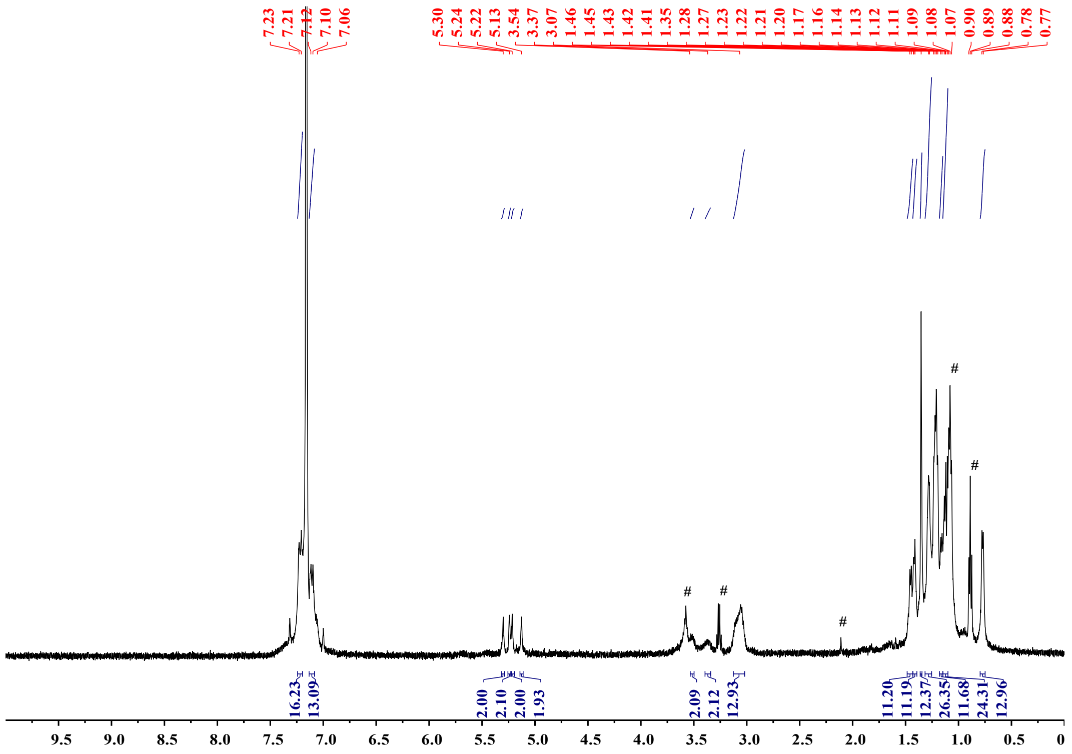

Figure *S*40. ^{1}H NMR spectrum of **16** (500 MHz, C_6D_6, 298K). ($^{\#}$: Hexane, Et_2O, Toluene and THF)

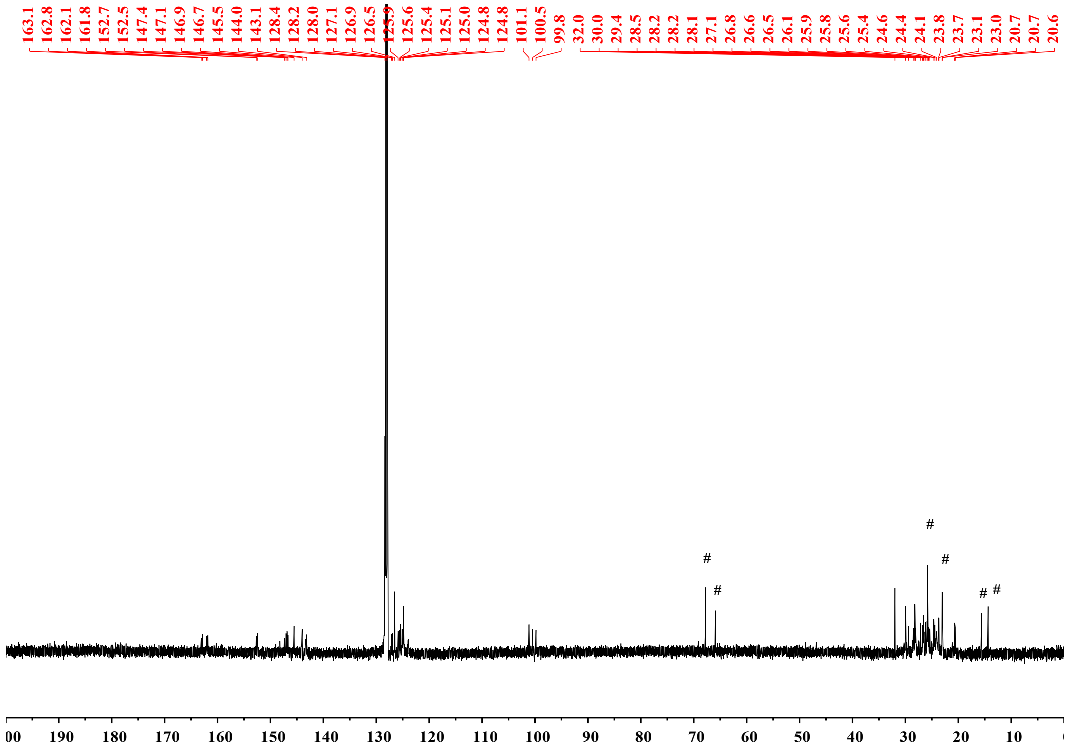

Figure *S*41. $^{13}C\{^{1}H\}$ NMR spectrum of **16** (126 MHz, C_6D_6, 298K). ($^{\#}$: Hexane, Et_2O, Toluene and THF)

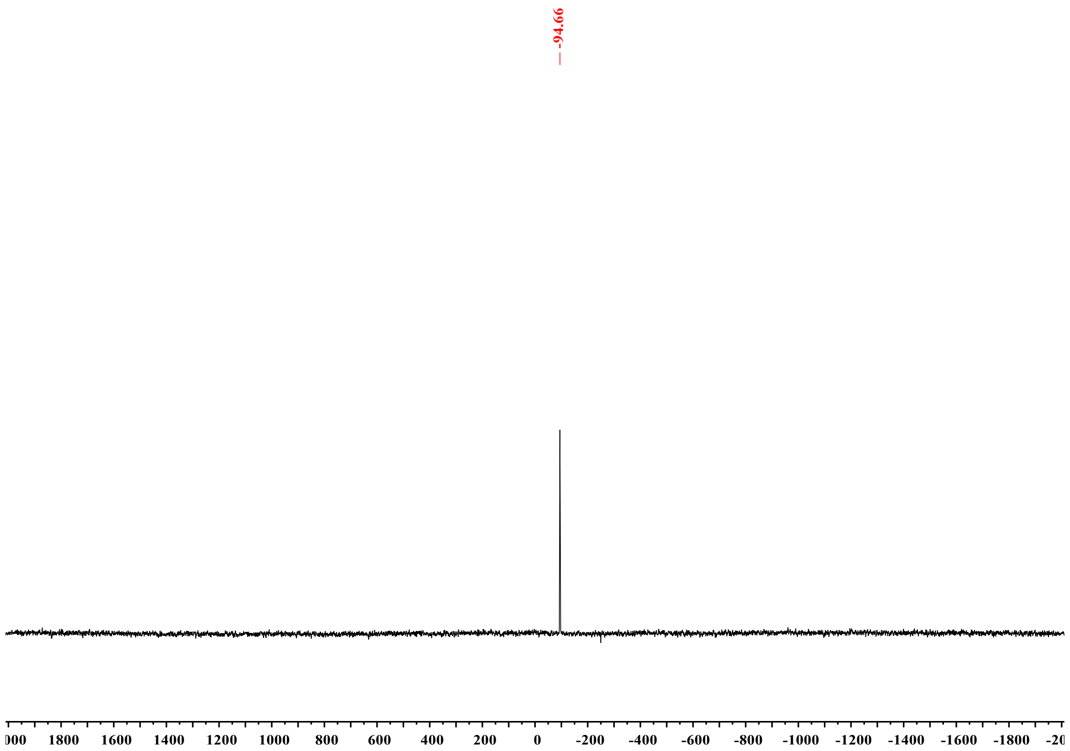

Figure *S*42. $^{119}Sn\{^{1}H\}$ NMR spectrum of **16** (187 MHz, C_6D_6, 298K).

4. Elemental analysis report

National Chung Hsing University

The Office of Research and Development, Instrument Center

Report on Elemental Analysis

Instrument Director : Prof. Chen-Yu Yeh

Operator : I-Chuan Chen

Note: This result is for academic use only, not to be used for any judicial or commercial advertising purpose.

Information of Submitted Sample

Web NO:	SEA0001002021070012		
Department :	National Tsing Hua University department of chemistry		Date of
Supervisor :	Prof. Yi-Chou Tsai	Receive:	2021.07.07
User name :	Wei -Ting Kuo	completion:	2021.07.07

Results of Analysis

Sample code	*Weight(mg)*	*N %*	*C %*	*H %*	*O %*	*S %*	*Repeat*	*Charge*
WT3-O9C-237	2.437	7.91	69.57	7.71			1	$ 320

Instrumentation: *Elementar vario EL CUBE (CHN-OS Rapid, German), Accuracy: 0.1%, Precision: 0.2%*

	Standard sample	*N %*	*C %*	*H %*	*O %*	*S %*
★	*Acetanilide*	*10.36*	*71.09*	*6.71*		
	Benzoic acid				*26.20*	
	Sulfanilic acid	*8.09*	*41.60*	*4.07*		*18.50*
	Daily standard	*10.30*	*71.10*	*6.62*		

Figure *S*43. Elemental analysis report of **1**.

National Chung Hsing University

The Office of Research and Development, Instrument Center

Report on Elemental Analysis

Instrument Director : Prof. Jen-Fon Jen

Operator : I-Chuan Chen

Note: This result is for academic use only, not to be used for any judicial or commercial advertising purpose.

Information of Submitted Sample

Web NO	SEA0001002017110024		
Department :	Chemistry		Date of
Supervisor :	Prof. Yi-Chou Tsai	Receive:	2017.11.21
User name :	Prof. Yi-Chou Tsai	completion:	2017.11.21

Results of Analysis

Sample code	*Weight(mg)*	*N %*	*C %*	*H %*	*O %*	*S %*	*Repeat*	*Charge*
K65	4.161	9.46	67.70	6.80			1	$ 2,000
	4.821	9.47	67.81	6.77				

Instrumentation: *Elementar vario EL CUBE (CHN-OS Rapid, German), Accuracy: 0.1%, Precision: 0.2%*

	Standard sample	*N %*	*C %*	*H %*	*O %*	*S %*
★	*Acetanilide*	*10.36*	*71.09*	*6.71*		
	Benzoic acid				*26.20*	
	Sulfanilic acid	*8.09*	*41.60*	*4.07*		*18.50*
	Daily standard	*10.35*	*71.02*	*6.75*		

Figure *S*44. Elemental analysis report of **2**.

Precision Instrumentation Center--Elemental Analysis Report

College of Science, National Taiwan University

民國 86 年申請服務標章的校徽

Remark: 1. This result is for academic use only, not to be used for any judicial or commercial advertising purpose.

2. Instrument： elementar Vario EL cube 型 (for NCSH, German)

Accuracy: ±0.1%
Precision: ±0.2%
Inaccuracy of instrument: ±0.3%

3. Instrument Director: Prof. Wong, Ken-Tsung   Operator: Ching-Wei Lu

Web. NO.	52021060002
User name	Wei Ting Kuo
Supervisor	Prof. Yi-Chou Tsai
University or Institute	National Tsing Hua University Department of chemistry
Acceptance date	2021/6/23
Analysis date	2021/6/23

Sample code	Date Time	Weight(mg)	Grp	N%	C%	S%	H%	Repeat	Charge
acetanilide	23.06.2021	3.629	standard	10.360	71.090	0.000	6.710		
standard 測出值	23.06.2021	4.018		10.367	71.219	0.000	6.759		
WT3-G2L-H-229	23.06.2021	2.936	sensitive	9.061	68.579		7.142	1	$ 400
								1	$ 400

Figure *S*45. Elemental analysis report of **3**.

National Chung Hsing University

The Office of Research and Development, Instrument Center

Report on Elemental Analysis

Instrument Director : Prof. Chen-Yu Yeh

Operator : I-Chuan Chen

Note: This result is for academic use only, not to be used for any judicial or commercial advertising purpose.

Information of Submitted Sample

Web NO :	BSEA0001002021120035		
Department :	National Tsing Hua University Department of chemistry		Date of
Supervisor :	Prof. Yi-Chou Tsai	Receive:	2021.12.23
User name :	Wei-Ting Kuo	completion:	2021.12.23

Results of Analysis

Sample code	*Weight(mg)*	*N %*	*C %*	*H %*	*O %*	*S %*	*Repeat*	*Charge*
WT4-G5Q-H-65	3.072	8.66	61.45	6.00				$ 320

Instrumentation: *Elementar vario EL CUBE (CHN-OS Rapid, German), Accuracy: 0.1%, Precision: 0.2%*

	Standard sample	*N %*	*C %*	*H %*	*O %*	*S %*
★	*Acetanilide*	*10.36*	*71.09*	*6.71*		
	Benzoic acid				*26.20*	
	Sulfanilic acid	*8.09*	*41.60*	*4.07*		*18.50*
	Daily standard	*10.36*	*71.16*	*6.67*		

Figure *S*46. Elemental analysis report of **4**.

National Chung Hsing University

The Office of Research and Development, Instrument Center

Report on Elemental Analysis

Instrument Director : Prof. Jen-Fon Jen

Operator : I-Chuan Chen

Note: This result is for academic use only, not to be used for any judicial or commercial advertising purpose.

Information of Submitted Sample

Web NO	SEA0001002018050036		
Department :	Chemistry		Date of
Supervisor :	Prof. Yi-Chou Tsai	Receive:	2018.05.29
User name :	Prof. Yi-Chou Tsai	completion:	2018.05.29

Results of Analysis

Sample code	*Weight(mg)*	*N %*	*C %*	*H %*	*O %*	*S %*	*Repeat*	*Charge*
K87	2.668	8.30	62.59	6.39			1	$ 2,000
	3.048	8.26	62.35	6.47				

Instrumentation: *Elementar vario EL CUBE (CHN-OS Rapid, German), Accuracy: 0.1%, Precision: 0.2%*

	Standard sample	*N %*	*C %*	*H %*	*O %*	*S %*
★	*Acetanilide*	*10.36*	*71.09*	*6.71*		
	Benzoic acid				*26.20*	
	Sulfanilic acid	*8.09*	*41.60*	*4.07*		*18.50*
	Daily standard	*10.40*	*71.09*	*6.70*		

Figure *S*47. Elemental analysis report of **5**.

National Chung Hsing University

The Office of Research and Development, Instrument Center

Report on Elemental Analysis

Instrument Director : Prof. Chen-Yu Yeh

Operator : I-Chuan Chen

Note: This result is for academic use only, not to be used for any judicial or commercial advertising purpose.

Information of Submitted Sample

Web NO	SEA0001002020010016		
Department :	National Tsing Hua University department of chemistry		Date of
Supervisor :	Prof. Yi-Chou Tsai	Receive:	2020.01.15
User name :	Wei –Ting Kuo	Completion:	2020.01.15

Results of Analysis

Sample code	*Weight(mg)*	*N %*	*C %*	*H %*	*O %*	*S %*	*Repeat*	*Charge*
WT3-C_6-F1G-28	3.564	6.71	51.25	5.09			1	$ 2,000

Instrumentation: *Elementar vario EL CUBE (CHN-OS Rapid, German), Accuracy: 0.1%, Precision: 0.2%*

	Standard sample	*N %*	*C %*	*H %*	*O %*	*S %*
★	*Acetanilide*	*10.36*	*71.09*	*6.71*		
	Benzoic acid				*26.20*	
	Sulfanilic acid	*8.09*	*41.60*	*4.07*		*18.50*
	Daily standard	*10.36*	*71.00*	*6.71*		

Figure *S*48. Elemental analysis report of **6**.

National Chung Hsing University

The Office of Research and Development, Instrument Center

Report on Elemental Analysis

Instrument Director : Prof. Chen-Yu Yeh

Operator : I-Chuan Chen

Note: This result is for academic use only, not to be used for any judicial or commercial advertising purpose.

Information of Submitted Sample

Web NO :	BSEA0001002024010043		
Department :	National Tsing Hua University		Date of
	Department of chemistry		
Supervisor :	Prof. Yi-Chou Tsai	Receive:	2024.02.01
User name :	Wei-Ting Kuo	completion:	2024.02.01

Results of Analysis

Sample code	*Weight(mg)*	*N %*	*C %*	*H %*	*O %*	*S %*	*Repeat*	*Charge*
WT5-Mes-Ge_4Cl_4-73	1.347	6.93	50.17	4.77				$ 320

Instrumentation: *Elementar vario EL CUBE (CHN-OS Rapid, German), Accuracy: 0.1%, Precision: 0.2%*

	Standard sample	*N %*	*C %*	*H %*	*O %*	*S %*
★	*Acetanilide*	*10.36*	*71.09*	*6.71*		
	Benzoic acid				*26.20*	
	Sulfanilic acid	*8.09*	*41.60*	*4.07*		*18.50*
	Daily standard	*10.40*	*71.04*	*6.67*		

Figure *S*49. Elemental analysis report of **7**.

National Chung Hsing University

The Office of Research and Development, Instrument Center

Report on Elemental Analysis

Instrument Director : Prof. Jen-Fon Jen

Operator : I-Chuan Chen

Note: This result is for academic use only, not to be used for any judicial or commercial advertising purpose.

Information of Submitted Sample

Web NO	SEA0001002018040007		
Department :	Chemistry		Date of
Supervisor :	Prof. Yi-Chou Tsai	Receive:	2018.04.10
User name :	Prof. Yi-Chou Tsai	completion:	2018.04.10

Results of Analysis

Sample code	*Weight(mg)*	*N %*	*C %*	*H %*	*O %*	*S %*	*Repeat*	*Charge*
K83	1.696	6.92	52.09	5.16			1	$ 2,000
	2.745	6.93	52.11	5.14				

Instrumentation: *Elementar vario EL CUBE (CHN-OS Rapid, German), Accuracy: 0.1%, Precision: 0.2%*

	Standard sample	*N %*	*C %*	*H %*	*O %*	*S %*
★	*Acetanilide*	*10.36*	*71.09*	*6.71*		
	Benzoic acid				*26.20*	
	Sulfanilic acid	*8.09*	*41.60*	*4.07*		*18.50*
	Daily standard	*10.39*	*71.13*	*6.70*		

Figure *S*50. Elemental analysis report of **8**.

Precision Instrumentation Center--Elemental Analysis Report

College of Science, National Taiwan University

民國 86 年申請服務標章的校徽

Remark: 1. This result is for academic use only, not to be used for any judicial or commercial advertising purpose.

2. Instrument： elementar Vario EL cube 型 (for NCSH, German)

Accuracy: ±0.1%
Precision: ±0.2%
Inaccuracy of instrument: ±0.3%

3. Instrument Director: Prof. Wong, Ken-Tsung Operator: Ching-Wei Lu

Web. NO.	52020110004
User name	Wei Ting Kuo
Supervisor	Prof. Yi-Chou Tsai
University or Institute	National Tsing Hua University Department of chemistry
Acceptance date	2020/12/16
Analysis date	2020/12/16

Sample code	Date Time	Weight(mg)	Grp	N%	C%	S%	H%	Repeat	Charge
acetanilide	16.12.2020	4.228	standard	10.360	71.090	0.000	6.710		
standard daily data	16.12.2020	3.434		10.364	71.124	0.000	6.649		
WT3-M3C-170	16.12.2020	4.169	sensitive	6.253	55.231		6.056	1	$ 2,000
								1	$ 2,000

Figure *S*51. Elemental analysis report of **9**.

National Chung Hsing University

The Office of Research and Development, Instrument Center

Report on Elemental Analysis

Instrument Director : Prof. Chen-Yu Yeh

Operator : I-Chuan Chen

Note: This result is for academic use only, not to be used for any judicial or commercial advertising purpose.

Information of Submitted Sample

Web NO :	BSEA0001002022030005		
Department :	National Tsing Hua University Department of chemistry		Date of
Supervisor :	Prof. Yi-Chou Tsai	Receive:	2022.03.03
User name :	Wei-Ting Kuo	completion:	2022.03.03

Results of Analysis

Sample code	*Weight(mg)*	*N %*	*C %*	*H %*	*O %*	*S %*	*Repeat*	*Charge*
WT4-O6M-H-68	2.463	8.62	61.34	5.98				$ 320

Instrumentation: *Elementar vario EL CUBE (CHN-OS Rapid, German), Accuracy: 0.1%, Precision: 0.2%*

	Standard sample	*N %*	*C %*	*H %*	*O %*	*S %*
★	*Acetanilide*	*10.36*	*71.09*	*6.71*		
	Benzoic acid				*26.20*	
	Sulfanilic acid	*8.09*	*41.60*	*4.07*		*18.50*
	Daily standard	*10.39*	*71.03*	*6.69*		

Figure *S*52. Elemental analysis report of **10**.

Precision Instrumentation Center--Elemental Analysis Report

College of Science, National Taiwan University

民國 86 年申請服務標章的校徽

Remark: 1. This result is for academic use only, not to be used for any judicial or commercial advertising purpose.

2. Instrument： elementar Vario EL cube 型 (for NCSH, German)

Accuracy: ± 0.1%
Precision: ± 0.2%
Inaccuracy of instrument: ± 0.3%

3. Instrument Director: Prof. Wong, Ken-Tsung Operator: Ching-Wei Lu

Web. NO.	52021060002
User name	Wei Ting Kuo
Supervisor	Prof. Yi-Chou Tsai
University or Institute	National Tsing Hua University Department of chemistry
Acceptance date	2021/6/23
Analysis date	2021/6/23

Sample code	Date Time	Weight(mg)	Grp	N%	C%	S%	H%	Repeat	Charge
acetanilide	23.06.2021	3.629	standard	10.360	71.090	0.000	6.710		
standard 測出值	23.06.2021	4.018		10.367	71.219	0.000	6.759		
WT3-M7H-235	23.06.2021	3.291	sensitive	7.074	63.263		7.173	1	$ 400
								1	$ 400

Figure *S*53. Elemental analysis report of **11**.

National Chung Hsing University

The Office of Research and Development, Instrument Center

Report on Elemental Analysis

Instrument Director : Prof. Chen-Yu Yeh

Operator : I-Chuan Chen

Note: This result is for academic use only, not to be used for any judicial or commercial advertising purpose.

Information of Submitted Sample

Web NO:	SEA0001002021070012		
Department :	National Tsing Hua University department of chemistry		Date of
Supervisor :	Prof. Yi-Chou Tsai	Receive:	2021.07.07
User name :	Wei -Ting Kuo	completion:	2021.07.07

Results of Analysis

Sample code	*Weight(mg)*	*N %*	*C %*	*H %*	*O %*	*S %*	*Repeat*	*Charge*
WT4-P4B-3	2.733	7.28	65.55	7.34			1	$ 320

Instrumentation: *Elementar vario EL CUBE (CHN-OS Rapid, German), Accuracy: 0.1%, Precision: 0.2%*

	Standard sample	*N %*	*C %*	*H %*	*O %*	*S %*
★	*Acetanilide*	*10.36*	*71.09*	*6.71*		
	Benzoic acid				*26.20*	
	Sulfanilic acid	*8.09*	*41.60*	*4.07*		*18.50*
	Daily standard	*10.30*	*71.10*	*6.62*		

Figure *S*54. Elemental analysis report of **12.**

National Chung Hsing University

The Office of Research and Development, Instrument Center

Report on Elemental Analysis

Instrument Director : Prof. Chen-Yu Yeh

Operator : I-Chuan Chen

Note: This result is for academic use only, not to be used for any judicial or commercial advertising purpose.

Information of Submitted Sample

Web NO :	BSEA0001002021070024		
Department :	National Tsing Hua University department of chemistry		Date of
Supervisor :	Prof. Yi-Chou Tsai	Receive:	2021.07.22
User name :	Wei -Ting Kuo	completion:	2021.07.22

Results of Analysis

Sample code	*Weight(mg)*	*N %*	*C %*	*H %*	*O %*	*S %*	*Repeat*	*Charge*
WT4-P5E-8	2.982	6.20	54.30	6.09				$ 320

Instrumentation: *Elementar vario EL CUBE (CHN-OS Rapid, German), Accuracy: 0.1%, Precision: 0.2%*

	Standard sample	*N %*	*C %*	*H %*	*O %*	*S %*
★	*Acetanilide*	*10.36*	*71.09*	*6.71*		
	Benzoic acid				*26.20*	
	Sulfanilic acid	*8.09*	*41.60*	*4.07*		*18.50*
	Daily standard	*10.35*	*71.09*	*6.70*		

Figure *S*55. Elemental analysis report of **15**.

National Chung Hsing University

The Office of Research and Development, Instrument Center

Report on Elemental Analysis

Instrument Director : Prof. Chen-Yu Yeh

Operator : I-Chuan Chen

Note: This result is for academic use only, not to be used for any judicial or commercial advertising purpose.

Information of Submitted Sample

Web NO :	BSEA0001002022050040		
Department :	National Tsing Hua University Department of chemistry		Date of
Supervisor :	Prof. Yi-Chou Tsai	Receive:	2022.05.19
User name :	Wei-Ting Kuo	completion:	2022.05.19

Results of Analysis

Sample code	*Weight(mg)*	*N %*	*C %*	*H %*	*O %*	*S %*	*Repeat*	*Charge*
WT4-R2U-H-118	3.018	7.08	61.35	6.87				$ 320

Instrumentation: *Elementar vario EL CUBE (CHN-OS Rapid, German), Accuracy: 0.1%, Precision: 0.2%*

	Standard sample	*N %*	*C %*	*H %*	*O %*	*S %*
★	*Acetanilide*	*10.36*	*71.09*	*6.71*		
	Benzoic acid				*26.20*	
	Sulfanilic acid	*8.09*	*41.60*	*4.07*		*18.50*
	Daily standard	*10.42*	*71.14*	*6.72*		

Figure *S*56. Elemental analysis report of **16**.

5. Details of computational studies

Computational methods

Calculations were performed with the Gaussian 16 software package.[13] The molecular geometries were optimized without symmetry constraints at the BP86 level of density functional theory (DFT)[14–15] and stability of wavefunction was checked for optimized structure. Vibrational frequency calculations at the same level of theory have also been performed at 1 atm and 298.15 K to identify all the located stationary points as minima (zero imaginary frequency) or transition states (one imaginary frequency). The SCF convergence criterion was set to 10^{-8} in all cases. Intrinsic reaction coordinates (IRC) were calculated for the transition states to validate the expected reactants and products.[16–17] The 6-31G(d,p) Pople basis set was used to describe H, C, N, Cl, K and Ge atoms (named BS-I).[18–23] The basis set used for the single-point calculations comprised def2-TZVP for all atoms (named BS-II)[24] with the optimized structures at the BP86/BS-I level. Multiwfn 3.8 (dev) program[25] was used for the analyses of natural bond orbitals (NBO)[26] and electron localization function (ELF) [27] and the extended transition state method for energy decomposition analysis combined with the natural orbitals for chemical valence (ETS-NOCV).[28] Cartesian coordinates of the optimized geometries are listed in Table *S12*. To speed up the calculations for the intramolecular rearrangement of **5**, the bulky 2,6-$Et_2(C_6H_3)$ groups of the 2,6-diamidopyridyl ligands were replaced with the methyl groups.

Table *S*4. Comparisons of the experimental and calculated core structural metrics of **3**. Bond lengths and bond angles are respectively taken in units of (Å) and in units of (°).

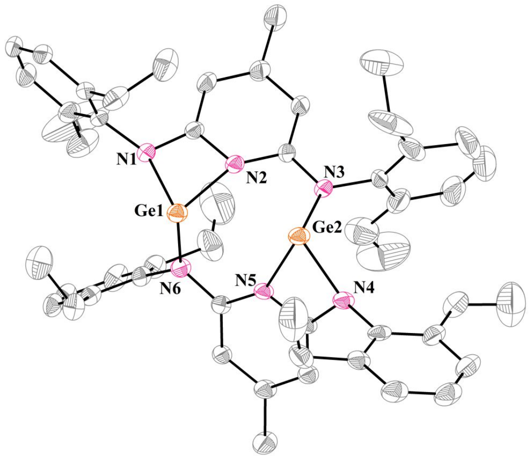

	Exp.	Cal.
Ge1−N1	1.976(5)	2.0238
Ge1−N2	2.084(6)	2.0667
Ge1−N6	1.965(6)	2.0246
Ge2−N3	1.956(6)	1.9935
Ge2−N4	1.970(6)	2.0051
Ge2−N5	2.091(5)	2.0466
N1−Ge1−N6	99.6(2)	101.288
N2−Ge1−N6	93.1(2)	97.361
N3−Ge2−N4	100.5(3)	100.468
N3−Ge2−N5	93.6(2)	97.750

Table *S*5. Comparisons of the experimental and calculated core structural metrics of **5**. Bond lengths and bond angles are respectively taken in units of (Å) and in units of (°).

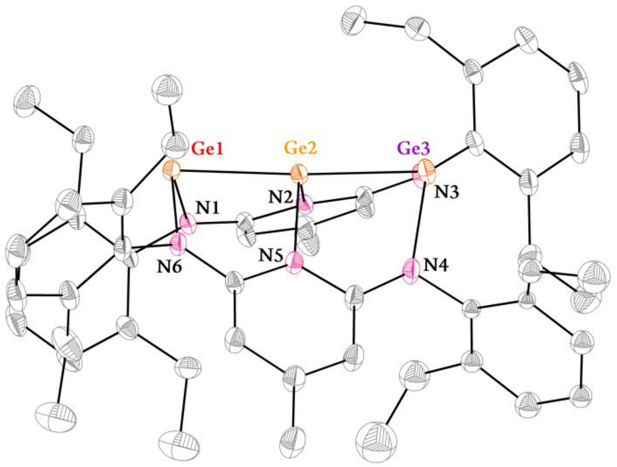

	Exp.	Cal.
Ge1−Ge2	2.5042(4)	2.4768
Ge2−Ge3	2.4986(4)	2.4793
Ge1−N1	2.023(2)	2.0328
Ge1−N6	1.965(2)	1.9928
Ge2−N2	1.968(2)	1.9579
Ge2−N5	1.965(2)	1.9586
Ge3−N3	1.966(3)	1.9912
Ge3−N4	2.026(3)	2.0376
Ge1−Ge2−Ge3	176.609(19)	174.231
N1−Ge1−Ge2	80.61(7)	81.308
N3−Ge3−Ge2	84.07(7)	83.524
N4−Ge3−Ge2	80.65(7)	81.058
N1−Ge1−N6	100.11(10)	98.135
N2−Ge2−N5	101.00(10)	99.093
N3−Ge3−N4	99.37(11)	98.121
N6−Ge1−Ge2	84.14(7)	83.718

Table *S*6. Comparisons of the experimental core structural metrics of **11** and calculated core structural metrics of $K_2Ge_6(\mu_3\text{-}\kappa^1{:}\kappa^1{:}\kappa^1\text{-DAP}^{Dipp})_2(\mu_4\text{-}\kappa^1{:}\kappa^1{:}\kappa^1{:}\eta^3\text{-DAP}^{Dipp})_2$ **(11m)**. Bond lengths and bond angles are respectively taken in units of (Å) and in units of (°).

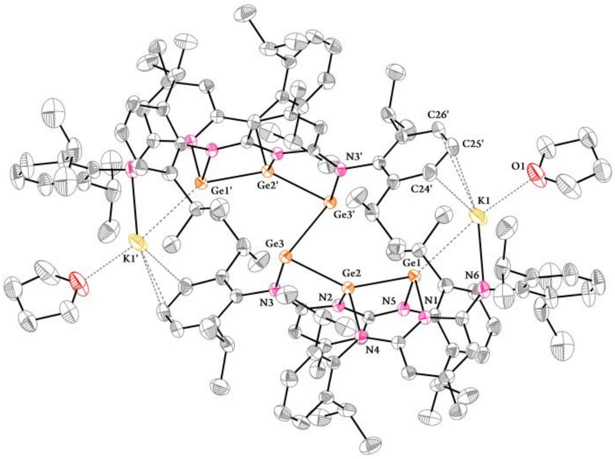

	Exp.	Cal.
Ge1−Ge2	2.4446(4)	2.4548
Ge1−N1	1.993(2)	2.0105
Ge1−N5	2.016(2)	2.0601
Ge1□□□K1	3.1870(8)	3.1318
Ge2−Ge3	2.4734(4)	2.4917
Ge2−N2	1.9671(19)	2.0088
Ge2−N4	1.960(2)	1.9860
Ge3−Ge3'	2.7634(5)	2.6716
Ge3−N3	2.059(2)	2.0585
Ge1−Ge2−Ge3	141.969(14)	139.242
Ge2−Ge3−Ge3'	77.609(12)	71.583
Ge2−Ge1−K1	136.68(2)	135.828
Ge3'−Ge3−N3	98.34(6)	95.683
N1−Ge1−N5	98.27(8)	96.309
N2−Ge2−N4	99.46(9)	99.252

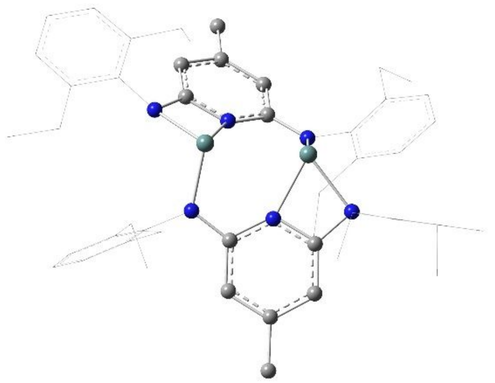

3

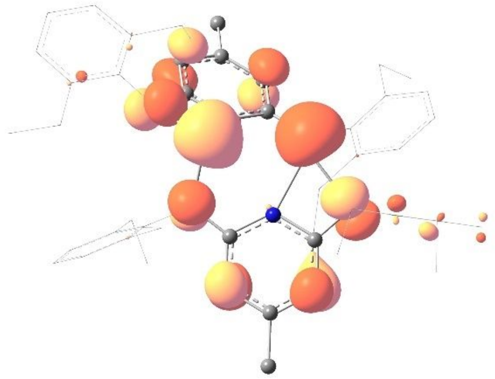

HOMO (−4.33 eV)

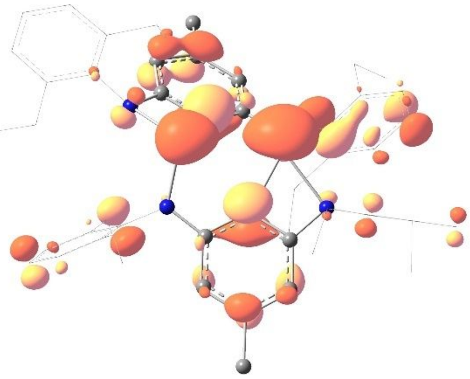

HOMO−13 (−6.53 eV)

Figure *S*57. Frontier molecular orbital of **3** (isovalue = 0.04 a.u.).

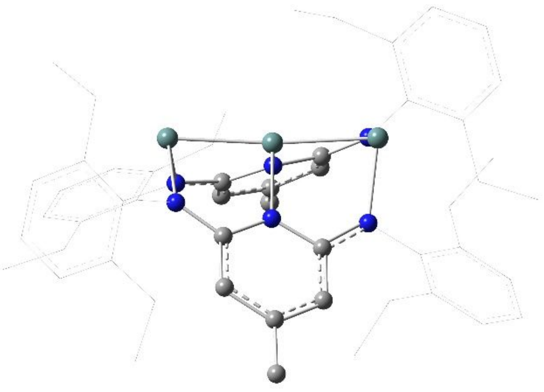

5

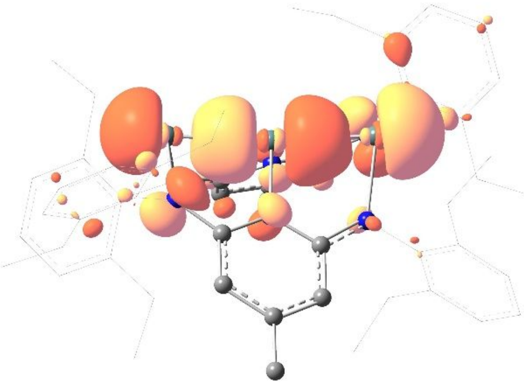

HOMO (−4.60 eV)

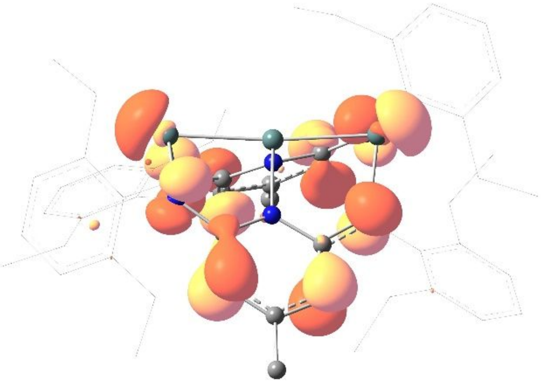

HOMO−2 (−4.77 eV)

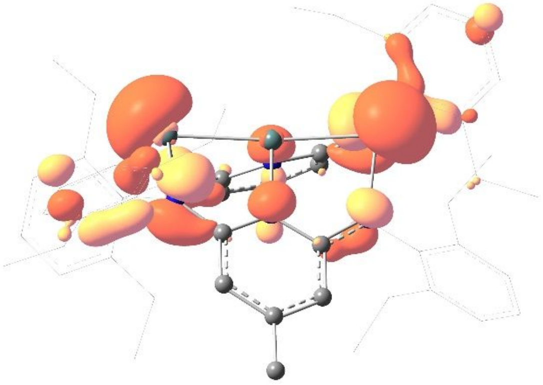

HOMO−3 (−5.41 eV)

Figure *S*58. Frontier molecular orbital of **5** (isovalue = 0.04 a.u.).

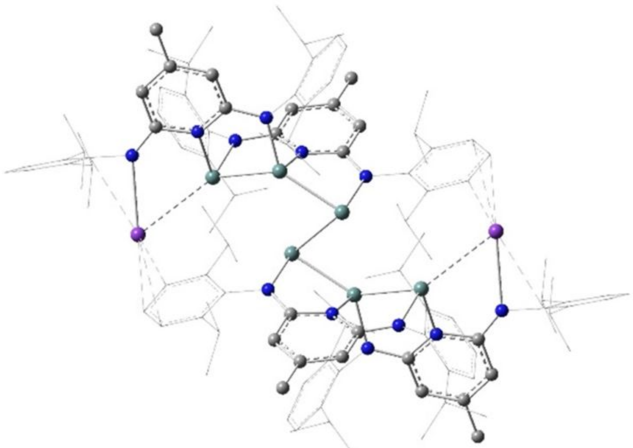

11m

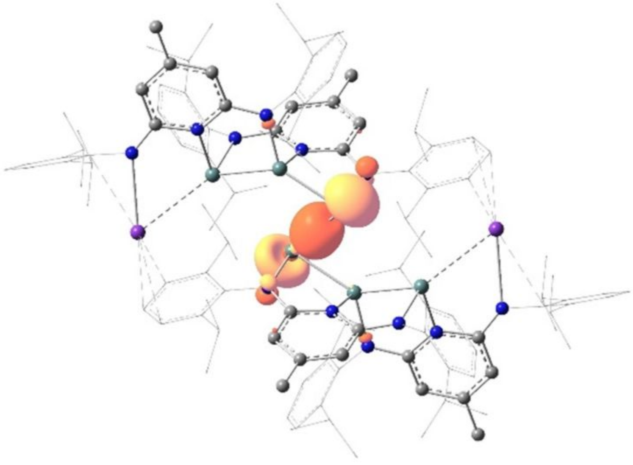

HOMO (−3.75 eV)

Figure *S*59. Frontier molecular orbital of **11m** (isovalue = 0.06 a.u.).

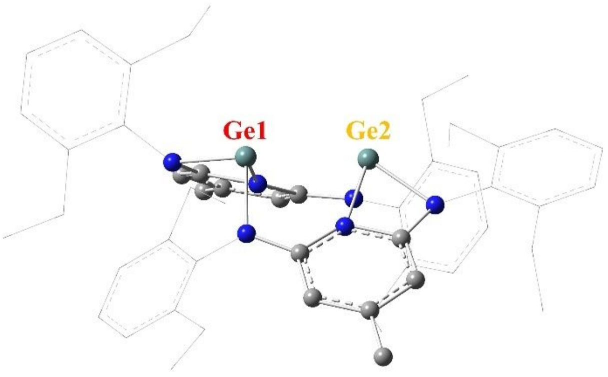

3

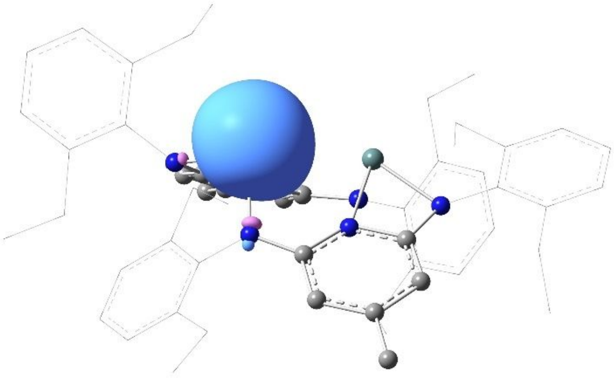

LP (1.85 e)
Ge1 ($sp^{0.18}$)

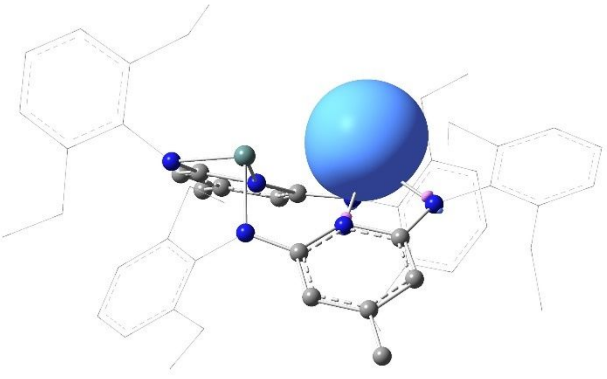

LP (1.87 e)
Ge2 ($sp^{0.16}$)

Figure *S*60. NBO analyses of **3** plotted with an isosurface of 0.05 a.u.

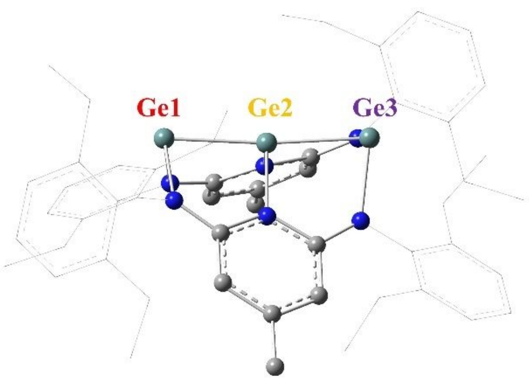

5

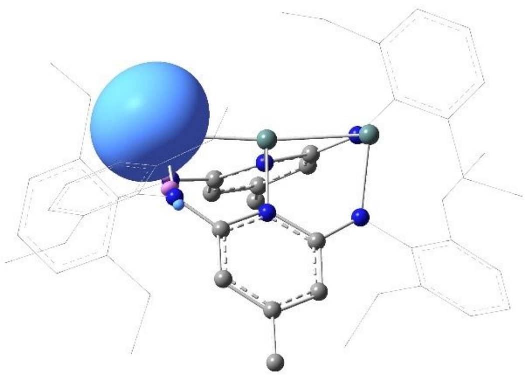

LP (1.94 e)
Ge1 (*sp*$^{0.24}$)

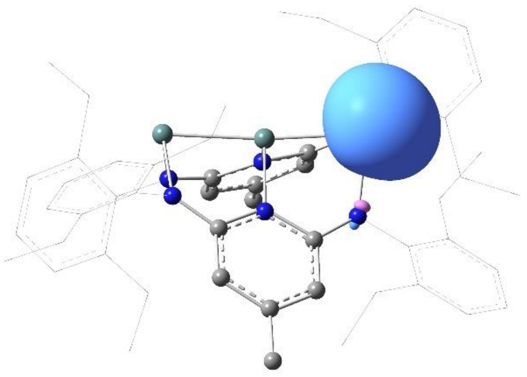

LP (1.94 e)
Ge3 (*sp*$^{0.24}$)

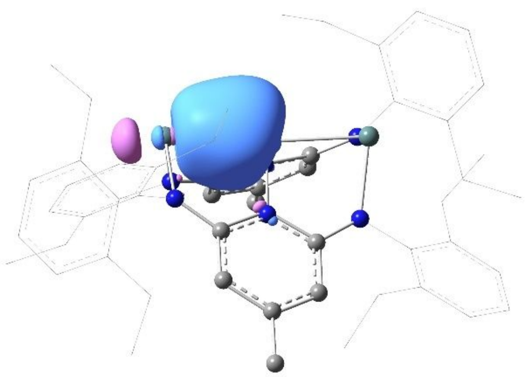

BD (1.90 e)
35% Ge1 (*sp*$^{9.61}$) + 65% Ge2 (*sp*$^{1.25}$)

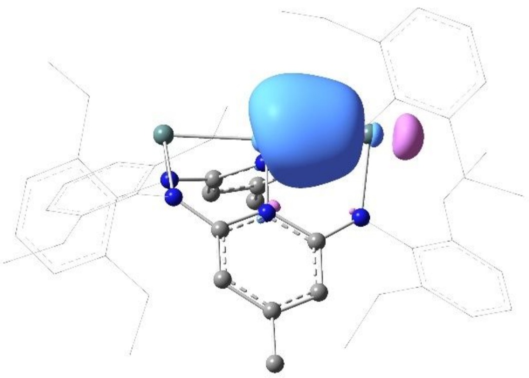

BD (1.90 e)
65% Ge2 (*sp*$^{1.27}$) + 35% Ge3 (*sp*$^{9.65}$)

Figure *S*61. NBO analyses of **5** plotted with an isosurface of 0.05 a.u.

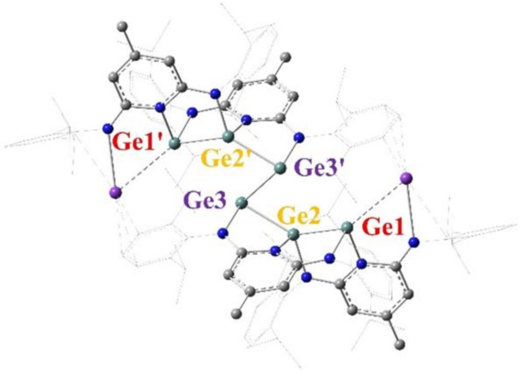

11m

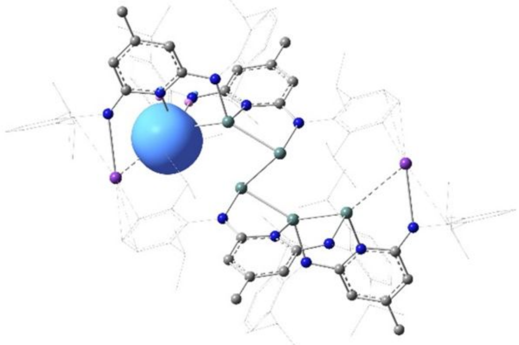

LP (1.91 e)
Ge1' (*sp*$^{0.30}$)

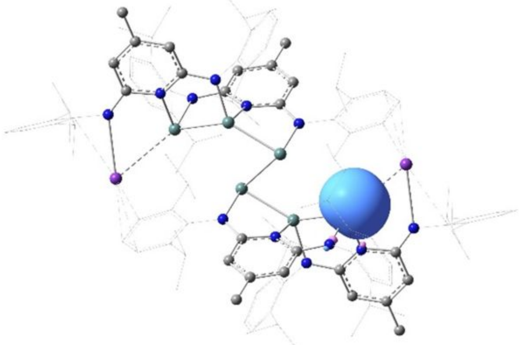

LP (1.91 e)
Ge1 (*sp*$^{0.30}$)

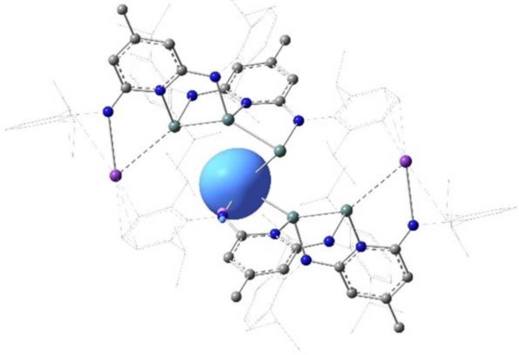

LP (1.88 e)
Ge3 (*sp*$^{0.24}$)

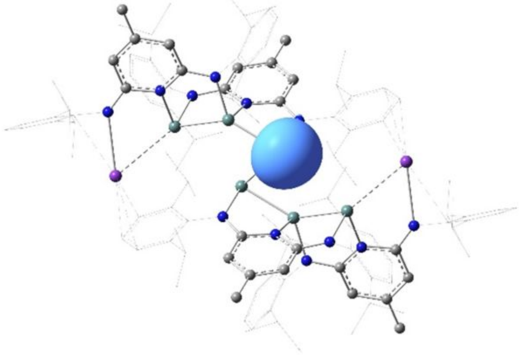

LP (1.88 e)
Ge3' (*sp*$^{0.24}$)

Figure *S*62. The first half of NBO analyses of **11m** plotted with an isosurface of 0.05 a.u.

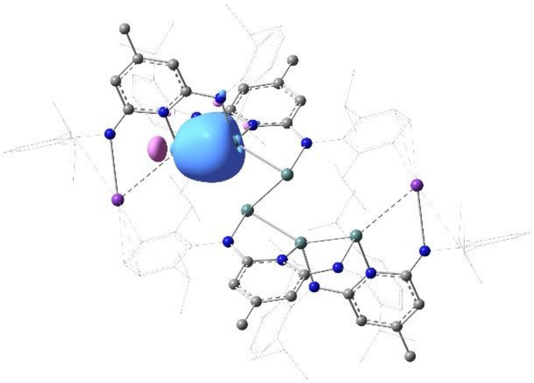

BD (1.88 e)
40% Ge1' (*sp*$^{7.14}$) + 60% Ge2' (*sp*$^{1.38}$)

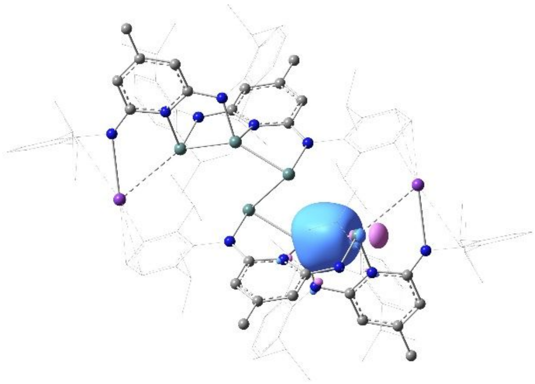

BD (1.88 e)
40% Ge1 (*sp*$^{7.14}$) + 60% Ge2 (*sp*$^{1.38}$)

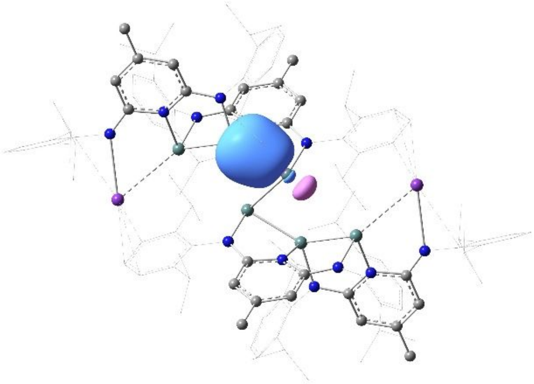

BD (1.89 e)
64% Ge2' (*sp*$^{1.09}$) + 36% Ge3' (*sp*$^{14.06}$)

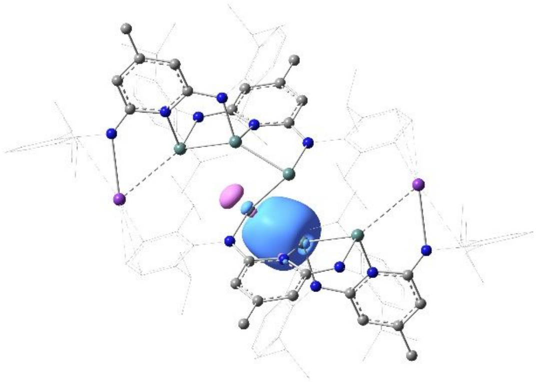

BD (1.89 e)
64% Ge2 (*sp*$^{1.09}$) + 36% Ge3 (*sp*$^{14.06}$)

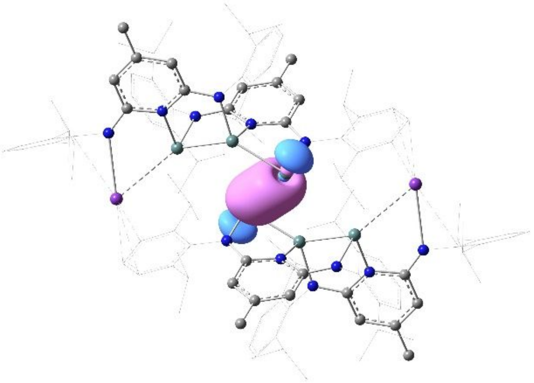

BD (1.70 e)
50% Ge3 (*sp*$^{10.32}$) + 50% Ge3' (*sp*$^{10.32}$)

Figure *S*63. The second half of NBO analyses of **11m** plotted with an isosurface of 0.05 a.u.

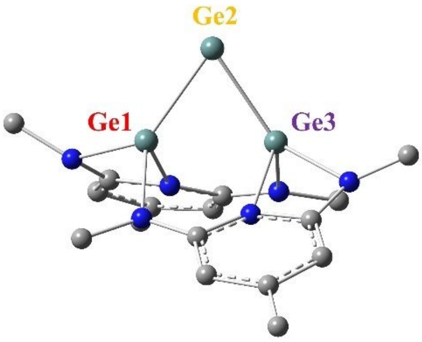

5A

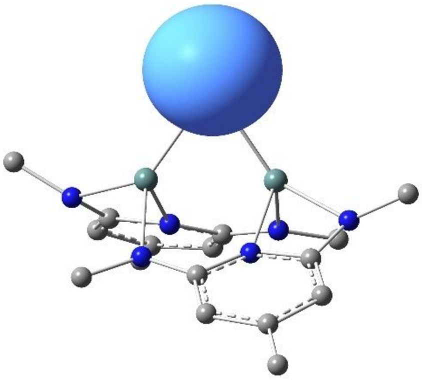

LP (1.88 e)
Ge2 (*sp*$^{0.12}$)

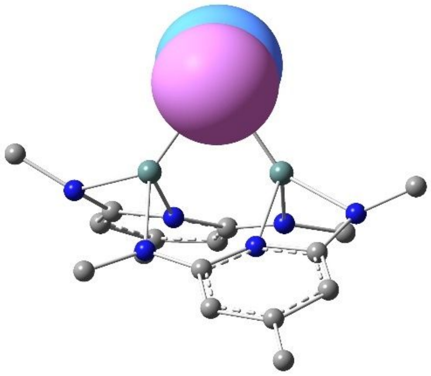

LP (1.25 e)
Ge2 (*p*)

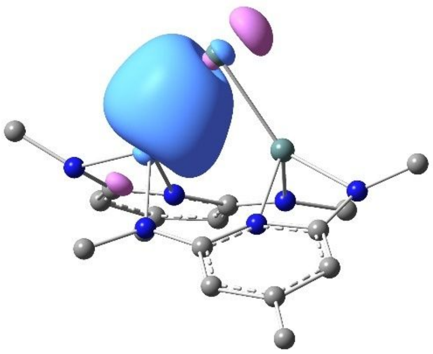

BD (1.69 e)
66% Ge1 (*sp*$^{1.67}$) + 34% Ge2 (*sp*$^{16.58}$)

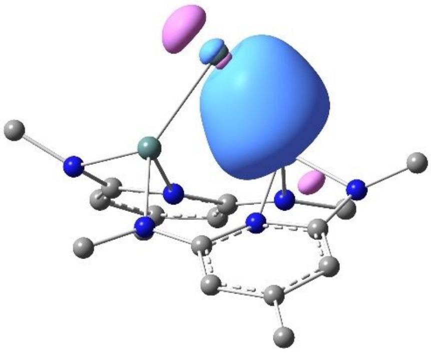

BD (1.69 e)
34% Ge2 (*sp*$^{16.58}$) + 66% Ge3 (*sp*$^{1.67}$)

Figure *S*64. NBO analyses of **5A** plotted with an isosurface of 0.05 a.u.

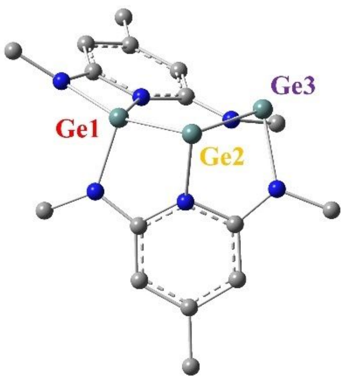

5B

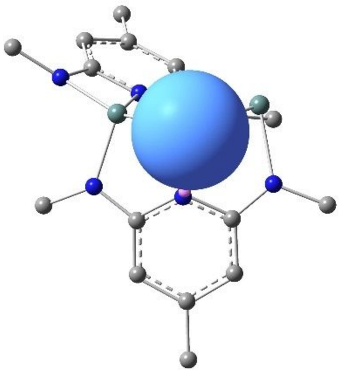

LP (1.89 e)
Ge2 ($sp^{0.24}$)

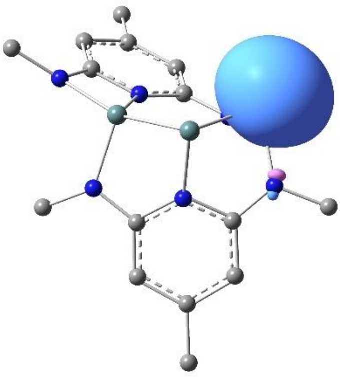

LP (1.93 e)
Ge3 ($sp^{0.23}$)

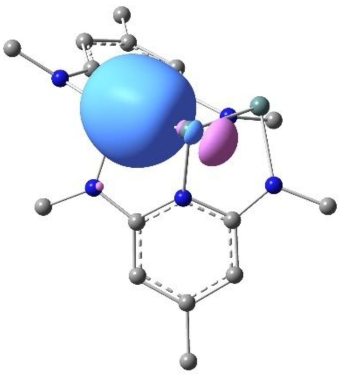

BD (1.81 e)
66% Ge1 ($sp^{1.72}$) + 34% Ge2 ($sp^{17.07}$)

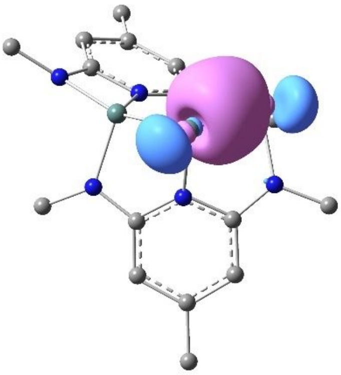

BD (1.81 e)
54% Ge2 ($sp^{9.52}$) + 46% Ge3 ($sp^{9.64}$)

Figure *S*65. NBO analyses of **5B** plotted with an isosurface of 0.05 a.u.

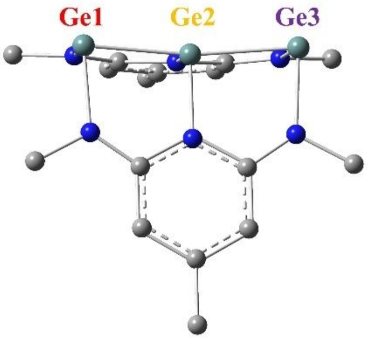

5C

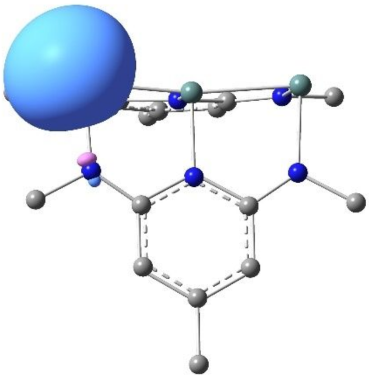

LP (1.95 e)
Ge1 (*sp*$^{0.27}$)

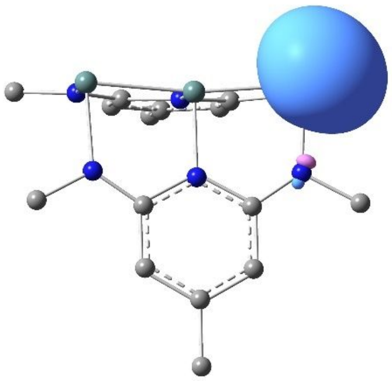

LP (1.95 e)
Ge3 (*sp*$^{0.27}$)

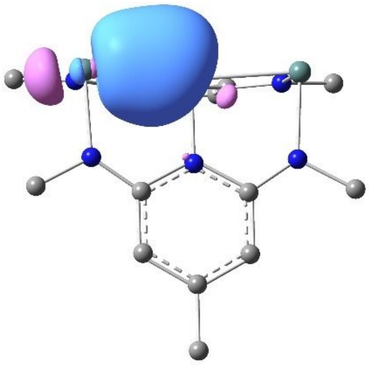

BD (1.88 e)
36% Ge1 (*sp*$^{12.21}$) + 64% Ge2 (*sp*$^{1.84}$)

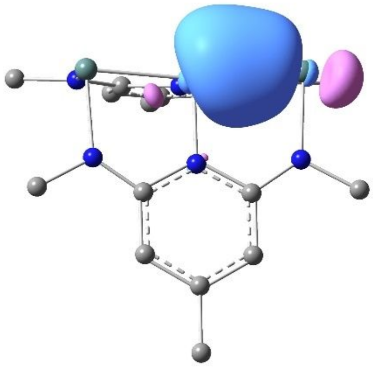

BD (1.88 e)
64% Ge2 (*sp*$^{1.84}$) + 36% Ge3 (*sp*$^{12.21}$)

Figure *S*66. NBO analyses of **5C** plotted with an isosurface of 0.05 a.u.

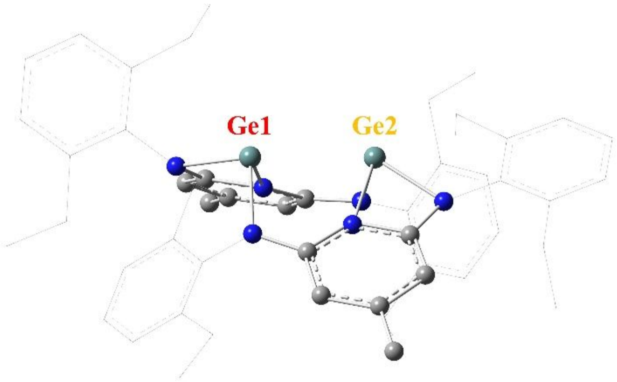

3

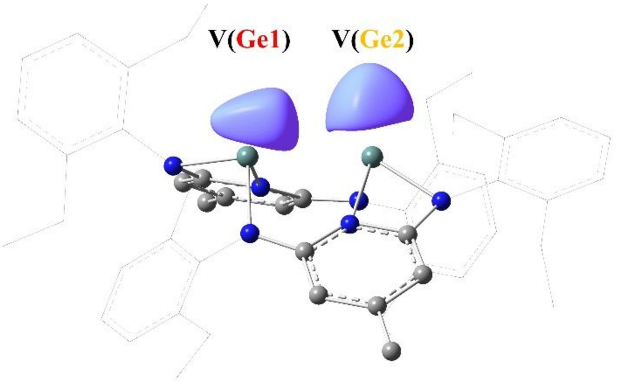

- **V(Ge1) = 2.20 e**
- **V(Ge2) = 2.21 e**

Figure *S*67. ELF plots of **3**. The ELF function of $\eta(\mathbf{r})$ = 0.7 is shown around Ge.

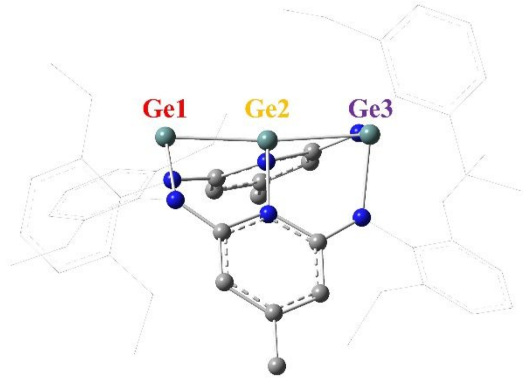

5

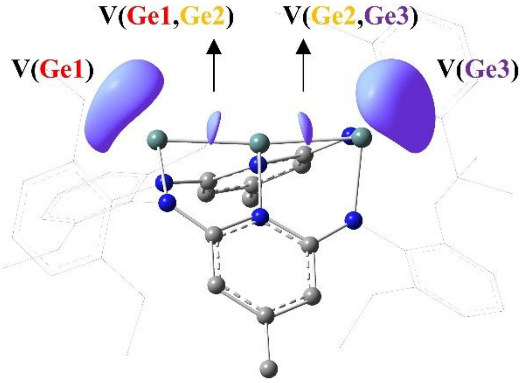

- **V(Ge1) = 2.26 e**
- **V(Ge3) = 2.26 e**
- **V(Ge1,Ge2) = 0.64 + 1.49 = 2.13 e**
- **V(Ge2,Ge3) = 1.50 + 0.64 = 2.14 e**

Figure *S*68. ELF plots of **5**. The ELF function of $\eta(\mathbf{r}) = 0.8$ is shown around Ge.

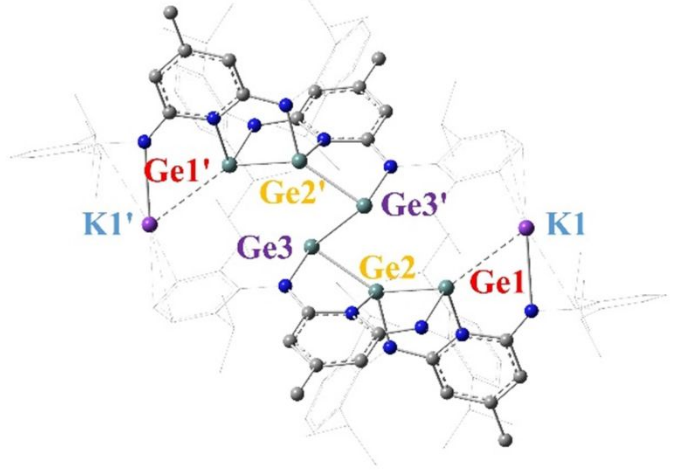

11m

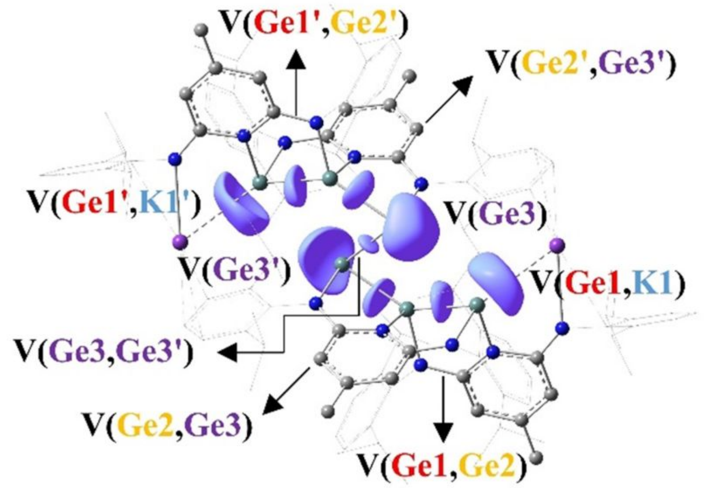

- **V(Ge3) = 2.27 e**
- **V(Ge3') = 2.27 e**
- **V(Ge1,Ge2) = 0.76 + 1.40 = 2.16 e**
- **V(Ge1,K1) = 2.20 + 0.03 = 2.23 e**
- **V(Ge2,Ge3) = 1.55 + 0.56 = 2.11 e**
- **V(Ge3,Ge3') = 0.84 + 0.84 = 1.68 e**
- **V(Ge1',Ge2') = 0.76 + 1.40 = 2.16 e**
- **V(Ge1',K1') = 2.20 + 0.03 = 2.23 e**
- **V(Ge2',Ge3') = 1.55 + 0.56 = 2.11 e**

Figure *S*69. ELF plots of **11m**. The ELF function of $\eta(\mathbf{r})$ = 0.7 is shown around Ge.

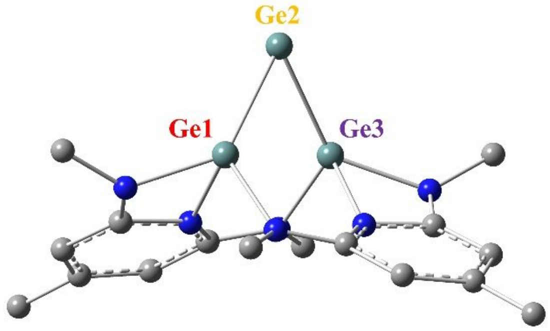

5A

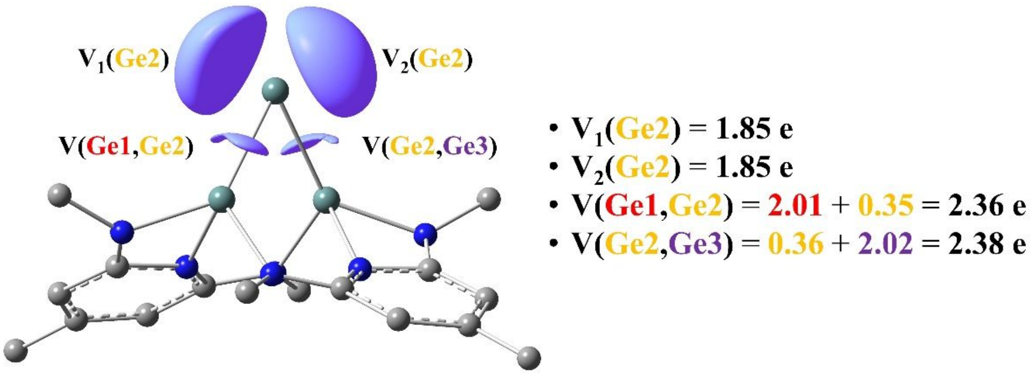

Figure *S*70. ELF plots of **5A**. V_1(Ge2) and V_2(Ge2) are drawn with the ELF function of $\eta(\mathbf{r})$ = 0.8, and V(Ge1,Ge2) and V(Ge2,Ge3) are drawn with the ELF function of $\eta(\mathbf{r})$ = 0.7.

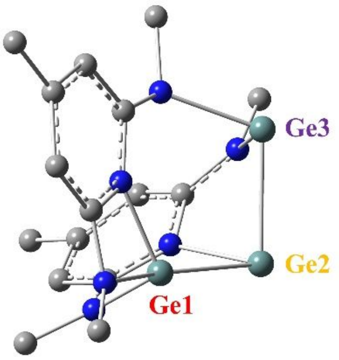

5B

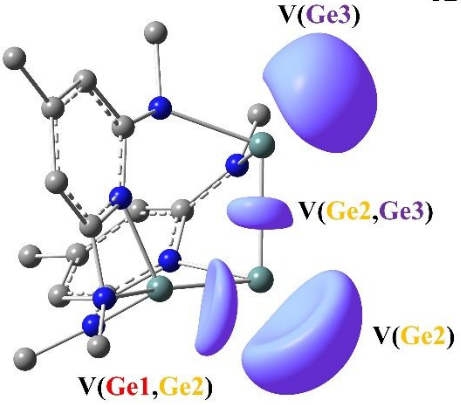

- **V(Ge2) = 2.28 e**
- **V(Ge3) = 1.85 e**
- **V(Ge1,Ge2) = 1.88 + 0.46 = 2.34 e**
- **V(Ge2,Ge3) = 0.97 + 0.90 = 1.87 e**

Figure *S*71. ELF plots of **5B**. V(Ge2) and V(Ge3) are drawn with the ELF function of $\eta(\mathbf{r}) = 0.8$, and V(Ge1,Ge2) and V(Ge2,Ge3) are drawn with the ELF function of $\eta(\mathbf{r}) = 0.7$.

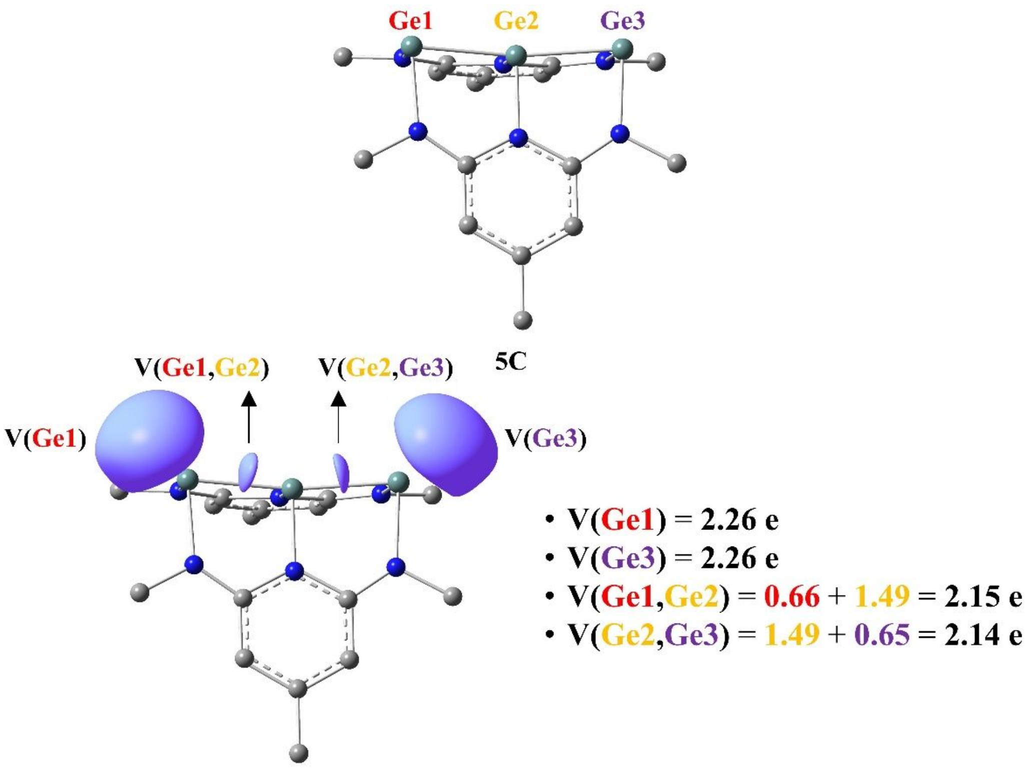

Figure *S*72. ELF plots of **5C**. The ELF function of $\eta(\mathbf{r}) = 0.8$ is shown around Ge.

Table *S*7. Deformation density plots of **5**. Main important ETS-NOCV deformation density contributions to the bonding between the Ge and the [Ge_2] fragment (isovalue = 0.005 a.u.). Green contour corresponds to depletion of electron density, whereas pink contour indicates accumulation of electron density. Charge flow from green to pink. All energies are given in kcal/mol.

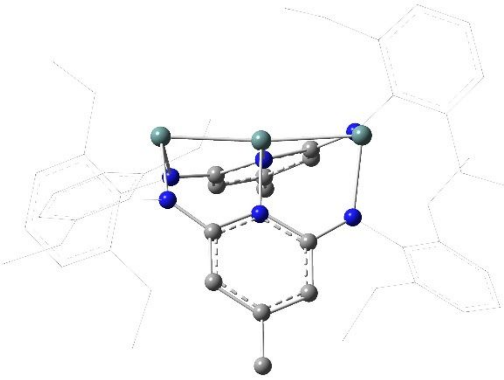

5

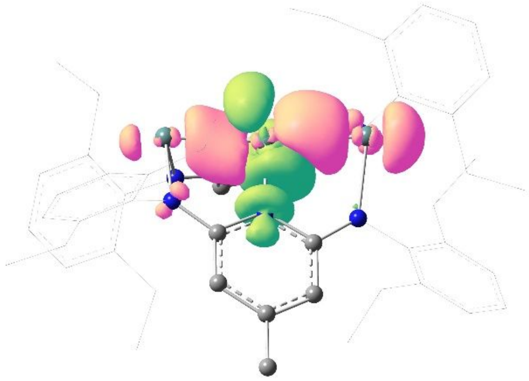

$\Delta\rho_1$

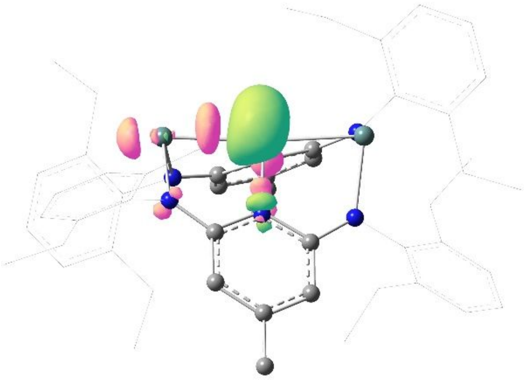

$\Delta\rho_2$

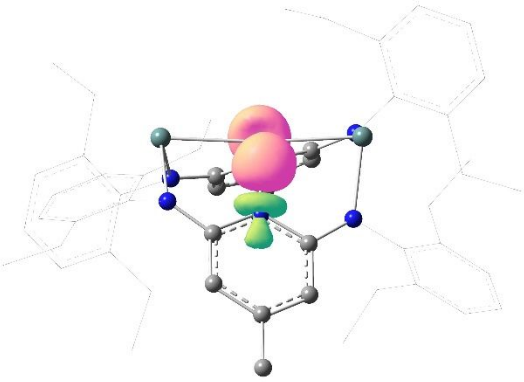

$\Delta\rho_3$

ΔE_{orb}	$\Delta\rho_1$	$\Delta\rho_2$	$\Delta\rho_3$
−450.65	−298.96 (66.34%)	−58.22 (12.92%)	−38.74 (8.60%)

[Ge_2] = $Ge_2(\mu\text{-}\kappa^1{:}\kappa^2\text{-DAP}^{Dep})_2$

Table *S*8. Deformation density plots of **5A**. Main important ETS-NOCV deformation density contributions to the bonding between the Ge and the [Ge_2] fragment (isovalue = 0.008 a.u.). Green contour corresponds to depletion of electron density, whereas pink contour indicates accumulation of electron density. Charge flow from green to pink. All energies are given in kcal/mol.

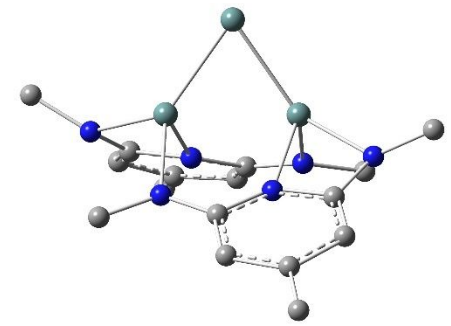

5A

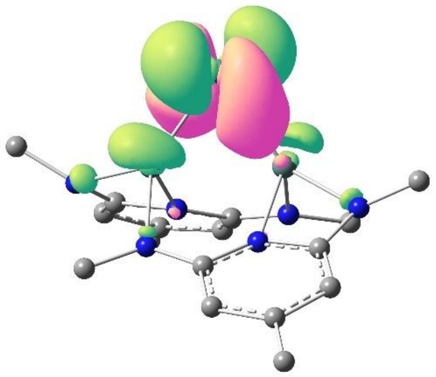

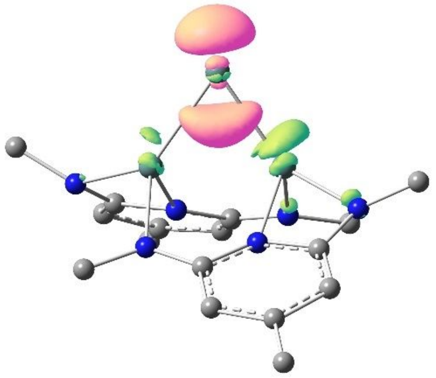

$\Delta\rho_1$ **$\Delta\rho_2$**

ΔE_{orb}	$\Delta\rho_1$	$\Delta\rho_2$
−180.24	−109.26 (60.62%)	−49.08 (27.23%)

[Ge_2] = $Ge_2(\mu$-κ^1:κ^2-DAP$^{Me})_2$

Table *S*9. Deformation density plots of **5B**. Main important ETS-NOCV deformation density contributions to the bonding between the Ge and the $[Ge_2]$ fragment (isovalue = 0.005 a.u.). Green contour corresponds to depletion of electron density, whereas pink contour indicates accumulation of electron density. Charge flow from green to pink. All energies are given in kcal/mol.

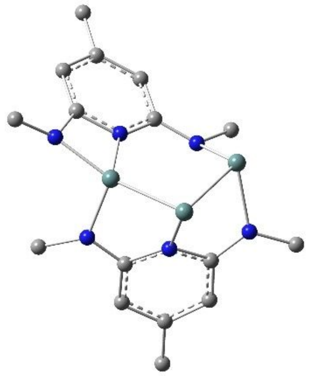

5B

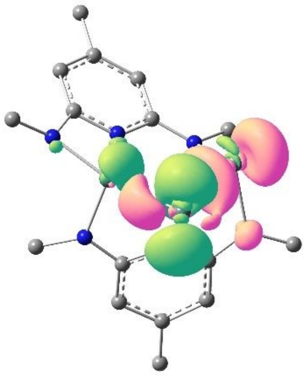

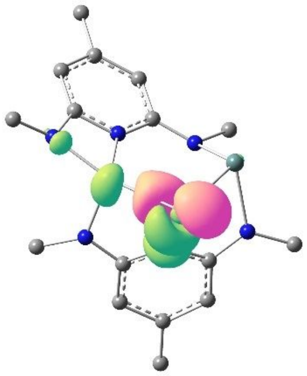

$\Delta\rho_1$ **$\Delta\rho_2$**

ΔE_{orb}	$\Delta\rho_1$	$\Delta\rho_2$
−249.31	−150.19 (60.24%)	−40.66 (16.31%)

$[Ge_2] = Ge_2(\mu\text{-}\kappa^1\text{:}\kappa^2\text{-DAP}^{Me})_2$

Table *S*10. Deformation density plots of **5C**. Main important ETS-NOCV deformation density contributions to the bonding between the Ge and the [Ge_2] fragment (isovalue = 0.005 a.u.). Green contour corresponds to depletion of electron density, whereas pink contour indicates accumulation of electron density. Charge flow from green to pink. All energies are given in kcal/mol.

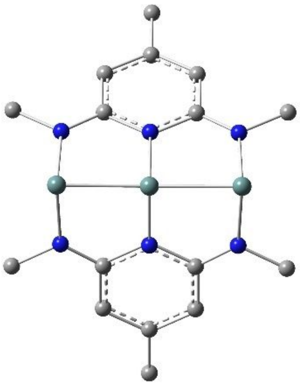

5C

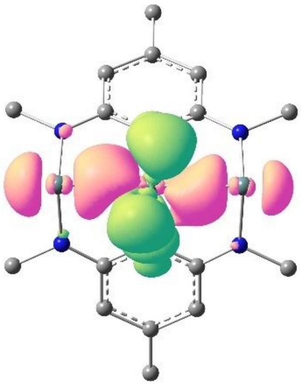

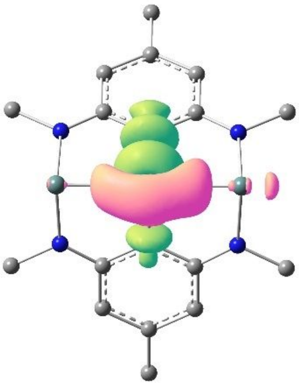

$\Delta\rho_1$ **$\Delta\rho_2$**

ΔE_{orb}	$\Delta\rho_1$	$\Delta\rho_2$
−505.03	−283.27 (56.09%)	−152.74 (30.24%)

[Ge_2] = $Ge_2(\mu\text{-}\kappa^1{:}\kappa^2\text{-DAP}^{Me})_2$

Table *S*11. Comparisons of the calculated structural metrics of **5A**, **TS$_{5A5B}$**, **5B**, **TS$_{5B5C}$** and **5C**. Bond lengths and bond angles are respectively taken in units of (Å) and in units of (°).

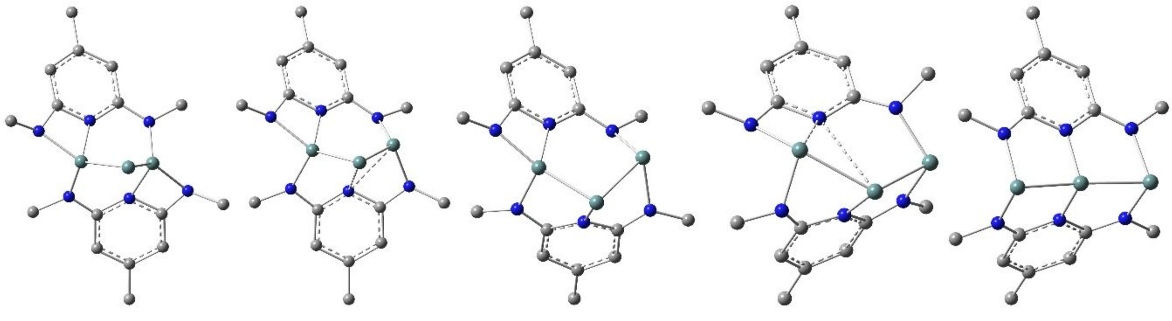

5A **TS$_{5A5B}$** **5B** **TS$_{5B5C}$** **5C**

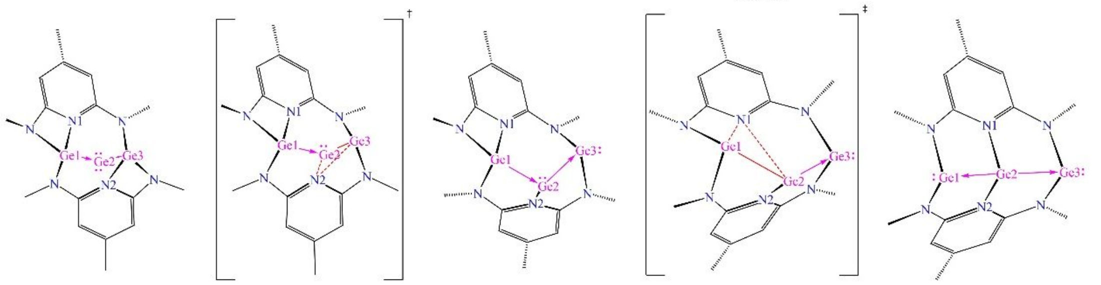

Complex	**5A**	**TS$_{5A5B}$**	**5B**	**TS$_{5B5C}$**	**5C**
Ge1−Ge2	2.4227	2.4422	2.4003	2.6512	2.4507
Ge1−N1	1.9855	1.9222	1.9428	2.1327	3.2188
Ge2−Ge3	2.4228	2.4596	2.5237	2.5001	2.4507
Ge2−N1	3.6535	3.7052	3.6596	3.2673	1.9476
Ge2−N2	3.6530	3.3258	2.0826	1.9764	1.9478
Ge3−N2	1.9858	2.6666	3.0361	3.1053	3.2187
Ge1−Ge2−Ge3	68.596	77.3147	93.342	117.776	169.491
N1−Ge2−N2	53.806	53.916	69.474	67.086	99.814

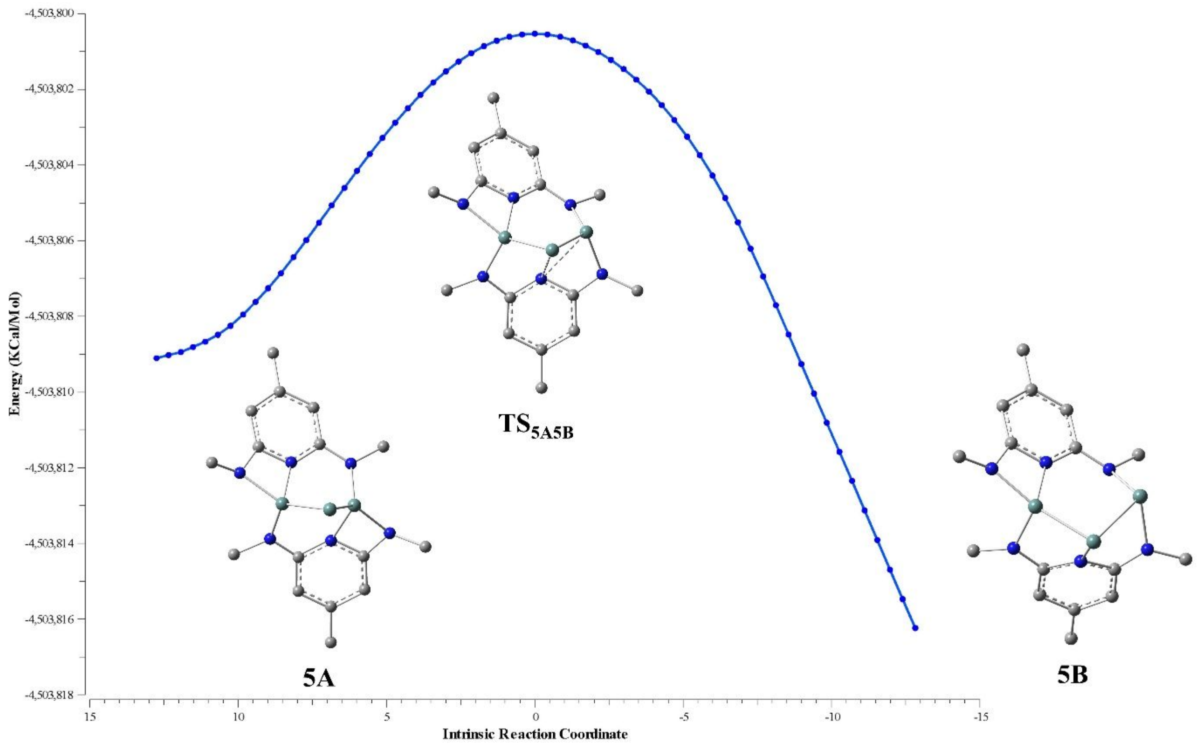

Figure *S*73. IRC analysis results for the $\mathbf{TS_{5A5B}}$ at 1 atm and 298.15 K.

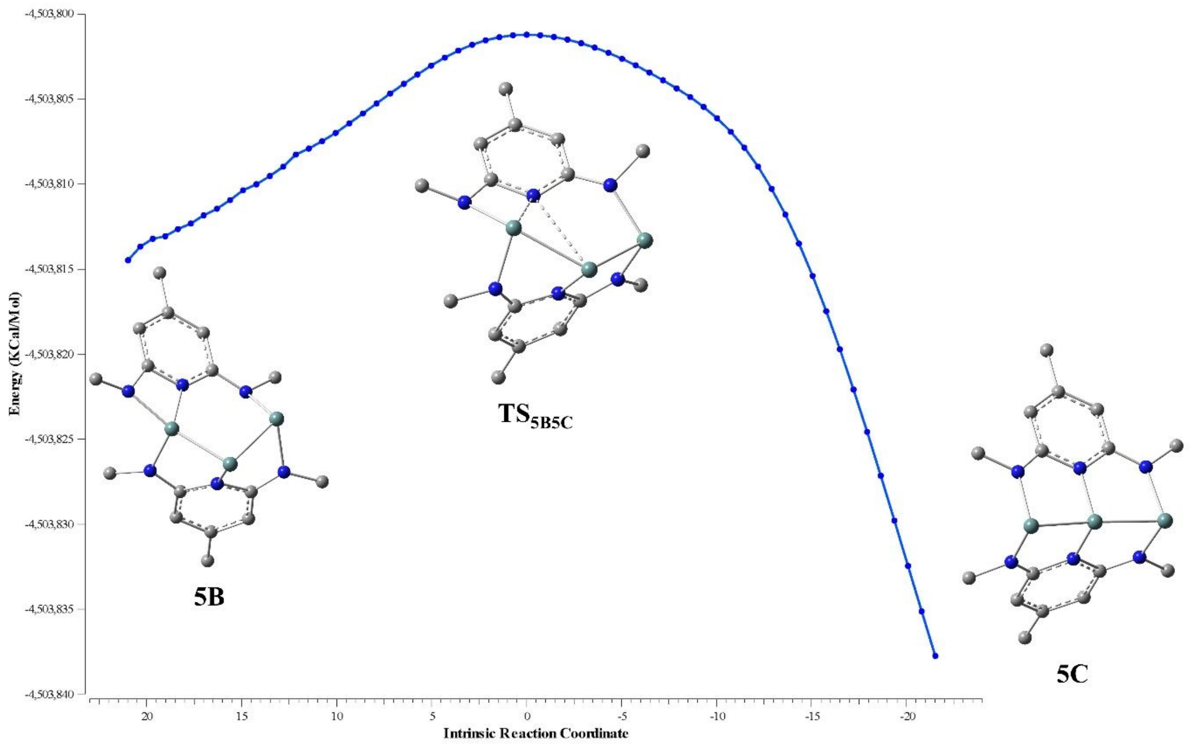

Figure *S*74. IRC analysis results for the $\mathbf{TS_{5A5C}}$ at 1 atm and 298.15 K.

Table *S*12. Cartesian coordinates data sets and thermodynamic data for DFT optimized complexes in at 1 atm and 298.15 K.

3

**Electronic energy (BP86)**	**−6502.850360**
**Zero-point correction**	1.000724
**Thermal correction to energy**	1.064573
**Thermal correction to enthalpy**	1.065518
**Thermal correction to Gibbs free energy**	0.896476

C	15.88955721	8.83951721	2.66495130
C	16.87443857	7.83202051	2.86104684
C	16.03282978	9.79265704	1.61302196
N	14.72681869	8.83321460	3.49470241
C	16.73957219	6.81773124	3.97773530
C	17.99915813	7.80261588	2.01641543
C	17.17682352	9.72021585	0.79392016
C	15.01570279	10.88806347	1.32078646
C	14.44817363	9.85187081	4.37577114
Ge	12.78472464	8.53195230	3.01184488
C	17.53786284	7.20768454	5.23905277
C	18.15692662	8.73955198	0.98791320
C	15.52053836	12.30101906	1.68451660
C	15.28853002	10.72387347	5.08222201
N	13.07799080	9.90876715	4.52503594
N	12.32512488	6.94876468	4.18712878
C	14.68406640	11.61431865	5.99982345
C	12.46554947	10.80461232	5.35393542
C	11.00003176	6.85616400	4.51750764

C	13.11885895	5.75164006	4.19369528
C	15.55541067	12.53521476	6.82584463
C	13.28861139	11.65094920	6.14747052
N	11.10197803	10.81785054	5.31624630
C	10.30247820	5.67071341	4.88137683
N	10.28399615	8.01516590	4.45669646
C	13.39026666	5.06575692	2.97826856
C	13.61035084	5.25211073	5.43564753
C	10.36586623	11.66174876	6.20811957
Ge	10.31070489	9.92418425	3.71956937
C	8.91319869	5.70309165	5.08405076
C	8.91881532	8.06746168	4.64830584
C	12.82449236	5.50066685	1.63423614
C	14.19627103	3.90890220	3.02484249
C	14.40004814	4.08903541	5.42872319
C	13.29539439	5.94621430	6.74966460
C	9.89668461	12.92938423	5.77880040
C	10.08127772	11.17049690	7.51301929
N	8.52732740	9.37692883	4.45481786
C	8.19000114	6.91114526	4.95231447
C	8.17363797	4.43046822	5.43495664
C	13.87655023	6.09869487	0.67733345
C	14.70453292	3.42244034	4.23294297
C	13.99533518	7.31034382	6.89867742
C	10.18003029	13.48337794	4.39118458
C	9.16664845	13.71335795	6.69676132

C	9.36771035	11.99504872	8.39916030
C	10.54821420	9.77201436	7.87871384
C	7.22458832	9.69209870	3.95297100
C	11.34951598	14.49116939	4.37251233
C	8.91333225	13.26111952	7.99620638
C	9.88350894	9.13830444	9.10664096
C	6.25875702	10.21126508	4.85855949
C	6.91085109	9.49544130	2.58061764
C	6.65147274	10.41218855	6.31230897
C	4.97973961	10.52889102	4.37083669
C	5.61274363	9.82573744	2.13880327
C	7.91039595	8.96876987	1.55933336
C	5.58929520	11.04485472	7.21875176
C	4.65462367	10.33688118	3.01937436
C	8.46202719	10.06148183	0.61730542
H	10.85260970	4.72986986	4.94274470
H	7.10673652	6.95604251	5.08372895
H	8.84902213	3.56155541	5.46693968
H	7.68359793	4.52069209	6.42057143
H	7.37694855	4.22233467	4.69944011
H	12.20278328	6.09157342	6.83581783
H	13.58426277	5.27555170	7.57917846
H	15.09110785	7.19525290	6.93248412
H	13.73940167	7.93955631	6.03503723
H	13.67408924	7.82378593	7.82071760
H	14.77707084	3.70246144	6.38269790

H	15.32162508	2.51795621	4.24770952
H	14.40598635	3.37506442	2.09043981
H	12.36922789	4.61634899	1.14955596
H	12.00395329	6.22513109	1.77856744
H	14.67756922	5.37254970	0.45770940
H	13.41078709	6.39525110	-0.27775791
H	14.36034111	6.99229009	1.10911290
H	15.67341709	6.71412278	4.23131168
H	17.08864364	5.83343266	3.61721621
H	18.61179875	7.32806507	5.01301124
H	17.17421425	8.16170349	5.65718653
H	17.43950795	6.43393745	6.01991335
H	18.75463994	7.02282631	2.16959162
H	19.03235068	8.69816608	0.33115710
H	17.28605120	10.44400999	-0.02277842
H	14.77642471	10.85939466	0.24110723
H	14.06141825	10.70237692	1.84358837
H	16.45443418	12.54128585	1.14815297
H	14.77016777	13.06515614	1.41925323
H	15.72109649	12.37786517	2.76567563
H	16.37189497	10.67243665	4.95376228
H	12.81430644	12.35344396	6.83573141
H	16.18380659	13.17072541	6.17761277
H	14.95490017	13.19174470	7.47431479
H	16.24184918	11.95416607	7.46673618
H	12.28647023	14.01046758	4.69907605

H	11.50500963	14.89362398	3.35700741
H	11.15158294	15.34014655	5.04931833
H	10.40343220	12.66272774	3.68456584
H	9.26523830	13.97755440	4.01585928
H	8.79505479	14.69390614	6.37654648
H	8.34815581	13.88724337	8.69464314
H	9.14476162	11.63723187	9.40918366
H	10.39377657	9.14711355	6.98144000
H	11.64725914	9.78591336	8.02380911
H	10.12074900	9.68499254	10.03586785
H	8.78522093	9.11066827	9.00325545
H	10.23533608	8.10197753	9.23909842
H	6.95793976	9.43480574	6.73336467
H	7.57352436	11.01965980	6.33546984
H	5.98966298	11.17182350	8.23778108
H	5.28606615	12.04231557	6.85626513
H	4.68057132	10.42170993	7.29151670
H	3.65119880	10.58431043	2.65673689
H	4.22554034	10.92888875	5.05515660
H	5.35776216	9.67092150	1.08351634
H	8.75674754	8.45379012	2.04767089
H	7.40603954	8.19745185	0.94712630
H	9.14294723	9.62065136	-0.13081078
H	7.64634544	10.57582883	0.08125637
H	9.03028143	10.82140343	1.18133719

5

**Electronic energy (BP86)**	**−8580.176125**
**Zero-point correction**	1.003600
**Thermal correction to energy**	1.068476
**Thermal correction to enthalpy**	1.069420
**Thermal correction to Gibbs free energy**	0.900349

C	4.54784415	3.42501464	3.10515298
C	5.09626039	3.71860546	1.82450494
C	4.48944331	2.09451702	3.59551255
N	4.05157795	4.52454850	3.88748001
C	5.12960103	5.16796103	1.37052357
C	5.58486022	2.65902074	1.04242443
C	4.99644973	1.06442562	2.77518706
C	3.88693872	1.73868982	4.94695799
C	2.76041549	4.90779039	3.65999332
Ge	5.10496402	4.88684844	5.58792696
C	6.01424803	5.47878918	0.15770824
C	5.53604785	1.33711999	1.51413081
C	4.89994831	1.11219614	5.92658209
C	1.87864672	4.27576018	2.75039361
N	2.29545725	5.97437839	4.40536097
Ge	3.49275798	6.76098153	5.73989862
N	6.32852052	6.28585889	4.86898933
C	0.53545605	4.67223583	2.68469771
C	0.96659784	6.35862261	4.38816949
Ge	1.88534396	8.60581857	6.13953000
N	4.60550670	7.85143856	4.55296111

C	5.92587786	7.49314196	4.35528537
C	7.68949270	6.14951319	5.32004003
C	-0.40731880	4.01030449	1.70490271
C	0.07079428	5.70020393	3.52328276
N	0.60592963	7.38849843	5.21963171
N	2.81541164	9.34880676	4.48580749
C	4.09952160	9.06182193	4.11911290
C	6.77558333	8.35756739	3.63832540
C	8.09324193	6.83434592	6.50195683
C	8.57082725	5.26587374	4.64554605
C	-0.71966626	7.40103559	5.78289904
C	2.26105284	10.57959060	3.99207528
C	4.93636171	9.91800600	3.36247024
C	6.27551104	9.57045884	3.13479141
C	7.09081358	7.68862321	7.26895417
C	9.41588908	6.66846139	6.94949716
C	9.88018650	5.11919320	5.14065345
C	8.07766999	4.51085443	3.42491643
C	-1.04830547	6.48832078	6.82276773
C	-1.64904819	8.39443782	5.36777827
C	2.37812245	11.78217791	4.74259927
C	1.58928676	10.56101965	2.73879948
C	7.18897786	10.51189750	2.38225597
C	7.59949251	8.32383774	8.56982790
C	10.30590044	5.82281885	6.27355451
C	9.06853470	3.53093376	2.78619230

C	-0.07428120	5.45057837	7.35959138
C	-2.33602831	6.55325496	7.39133826
C	-2.91875408	8.42483053	5.97016730
C	-1.24126540	9.38600766	4.29458315
C	1.81868751	12.95884501	4.20482562
C	3.09275728	11.84856616	6.08490224
C	1.04896501	11.76204616	2.24360637
C	1.45788051	9.27315272	1.94623696
C	-0.35448113	4.01864992	6.85571293
C	-3.26832241	7.50597567	6.96993955
C	-2.28476030	10.44096082	3.91089691
C	2.16603806	12.24460876	7.25254978
C	1.16257031	12.95675455	2.96769978
C	2.42738380	9.17541787	0.74983933
H	4.53592775	10.86789398	3.00582571
H	7.88981238	9.95838350	1.73581362
H	6.61867560	11.21766522	1.75801294
H	7.79857571	11.10562660	3.08745705
H	7.81542091	8.06310063	3.48307986
H	7.15499765	3.96521545	3.70186178
H	7.74599100	5.24708687	2.66832210
H	9.39099452	2.75430176	3.50090128
H	8.59238757	3.01935442	1.93420232
H	9.97286163	4.04215616	2.41194078
H	8.43672733	9.01901106	8.38600141
H	6.79211445	8.89824800	9.05299737

H	7.94424440	7.56228812	9.28994167
H	6.70848439	8.48828978	6.60643177
H	6.20774460	7.05862415	7.50058288
H	9.75127691	7.19214404	7.84940684
H	11.33073389	5.70223671	6.64041923
H	10.57683464	4.44901366	4.62865520
H	1.91113378	13.89447542	4.76838357
H	0.73911465	13.88474274	2.56918970
H	0.53099170	11.75307740	1.27715844
H	1.61725180	8.42988989	2.63681786
H	0.42135334	9.19519380	1.56837119
H	3.47677446	9.21324806	1.08477861
H	2.27608321	8.22972849	0.20193055
H	2.26949486	10.00667351	0.04078669
H	3.56377842	10.87749945	6.31408922
H	3.91731970	12.58347562	6.01014944
H	1.70569952	13.23401443	7.08973342
H	1.35589524	11.50577540	7.36916706
H	2.73090259	12.28715340	8.19940949
H	-0.97510954	6.01358116	3.51272072
H	2.25001334	3.44826185	2.14421910
H	-0.03668289	3.02201336	1.39035433
H	-1.41365898	3.88670306	2.13784116
H	-0.51784382	4.62926752	0.79593683
H	3.44215282	2.63493056	5.41235733
H	3.05325468	1.02873119	4.78377043

H	5.72109457	1.81778579	6.13577173
H	4.41082192	0.86145825	6.88330529
H	5.34007420	0.18572825	5.51997547
H	4.09376140	5.49944127	1.15690636
H	5.43669149	5.76180026	2.24975042
H	7.05894293	5.16204626	0.32169110
H	5.65282849	4.97704977	-0.75682541
H	6.01883599	6.56260091	-0.04298971
H	6.00862700	2.86624114	0.05478632
H	5.91706288	0.52002537	0.89233657
H	4.95050053	0.02993601	3.13458493
H	0.96402409	5.73095870	7.11130057
H	-0.13709389	5.45878438	8.46362028
H	0.35412778	3.30003030	7.30179496
H	-1.37587993	3.69942535	7.12515364
H	-0.26271998	3.95896663	5.75905907
H	-2.59402197	5.85271657	8.19457826
H	-4.26176332	7.54592707	7.42906526
H	-3.64559068	9.17871988	5.65401839
H	-0.31394540	9.89376439	4.62278442
H	-0.93849695	8.81950833	3.39414628
H	-1.87034816	11.11694242	3.14557927
H	-3.20154232	9.98420084	3.49843837
H	-2.57599057	11.06059716	4.77646374

11m

**Electronic energy (BP86)**	**−18989.500566**
**Zero-point correction**	2.462306
**Thermal correction to energy**	2.589520
**Thermal correction to enthalpy**	2.590464
**Thermal correction to Gibbs free energy**	2.304231

C	8.54679535	4.98250798	5.11278340
C	8.49983828	4.77725680	3.70537163
C	7.86122945	4.08820920	5.98945666
N	9.26795959	6.10545125	5.66269299
C	9.08379374	5.78048514	2.71645882
C	7.78459131	3.67311910	3.20126724
C	7.16268389	2.99954054	5.43134331
C	7.78312644	4.28865360	7.50331090
C	8.52926408	7.17472627	6.09396197
Ge	11.17000323	5.78794088	6.23133987
C	9.93890215	5.11940597	1.61723873
C	7.93448060	6.57855603	2.05789296
C	7.12466552	2.78106849	4.05055593
C	8.11160221	2.98707772	8.26357779
C	6.36770631	4.76755835	7.90945369
C	7.17774449	7.37785504	5.73519535
N	9.16112056	8.08251164	6.92642705
Ge	11.16904558	8.13579582	6.94807140
N	12.04554726	6.55664201	4.53237713
C	6.47860715	8.48147991	6.24590611
C	8.44881014	9.08116505	7.55899288

Ge	11.08015110	9.46663231	9.05268812
N	11.49084954	8.79623279	5.10297939
C	12.02247169	7.89894222	4.18261750
C	12.71106052	5.60908019	3.73242078
C	5.03531619	8.71557051	5.85838924
C	7.10761924	9.32803338	7.16635887
N	9.11570122	9.78718021	8.52771153
Ge	10.77083250	6.88631425	9.67202225
C	11.56003507	10.20186266	4.78273755
C	12.54036990	8.30709648	2.93745510
C	13.15571440	6.01550541	2.43619763
N	12.84403387	4.39035097	4.27097760
C	8.37479206	10.87026692	9.10497829
Ge	10.68179605	8.21690747	11.77683946
N	12.73523069	6.56580191	10.19688480
C	10.49466839	10.83306549	4.07753519
C	12.71677362	10.96288781	5.12729300
C	13.07338516	7.34812300	2.04571568
C	13.54092353	3.31998227	3.67685308
K	12.98319500	3.28524629	6.73840704
C	7.32978209	10.60136434	10.03454888
C	8.59163551	12.20412866	8.64415947
Ge	10.68120348	10.56484677	12.49341510
N	12.68980802	8.27021922	11.79843201
N	10.36009883	7.55666445	13.62200105
C	13.40211758	7.27157285	11.16580294

C	13.47607550	5.48271314	9.61952977
C	9.22987412	10.08674391	3.66998753
C	10.64300053	12.17244788	3.66798271
C	12.80400103	12.30903572	4.72325644
C	13.89138238	10.36555914	5.88849011
C	13.56116509	7.79013830	0.68302051
C	14.95715086	3.33255238	3.40953688
C	12.83280563	2.07630805	3.50034173
C	7.13568691	9.23130502	10.68067509
C	6.43809223	11.64349468	10.37344849
C	7.69084427	13.21824399	9.03292430
C	9.80657302	12.56108004	7.80032819
N	12.58308127	10.24707456	13.06247517
N	9.80530935	9.79628661	14.19234575
C	13.32166036	9.17779223	12.63107998
C	10.29074137	6.15104622	13.94225547
C	9.82839000	8.45403566	14.54224129
C	14.74321456	7.02451217	11.55848400
C	14.52092894	5.75163159	8.68979222
C	13.25936213	4.14886341	10.08043220
C	9.23879340	9.82397660	2.14745620
C	7.94776591	10.85278899	4.05836811
C	11.78890947	12.91213464	3.97516048
C	14.11879307	11.06985067	7.23946751
C	15.17493170	10.36706079	5.02796713
C	15.84176975	4.55589509	3.66562821

C	15.58929680	2.15410634	2.96875256
C	13.52489824	0.92433116	3.08426099
C	11.31861885	1.98474030	3.70746876
C	5.85181056	8.52206002	10.19682807
C	7.08244046	9.41458228	12.20878087
C	6.58812705	12.93660396	9.85496205
C	9.51842098	13.59251577	6.69691361
C	10.90744665	13.07222558	8.75339668
C	13.30432589	11.37000895	13.61229557
C	9.13968539	10.74389934	14.99213805
C	14.67315450	8.97443762	12.98998810
C	11.35600041	5.51976565	14.64753597
C	9.13394805	5.39013469	13.59762519
C	9.31044295	8.04599079	15.78741780
C	15.37222755	7.87087244	12.47918505
C	14.71506651	7.12175658	8.04382989
C	15.41252286	4.70949235	8.35068248
C	14.16003890	3.13473407	9.69145157
C	12.04467210	3.79192753	10.92462168
C	16.56172132	5.04906125	2.39081517
C	16.88337849	4.25541568	4.76876099
C	14.89588849	0.94987626	2.80602680
C	10.91562302	1.01893740	4.84705670
C	10.61054212	1.54590130	2.40517315
C	13.35113067	11.57548408	15.01967686
C	13.99007346	12.26410170	12.73554788

C	8.69505240	10.33762927	16.28841530
N	9.00657662	11.96248739	14.45332015
C	12.62077542	6.26602638	15.05526959
C	11.20755238	4.18036971	15.05701248
C	9.04660247	4.04397506	14.00159190
C	7.95945671	5.98759633	12.83635445
C	8.77740217	9.00505587	16.67904997
C	16.81548150	7.63644057	12.86663704
C	15.99873981	7.83105243	8.52815191
C	14.76875635	6.93871841	6.51571576
C	15.26255911	3.41636921	8.86914870
C	12.33326342	2.76079323	12.02819776
C	10.94366571	3.28042267	9.97191271
C	12.76682892	10.57251862	16.00864442
C	14.06650211	12.67958437	15.52369072
C	14.68874629	13.35272847	13.29357380
C	14.06815811	12.06352759	11.22170184
C	8.30971474	13.03305222	15.04709708
K	8.86748703	13.06711076	11.98591406
C	12.61175839	6.52853931	16.57785117
C	13.90291601	5.50008047	14.66681687
C	10.06162159	3.44076866	14.74970788
C	7.73217561	5.28347004	11.48526878
C	6.67578171	5.98609426	13.69668796
C	8.28960590	8.56317873	18.04178299
C	11.91170717	11.23394054	17.10764867

C	13.91587122	9.77425855	16.66745448
C	14.72668105	13.57138387	14.67433679
C	13.73985454	13.36510566	10.46135446
C	15.48351220	11.58440278	10.81560905
C	6.89351963	13.02060158	15.31457215
C	9.01785968	14.27681641	15.22294030
C	6.00889212	11.79710791	15.05923418
C	6.26140540	14.19928998	15.75475660
C	8.32579241	15.42902028	15.63843334
C	10.53207350	14.36820601	15.01588996
C	5.28905270	11.30468027	16.33439858
C	4.96717098	12.09693249	13.95602953
C	6.95481312	15.40362120	15.91674554
C	10.93531037	15.33369752	13.87612847
C	11.24002619	14.80728845	16.31817617
H	5.45379773	12.45392689	13.03214559
H	4.38092491	11.19292534	13.71045663
H	4.25491133	12.87809362	14.27494021
H	6.00675456	11.04569383	17.12838311
H	4.60542909	12.07503592	16.73319835
H	4.68642701	10.40543403	16.11631860
H	6.65340297	10.97869909	14.70032839
H	5.18478307	14.17189649	15.96162316
H	6.43517196	16.30595117	16.25546930
H	8.88408410	16.36390275	15.76858914
H	10.88412067	13.35675704	14.74353714

H	12.33484782	14.77476474	16.19140531
H	10.95878938	15.83898617	16.59606175
H	10.97118853	14.15044465	17.16067996
H	12.03185601	15.34568836	13.75447830
H	10.51236392	15.05021679	12.89338118
H	10.60006515	16.36607211	14.08178337
H	12.12458004	9.89021917	15.42702470
H	14.62967021	10.45957145	17.15885943
H	13.52135974	9.09299121	17.43847076
H	14.47391403	9.17524525	15.92919925
H	12.52240946	11.87721455	17.76654859
H	11.10305612	11.84506168	16.68090829
H	11.44760639	10.45912419	17.73982900
H	15.22771480	14.03622248	12.62923807
H	15.28367788	14.42035759	15.08528103
H	14.11709029	12.82787878	16.60754119
H	13.34262409	11.28519618	10.91686484
H	15.74162470	10.63161645	11.30318376
H	15.54359991	11.44267594	9.72368852
H	16.24380830	12.33356669	11.10108679
H	13.73166510	13.17738876	9.37618290
H	12.75778750	13.77306036	10.75081696
H	14.49661658	14.14633114	10.65088125
H	6.56513832	10.16855196	7.59811279
H	4.83210587	8.36663543	4.83290558
H	4.35568125	8.16198867	6.53229292

H	4.76789255	9.78255033	5.92506039
H	5.71912323	7.56728435	10.73270576
H	4.96175771	9.14337920	10.40455199
H	5.88276188	8.31139435	9.11780978
H	6.83243929	6.53269118	14.64151934
H	5.83837740	6.46224330	13.15602619
H	6.36179667	4.95724647	13.94642200
H	8.61004579	5.41003768	10.82877604
H	7.53109746	4.20480467	11.61244706
H	6.86132496	5.72452057	10.97009150
H	8.22240949	7.03384008	12.61269319
H	8.14957634	3.46973600	13.74354317
H	9.96396140	2.40242604	15.08473396
H	12.01318775	3.70911770	15.63027422
H	12.61266863	7.22459520	14.50861753
H	14.79170460	6.10840861	14.90628010
H	13.99587420	4.55046679	15.22363341
H	13.93147428	5.27622483	13.58879227
H	13.55387525	7.00129388	16.89817809
H	11.77386639	7.18308145	16.86819851
H	12.50969434	5.57782269	17.13151240
H	9.33030625	6.98861395	16.05468347
H	8.28146260	11.09995313	16.95306785
H	9.11000071	8.11171042	18.62765264
H	7.87897118	9.40567040	18.62041074
H	7.50341657	7.79279980	17.95070825

H	15.99108468	2.63383261	8.62798400
H	16.25879127	4.92778407	7.69239101
H	14.02225051	2.12088874	10.08159475
H	11.65883765	4.72263270	11.38011168
H	13.14987048	3.08759992	12.69205972
H	11.43474855	2.61909694	12.64714278
H	12.60465377	1.77526518	11.60770574
H	11.23806733	2.31751463	9.50858797
H	10.00455453	3.11371709	10.52292730
H	10.74865929	4.02174883	9.17638018
H	15.28565463	6.18402799	11.12659985
H	15.15397046	9.68410918	13.66463744
H	17.49555004	8.18224081	12.18684473
H	17.02116471	7.99349789	13.88879227
H	17.07999493	6.56820183	12.80858222
H	17.46963740	5.15926154	5.01490291
H	16.39664697	3.89791522	5.69239459
H	17.59564355	3.47441329	4.44947842
H	17.24536155	4.27893130	1.99160735
H	15.84407686	5.30854280	1.59694240
H	17.16434832	5.94816633	2.60947780
H	15.19723122	5.37409723	4.02495589
H	16.66593500	2.18161206	2.76198547
H	15.41553716	0.04773166	2.46682206
H	12.96661444	-0.01048241	2.95358321
H	10.96651156	2.99608793	3.98011771

H	10.89182936	0.51429628	2.12699507
H	9.51573588	1.57828040	2.53210466
H	10.87919631	2.20297426	1.56278976
H	11.33869150	1.30225465	5.82979848
H	9.81909553	1.00680648	4.96886497
H	11.25096179	-0.01334708	4.64111206
H	10.74773243	4.50836636	2.04375422
H	9.32822156	4.47612571	0.95832090
H	10.40276777	5.89441071	0.98511585
H	7.73388607	3.52500176	2.11739764
H	6.56777426	1.93205793	3.63954386
H	6.62387594	2.31587198	6.09562919
H	9.72597393	6.46285778	3.29807323
H	8.32872421	7.25994657	1.28684997
H	7.22073998	5.89312967	1.56656266
H	7.37644266	7.17741370	2.79627856
H	8.50853954	5.06708820	7.80816975
H	6.30762204	4.90916812	9.00138856
H	6.10949147	5.72036653	7.42197207
H	5.60750223	4.01832841	7.62390335
H	9.09373417	2.57927931	7.97410852
H	8.11975021	3.17471529	9.34876115
H	7.35495648	2.20575388	8.07398744
H	13.56926102	5.25324913	1.77144031
H	12.52047802	9.36449554	2.67028326
H	13.97172440	6.94769024	0.10427520

H	12.74078091	8.24173361	0.09723425
H	14.34740775	8.56045405	0.77416654
H	10.07661672	9.16932307	1.85716098
H	8.29661744	9.35125193	1.82726053
H	9.34089763	10.77459506	1.59363270
H	9.23800535	9.12826775	4.21680236
H	7.85475624	11.80224913	3.50129943
H	7.05894938	10.24440729	3.81915556
H	7.91929443	11.07695006	5.13633030
H	9.83729044	12.64362460	3.09476559
H	11.88648891	13.95046460	3.64007245
H	13.70100600	12.88334967	4.98121454
H	13.62848829	9.31933138	6.11229923
H	16.01246661	9.89107041	5.56856368
H	15.01817722	9.82032697	4.08323040
H	15.48876439	11.39590963	4.77804639
H	10.41711895	13.73421002	6.07823487
H	8.70193996	13.26598287	6.03276304
H	9.24708323	14.57799256	7.11756074
H	7.82869098	14.23207511	8.64277271
H	5.85950882	13.71911931	10.09590439
H	5.59169227	11.42520563	11.03157666
H	11.84666673	13.23900489	8.20259842
H	10.61303269	14.03503003	9.21693235
H	11.10230749	12.33067336	9.54876110
H	10.19237097	11.63042660	7.34470102

H	8.01425402	8.60947645	10.42029671
H	7.99780624	9.89101310	12.59182667
H	6.22281747	10.03705279	12.50520395
H	6.96829899	8.44965529	12.72141609
H	6.69692256	6.66801429	5.06072581
H	13.83639631	7.74348587	8.30409366
H	15.62865684	6.31657048	6.21941391
H	13.85364368	6.46204533	6.13236399
H	14.88272928	7.90375673	6.00326464
H	16.88889357	7.20979797	8.32066474
H	16.13153149	8.78589325	7.99241376
H	15.96740241	8.04162000	9.60718337
H	14.31955790	12.14855865	7.11218186
H	13.24108170	10.94306986	7.89613933
H	14.98987889	10.62907364	7.75448355

5A

**Electronic energy (BP86)**	**−7183.692266**
**Zero-point correction**	0.359021
**Thermal correction to energy**	0.387960
**Thermal correction to enthalpy**	0.388904
**Thermal correction to Gibbs free energy**	0.298970

N	3.06866748	-0.35726944	-0.43215143
C	2.88561066	0.59519146	0.56464163
Ge	1.16309343	-0.40439949	-0.87057006
C	3.74540327	1.43484948	1.27637602
N	1.52871528	0.67544606	0.75496527

N	0.72133275	-2.07581536	0.08900817
C	3.14770938	2.38243873	2.15155905
C	0.91153229	1.62897471	1.50414223
C	-0.58269707	-2.22011562	0.46196195
C	4.04108413	3.29751115	2.96048719
C	1.75070620	2.49484968	2.25787848
N	-0.44921482	1.63319827	1.41073200
C	-1.29521304	-3.41709438	0.74750661
N	-1.28053517	-1.05518928	0.54808891
Ge	-1.20503214	0.78069515	-0.20506619
C	-2.67049911	-3.36165528	1.03228801
C	-2.63741899	-0.97545916	0.73862909
N	-2.96741486	0.35982783	0.53187683
C	-3.37743072	-2.12843854	1.01052460
C	-3.42694563	-4.63414681	1.34591088
H	-0.78445357	-4.38110970	0.70568483
H	-4.45610344	-2.09137727	1.17809191
H	-2.79979877	-5.52759504	1.20057940
H	-3.77951690	-4.63370163	2.39323528
H	-4.32117959	-4.73355323	0.70623598
H	4.82941958	1.38354337	1.15280006
H	1.31140260	3.27197152	2.88641963
H	4.77405330	3.81121416	2.31424853
H	3.46053661	4.06379116	3.49752697
H	4.61837550	2.72335187	3.70761259
C	-4.30787780	0.73880168	0.10943032

H	-4.62162017	0.23581839	-0.82658004
H	-5.04982515	0.50429674	0.89558128
H	-4.33723035	1.82724775	-0.05670983
C	-1.21212155	2.62862573	2.15586423
H	-0.93816592	2.61427260	3.22649013
H	-1.06523393	3.65679799	1.76972664
H	-2.28050008	2.37043899	2.08290950
C	4.25534394	-0.33595057	-1.27485643
H	4.39459658	0.63140424	-1.79675035
H	5.16471822	-0.54413721	-0.68050952
H	4.16986515	-1.12893348	-2.03450294
C	1.55706430	-3.26280626	-0.05546462
H	1.52671361	-3.87973882	0.86105116
H	1.25694110	-3.89539669	-0.91429859
H	2.59885432	-2.93601266	-0.20147953
Ge	-0.25262240	0.78924537	-2.43281604

TS_{5A5B}

**Electronic energy (BP86)**	**−7183.680704**
**Zero-point correction**	0.358531
**Thermal correction to energy**	0.386944
**Thermal correction to enthalpy**	0.387888
**Thermal correction to Gibbs free energy**	0.298822

N	14.52252880	8.85640310	3.36896072
C	14.36415357	9.85807472	4.28929673
Ge	12.54210983	8.70715659	3.09113063
C	15.21959314	10.68270126	5.03083910

N	13.00023080	10.00002398	4.43776158
N	12.33355007	6.96890712	3.86263868
C	14.62244006	11.59216306	5.94378707
C	12.37904529	10.89091632	5.25751076
C	11.01831484	6.84158506	4.29099837
C	15.51889500	12.45888888	6.80196673
C	13.22583996	11.70588801	6.05984951
N	11.02759867	10.89432469	5.16537542
C	10.28530746	5.67178346	4.57322984
N	10.44823362	8.06216292	4.36173628
Ge	10.11280446	10.53938085	3.43346832
C	8.90547513	5.82171789	4.84666659
C	9.11184754	8.21072907	4.46384110
N	8.67361438	9.49712535	4.18213292
C	8.29783582	7.08996055	4.77618611
C	8.07484646	4.60295882	5.18915317
H	10.74546597	4.68095190	4.53716873
H	7.21972179	7.19438595	4.92037915
H	8.33167173	3.74596895	4.54346378
H	8.25566796	4.28677600	6.23284517
H	6.99651536	4.80261035	5.08331105
H	16.30520990	10.60719582	4.93792102
H	12.78978124	12.45115522	6.72758229
H	16.29383629	12.95541261	6.19293875
H	14.94785004	13.23546496	7.33428200
H	16.04520506	11.84827400	7.55771884

C	7.25606092	9.73343575	3.94251493
H	6.84390482	9.05477980	3.16911432
H	6.65677965	9.60620149	4.86517543
H	7.11824533	10.77063390	3.59722725
C	10.25654011	11.73028203	6.08248787
H	10.60307802	11.58671724	7.12156513
H	10.31081782	12.80779602	5.83264217
H	9.20231917	11.41297385	6.02924735
C	15.73952884	8.68422808	2.60069089
H	16.02113962	9.59306270	2.03130902
H	16.59634798	8.40779494	3.24604274
H	15.59498011	7.86671660	1.87660539
C	13.18441189	5.80863261	3.66954269
H	13.21757850	5.19382241	4.58876266
H	12.84996886	5.16204819	2.83303279
H	14.21012473	6.15126350	3.46305282
Ge	10.96185782	9.46491656	1.39032792

5B

**Electronic energy (BP86)**	**−7183.720374**
**Zero-point correction**	0.359473
**Thermal correction to energy**	0.387983
**Thermal correction to enthalpy**	0.388927
**Thermal correction to Gibbs free energy**	0.301013

N	3.12698079	-0.28533809	-0.69452451
C	2.88140458	0.58911506	0.35716538
Ge	1.24729737	-0.44414700	-1.13442311

C	3.68452663	1.30710829	1.23927908
N	1.51159216	0.67324181	0.43279828
N	0.86911520	-2.20808904	-0.45325494
C	3.00616200	2.08765129	2.22137832
C	0.80754811	1.47316858	1.28798250
C	-0.45603040	-2.31248872	-0.10472032
C	3.83057591	2.85828382	3.22960385
C	1.60944438	2.17163220	2.25207057
N	-0.53616223	1.52020594	1.14298643
C	-1.03505684	-3.30383790	0.71410864
N	-1.23321600	-1.28612562	-0.57338254
Ge	-1.56301567	1.73028524	-0.67527673
C	-2.37882847	-3.15617723	1.11292707
C	-2.45660506	-0.99756379	-0.01343121
N	-2.91663106	0.25583926	-0.28841962
C	-3.08836691	-1.99005720	0.77615159
C	-3.04180671	-4.23596617	1.93903530
H	-0.43883531	-4.14796258	1.06523399
H	-4.09303697	-1.82054396	1.16788419
H	-3.46358033	-5.02056211	1.28416991
H	-2.32281042	-4.72790385	2.61495723
H	-3.86886999	-3.83298674	2.54553875
H	4.77489529	1.26908897	1.19454072
H	1.12732848	2.81914158	2.98636923
H	4.54784010	3.52860074	2.72392671
H	3.19860617	3.46869801	3.89302254

H	4.42361839	2.17080786	3.85893786
C	-4.26471685	0.60652488	0.14643397
H	-5.02770356	-0.03890648	-0.32954943
H	-4.39332839	0.52975857	1.24709017
H	-4.46738086	1.64793740	-0.14927550
C	-1.27274493	2.27412772	2.16236862
H	-0.99596526	1.94686780	3.18224387
H	-1.12219771	3.37000871	2.08601158
H	-2.34809167	2.07865068	2.02759375
C	4.41587013	-0.40649495	-1.34571019
H	4.77289867	0.55125777	-1.77467366
H	5.18748736	-0.77344513	-0.64165377
H	4.33945108	-1.13910368	-2.16414824
C	1.82500115	-3.16451748	0.08401780
H	1.76674386	-3.23006252	1.18901191
H	1.66535293	-4.17663104	-0.33368311
H	2.84038288	-2.83391027	-0.17976215
Ge	-0.85200432	-0.10990667	-2.24918399

TS_{5B5C}

**Electronic energy (BP86)**	**−7183.691886**
**Zero-point correction**	0.358384
**Thermal correction to energy**	0.386201
**Thermal correction to enthalpy**	0.387145
**Thermal correction to Gibbs free energy**	0.300337

N	4.06955632	4.01508501	4.48417629
C	2.82514116	4.49903655	4.27705734

Ge	5.19837111	4.50660204	6.05102775
C	1.99242443	4.14733884	3.18658667
N	2.35143787	5.41984312	5.21219412
Ge	3.15532536	5.58181692	7.01045240
N	5.94049273	6.27120729	5.39214683
C	0.76520989	4.79941843	2.99223871
C	1.21758474	6.18692317	4.93573220
Ge	2.71423981	8.13155769	6.43323528
N	4.20952688	7.87404486	4.93457087
C	5.47388577	7.36697727	4.72174101
C	-0.13414746	4.39706450	1.84561568
C	0.38672223	5.84784729	3.84873730
N	1.04105918	7.23290751	5.78059124
N	2.64170136	9.45717475	4.91737272
C	3.84531396	9.09541606	4.38019188
C	6.29897079	8.04670042	3.76626215
C	4.66497692	9.81215473	3.49954551
C	5.90155518	9.23500656	3.14707268
C	6.78400508	9.89543427	2.11195321
H	4.35195170	10.78607683	3.11649038
H	6.34616917	9.79650495	1.10223959
H	6.88910169	10.97602580	2.31134319
H	7.79081491	9.44947633	2.08935492
H	7.24614044	7.58915879	3.47609572
H	-0.51275295	6.43212549	3.64795975
H	2.32427644	3.38500106	2.48047654

H	-0.68150526	5.26292601	1.43808707
H	0.43579390	3.92769430	1.02771371
H	-0.88871920	3.66452214	2.18654604
C	7.40601346	6.13793649	5.45584125
H	7.81307533	5.57795499	4.58922211
H	7.90292550	7.12298015	5.51733170
H	7.66709602	5.55950231	6.35667355
C	4.61217675	3.07208724	3.51018516
H	4.02815523	2.13135943	3.46708258
H	4.64496827	3.50581407	2.49088412
H	5.63982194	2.81631889	3.81199190
C	-0.13696235	8.07473205	5.62289039
H	-0.11129818	8.66334855	4.68425839
H	-1.06361631	7.47262983	5.64134275
H	-0.18581965	8.78052591	6.47057710
C	2.18051854	10.83030139	4.82489543
H	2.95278954	11.57157468	5.11690321
H	1.85263619	11.07324576	3.79480429
H	1.31169115	10.96615976	5.48890223

5C

**Electronic energy (BP86)**	**−7183.778601**
**Zero-point correction**	0.361619
**Thermal correction to energy**	0.389173
**Thermal correction to enthalpy**	0.390118
**Thermal correction to Gibbs free energy**	0.303544

N	4.05519523	4.14511760	4.83256782

C	2.86532977	4.61252056	4.37362128
Ge	5.23160945	4.98399788	6.21608867
C	2.07700787	3.95864859	3.39659581
N	2.40201840	5.79991759	4.91708959
Ge	3.50326341	6.72132927	6.23313897
N	6.15514512	6.23514402	4.95753808
C	0.83795056	4.49628720	3.01995995
C	1.15588869	6.32160836	4.60836669
Ge	1.79117049	8.41251504	6.69638523
N	4.51230236	7.89936419	5.05534293
C	5.72829694	7.46050681	4.55632547
C	0.01971278	3.82713530	1.93799276
C	0.36798223	5.66756692	3.63130420
N	0.76652395	7.43359984	5.28418906
N	2.86679578	9.52208262	5.42627850
C	4.01920489	9.16888396	4.80020836
C	6.44806944	8.30612835	3.67875567
C	4.73967275	10.01444660	3.92302877
C	5.94793212	9.57726232	3.36166449
C	6.73348003	10.49296275	2.44928210
H	4.35254986	11.00453226	3.68170591
H	7.32016260	9.92135358	1.71188982
H	6.07152019	11.18699289	1.90645994
H	7.44518363	11.10490644	3.03300440
H	7.39018730	7.96727197	3.24749294
H	-0.60282446	6.07722996	3.35196156

H	2.43602576	3.03904238	2.93429685
H	0.21009837	2.74237092	1.89942902
H	-1.06000741	3.98659799	2.09112905
H	0.27626917	4.24250643	0.94629111
C	7.44770320	5.77399918	4.45670394
H	7.43885023	5.61902184	3.35945769
H	8.26591590	6.47985363	4.70100910
H	7.68291593	4.80848584	4.93307018
C	4.53904907	2.87986358	4.28552037
H	3.80981322	2.05809534	4.42755686
H	4.77221847	2.95415948	3.20464246
H	5.46563129	2.60222799	4.81358210
C	-0.55580648	7.97696613	4.98341782
H	-0.62581337	8.35327931	3.94341432
H	-1.35885477	7.22933853	5.13676663
H	-0.74934137	8.82243653	5.66326280
C	2.35373777	10.86854969	5.18543651
H	3.09614722	11.64995012	5.44152092
H	2.04482051	11.01527239	4.13133321
H	1.46808505	11.02420871	5.82251383

6. References

(1) Fulmer, G. R.; Miller, A. J. M.; Sherden, N. H.; Gottlieb, H. E.; Nudelman, A.; Stoltz, B. M.; Bercaw, J. E.; Goldberg, K. I. NMR Chemical Shifts of Trace Impurities: Common Laboratory Solvents, Organics, and Gases in Deuterated Solvents Relevant to the Organometallic Chemist. *Organometallics* **2010**, *29*, 2176−2179.

(2) Fujie, Y.; Hanaki, N.*Jpn. Kokai Tokyo Koho*; 2004, JP 2004175789 A 20040624.

(3) Wey, Y.-T.; Yang, F.-S.; Yu, H.-C.; Kuo, T.-S.; Tsai, Y.-C. Synthesis and Characterization of an Eclipsed Digermylene as a Building Block to Construct a Cyclic Octagermylene. *Angew. Chem. Int. Ed.* **2017**, *56*, 15108−15112.

(4) Ottmers, D. M.; Rase, H. F. Potassium Graphites Prepared by Mixed-Reaction Technique. *Carbon* **1966**, *4*, 125−127.

(5) Duwell, E. J.; Baenziger, N. C. The Crystal Structures of KHg and KHg_2. *Acta Cryst.* **1955**, *8*, 705−710.

(6) *DENZO-SMN*, Otwinowski & Minor, **1997**.

(7) Blessing, R. H. An empirical correction for absorption anisotropy. *Acta Cryst*. **1995**, *A51*, 33−38.

(8) *SHELXTL*: Structure analysis program, version 6.10; Bruker-axs, Madison, WI, **2000**.

(9) *SHELXL-97*, Sheldrick, G. M. **1997**, University of Göttingen, Germany

(10) Sheldrick, G. M. *SHELXT* − Integrated space-group and crystalstructure determination. *Acta Cryst.* **2015**, *A71*, 3−8.

(11) Sheldrick, G. M. Crystal structure refinement with *SHELXL*. *Acta Cryst.* **2015**, *C71*, 3−8.

(12) Dolomanov, O. V.; Bourhis, L. J.; Gildea, R. J.; Howard, J. A. K.; Puschmann, H. *OLEX2*: a complete structure solution, refinement and analysis program. *J. Appl. Crystallogr.* **2009**, *42*, 339−341.

(13) Frisch, M. J.; Trucks, G. W.; Schlegel, H. B.; Scuseria, G. E.; Robb, M. A.; Cheeseman, J. R.; Scalmani, G.; Barone, V.; Petersson, G. A.; Nakatsuji, H.; Li, X.; Caricato, M.; Marenich, A. V.; Bloino, J.; Janesko, B. G.; Gomperts, R.; Mennucci, B.; Hratchian, H. P.; Ortiz, J. V.; Izmaylov,

A. F.; Sonnenberg, J. L.; Williams-Young, D.; Ding, F.; Lipparini, F.; Egidi, F.; Goings, J.; Peng, B.; Petrone, A.; Henderson, T.; Ranasinghe, D.; Zakrzewski, V. G.; Gao, J.; Rega, N.; Zheng, G.; Liang, W.; Hada, M.; Ehara, M.; Toyota, K.; Fukuda, R.; Hasegawa, J.; Ishida, M.; Nakajima, T.; Honda, Y.; Kitao, O.; Nakai, H.; Vreven, T.; Throssell, K.; Montgomery, Jr., J. A.; Peralta, J. E.; Ogliaro, F.; Bearpark, M. J.; Heyd, J. J.; Brothers, E. N.; Kudin, K. N.; Staroverov, V. N.; Keith, T. A.; Kobayashi, R.; Normand, J.; Raghavachari, K.; Rendell, A. P.; Burant, J. C.; Iyengar, S. S.; Tomasi, J.; Cossi, M.; Millam, J. M.; Klene, M.; Adamo, C.; Cammi, R.; Ochterski, J. W.; Martin, R. L.; Morokuma, K.; Farkas, O.; Foresman, J. B.; Fox D. J. *Gaussian, Inc., Wallingford CT*, **2016**.

(14) Perdew, J. P. Density-functional approximation for the correlation energy of the inhomogeneous electron gas. *Phys. Rev. B* **1986**, *33*, 8822−8824.

(15) Becke, A. D. Density-functional exchange-energy approximation with correct asymptotic behavior. *Phys. Rev. A* **1988**, *38*, 3098−3100.

(16) Fukui, K. Formulation of the reaction coordinate. *J. Phys. Chem.* **1970,** *74*, 4161−4163.

(17) Fukui, K. The path of chemical reactions - the IRC approach. *Acc. Chem. Res.* **1981,** *14*, 363−368.

(18) Ditchfield, R.; Hehre, W. J.; Pople, J. A. Self-consistent molecular-orbital methods. ix. an extended gaussian-type basis for molecular-orbital studies of organic molecules. *J. Chem. Phys.* **1971**, *54*, 724−728.

(19) Hehre, W. J.; Ditchfield, R.; Pople, J. A. Self-consistent molecular orbital methods. xii. further extensions of gaussian-type basis sets for use in molecular orbital studies of organic molecules. *J. Chem. Phys.* **1972**, *56*, 2257−2261.

(20) Hariharan, P. C.; Pople, J. A. The influence of polarization functions on molecular orbital hydrogenation energies. *Theor. Chim. Acta* **1973**, *28*, 213−222.

(21) Francl, M. M.; Pietro, W. J.; Hehre, W. J.; Binkley, J. S.; Gordon, M. S.; DeFrees, D. J.; Pople, J. A. Self-consistent molecular orbital methods. XXIII. A polarization-type basis set for second-row elements. *J. Chem. Phys.* **1982**, *77*, 3654−3665.

(22) Gordon, M. S.; Binkley, J. S.; Pople, J. A.; Pietro, W. J.; Hehre, W. J. Self-consistent molecular-

orbital methods. 22. Small split-valence basis sets for second-row elements. *J. Am. Chem. Soc.* **1982**, *104*, 2797−2803.

(23) Rassolov, V. A.; Ratner, M. A.; Pople, J. A.; Redfern, P. C.; Curtiss, L. A. 6-31G* basis set for third-row atoms. *J. Comput. Chem.* **2001**, *22*, 976−984.

(24) Weigenda, F.; Ahlrichsb, R. Balanced basis sets of split valence, triple zeta valence and quadruple zeta valence quality for H to Rn: Design and assessment of accuracy. *Phys. Chem. Chem. Phys.* **2005**, *7*, 3297−3305.

(25) Lu, T.; Chen, F. Multiwfn: a multifunctional wavefunction analyzer. *J. Comput. Chem.* **2012**, *33*, 580−592.

(26) Reed, A. E.; Curtiss, L. A.; Weinhold, F. Intermolecular interactions from a natural bond orbital, donor-acceptor viewpoint. *Chem. Rev.* **1988**, *88*, 899–926.

(27) Becke, A. D.; Edgecombe, K. E. A simple measure of electron localization in atomic and molecular systems. *J. Chem. Phys.* **1990**, *92*, 5397–5403.

(28) Mitoraj, M. P.; Michalak, A.; Ziegler, T. A Combined Charge and Energy Decomposition Scheme for Bond Analysis. *J. Chem. Theory Comput.* **2009**, *5*, 962−975.
